# Synthesis and molecular modeling studies of cholinesterase inhibitor dispiro[indoline-3,2′-pyrrolidine-3′,3′′-pyrrolidines]†

**DOI:** 10.1039/d0ra03064c

**Published:** 2020-06-08

**Authors:** M. Adel Youssef, Siva S. Panda, Riham A. El-Shiekh, ElSayed M. Shalaby, Dalia R. Aboshouk, Walid Fayad, Nehmedo G. Fawzy, Adel S. Girgis

**Affiliations:** Department of Chemistry, Faculty of Science, Helwan University Helwan Egypt; Department of Chemistry and Physics, Augusta University Augusta GA 30912 USA; Department of Pharmacognosy, Faculty of Pharmacy, Cairo University Cairo 11562 Egypt; X-Ray Crystallography Lab., Physics Division, National Research Centre Dokki Giza 12622 Egypt; Department of Pesticide Chemistry, National Research Centre Dokki Giza 12622 Egypt girgisas10@yahoo.com; Drug Bioassay-Cell Culture Laboratory, Pharmacognosy Department, National Research Centre Dokki Giza 12622 Egypt

## Abstract

A set of dispiro[indoline-3,2′-pyrrolidine-3′,3′′-pyrrolidines] 8a–l was regioselectively synthesized utilizing multi-component azomethine cycloaddition reaction of 3-(arylmethylidene)pyrrolidine-2,5-diones 5a–e, isatins 6a–c and sarcosine 7. Single crystal X-ray studies of 8c add conclusive support for the structure. Compounds 8e and 8g reveal cholinesterase inhibitory properties with promising efficacy against both AChE and BChE and were found to be more selective towards AChE than BChE as indicted by the selectivity index like Donepezil (a clinically used cholinesterase inhibitory drug). Molecular modeling studies assist in understanding the bio-observations and identifying the responsible parameters behind biological properties.

## Abstract 2

Dispiro[indoline-3,2′-pyrrolidine-3′,3′′-pyrrolidines] were regioselectively synthesized revealing cholinesterase (AChE, BChE) inhibitory properties.

## Graphical abstract

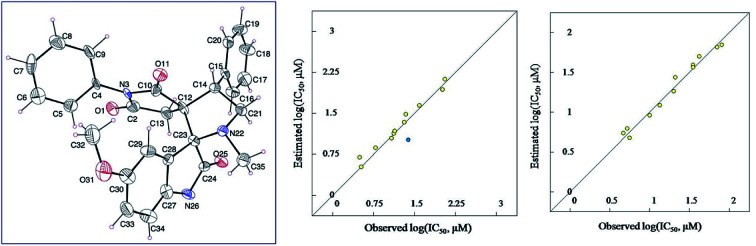

## Introduction

**Fig. 1:**
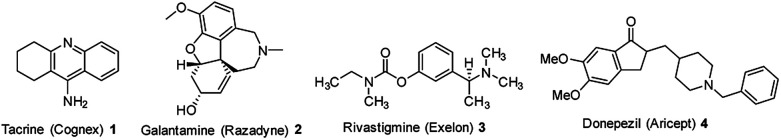
Cholinesterase inhibitor drugs useful for AD.

Dementia is one of the most serious health problems for older people. About 50 million people are suffering from dementia globally with 10 million new cases yearly according to the WHO (World Health Organization).^1^ Alzheimer's disease (AD) represents the most common cause of dementia (60–70%).^1^ AD is a fatal chronic neurodegenerative disease associated with memory impairment and language deficits besides high degeneration of cholinergic neurons of the central nervous system.^2,3^ Although no cure has been discovered for AD, a few pharmacological targets have been rationalized. Reduction of formation and aggregation of pathological hallmarks of AD (insoluble amyloid-β oligomers and tau neurofibrillary tangles) is a pharmacological target for AD. Other targets include modulation of neurotransmitter signals (cholinesterase inhibitors and *N*-methyl-d-aspartate receptor blockers).^4^ Although the full mechanism of AD is not well elucidated yet, extensive studies explained that the brain of AD patients suffers from cholinergic neuron damage. This is why acetylcholine (AC) level is considered an important therapeutic target of AD. AC is an important brain neurotransmitter with major roles in memory and maintaining consciousness.^5^ Acetylcholinesterase (AChE) is a catabolic enzyme capable for hydrolysis of AC. Butyrylcholinesterase (BChC) also regulates the AC levels. This is why inhibition of both cholinesterases is useful for AD patients.^2,6^ Tacrine (Cognex) 1 was the first cholinesterase inhibitor approved drug^7^ (Fig. 1). However, due to many clinically adverse effects including elevated liver transaminase levels it has been discontinued in many countries.^8,9^ Meanwhile, many researchers are still interested in this compound for developing analogs of lesser side effects.^9–11^

Currently, many cholinesterase inhibitors are clinically used as AD drugs of which Galantamine (Razadyne) 2 (approved by FDA “Food and Drug Administration” on 28 Feb. 2001) for mild and moderate AD.^4,12,13^ Rivastigmine (Exelon) 3 (approved by FDA in 21 April 2000) is also useful for mild and moderate dementia patients caused by AD or Parkinson's disease.^4,14,15^ Donepezil (Aricept) 4 is also a cholinesterase inhibitor approved by FDA on 25 Nov. 1996 for AD.^4,16,17^ Currently, available drugs are used to manage and prevent progress of the disease over time but unfortunately not able to cure. Additionally, the drugs lack long term efficacy and also associated with severe side effects. This is why urgent need of effective anti-AD agents are still compelling.^18^

The present study is focused on the construction of novel dispiro[indoline-3,2′-pyrrolidine-3′,3′′-pyrrolidines] and exploring their cholinesterase properties. Rational for the targeted chemical scaffold is based on the bio-isosteric form of the indanyl nucleus of Donepezil 4 and the indolyl heterocycle of the targeted agents.^19^ Additionally many natural and synthetic indole containing-compounds show promising cholinesterase inhibitory properties^20–23^ including mono- and bis-spiro-indoles.^24–27^ The biologically active spiro-indoles developed by our group also prompted the current study.^28–30^

## Results and discussion

### Chemistry

**Scheme 1:**
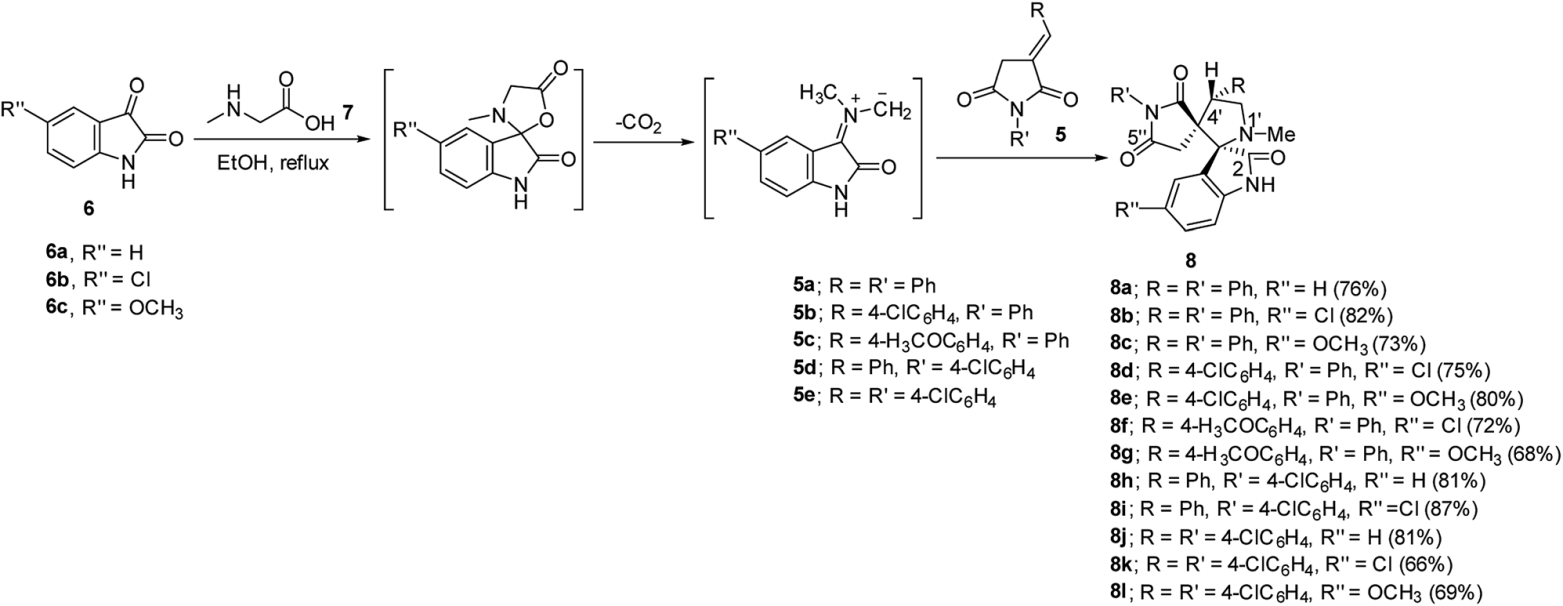
Synthetic route towards the targeted dispiro[indoline-3,2′-pyrrolidine-3′,3′′-pyrrolidine]-2,2′′,5′′-triones 8a–l.

**Fig. 2:**
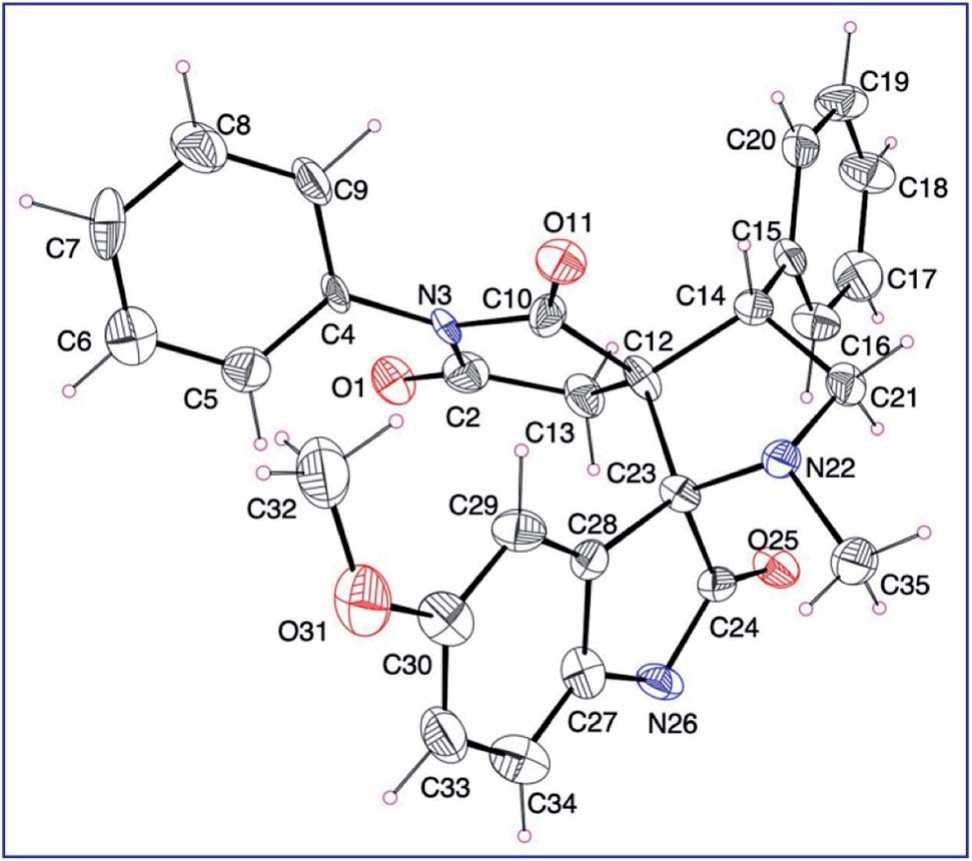
An ORTEP view of 8c showing the atom-numbering scheme. H atoms are shown as small spheres of arbitrary radii.

Synthetic route towards the targeted dispiro[indoline-3,2′-pyrrolidine-3′,3′′-pyrrolidine]-2,2′′,5′′-triones 8a–l is depicted in Scheme 1. Azomethine ylides generated from the reaction of refluxing isatins 6a–c and sarcosine 7 in ethanol reacted regioselectively with 3-(arylmethylidene)pyrrolidine-2,5-diones 5a–e^31–33^ affording solely the corresponding dispiro analogs 8a–l (TLC monitor). The non-stabilized azomethine ylide is formed *in situ* due to the applied reaction conditions (refluxing ethanol) through CO_2_ elimination from spiro-oxazalidinone. The latter is formed *via* condensation of amino acid (sarcosine) with isatin(s).^34^ The IR spectrum of compound 8a (example of the synthesized compounds) shows strong bands at *ν* = 1786, 1713 cm^−1^ assignable for the stretching vibration of carbonyl groups. ^1^H-NMR spectrum of 8a reveals the diastereotopic methylene protons of pyrrolidinedionyl H_2_C-4′′ and pyrrolidinyl H_2_C-5′ at *δ*_H_ = 2.37, 2.71 and 3.49, 3.84, respectively. The methine pyrrolidinyl HC-4′ is shown as a triplet signal at *δ*_H_ = 4.39. ^13^C-NMR spectrum of 8a reveals the pyrrolidinedionyl CH_2_ (C-4′′) and pyrrolidinyl CH_2_ (C-5′) at *δ*_C_ = 36.7, 58.4, respectively. The spiro carbons are shown at *δ*_C_ = 61.0, 77.6 assignable for C-3′ (C-3′′) and C-3 (C-2′), respectively. The methyl and methine (C-4′) carbons are revealed at *δ*_C_ = 34.6, 48.4, respectively. Additionally the carbonyl carbons are exhibited at *δ*_C_ = 173.0, 177.1 and 177.5. HSQC of compounds 8b and 8g support these interpretations (ESI Fig. S1–S38† show the spectral charts of the synthesized compounds). Single crystal X-ray study of compound 8c supports the structure (Fig. 2).

### X-ray studies

The ORTEP view of compound 8c is shown in Fig. 2. The compound is in the monoclinic system and space group *P*2_1_/*c* with four molecules in the unit cell and one molecule in the asymmetric unit of the crystallized form. Two spiro linkages exist in 8c attaching the central pyrrolidine ring to the pyrrolidinedione at C12 and to the indolyl heterocycle at C23. In general, the geometric parameters including both bond lengths and angles (ESI Tables S1–S3†) are in good agreement with the pre-determined structures having similar rings and moieties.^35,36^

**Fig. 3:**
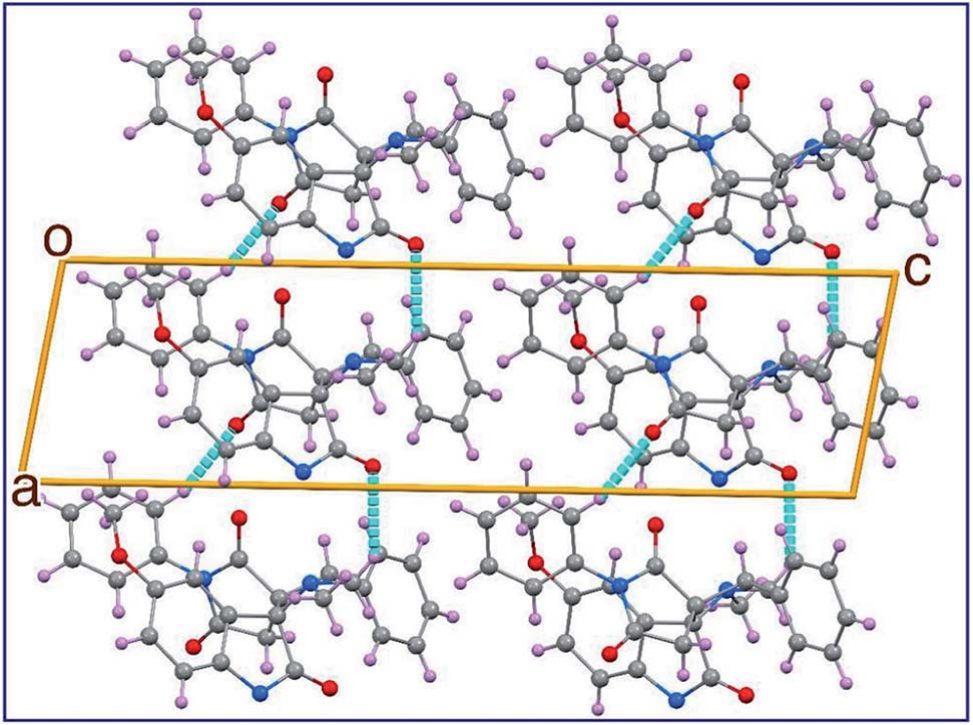
Crystal packing in the unit cell of 8c showing some hydrogen-bond interactions as dashed lines.

The two phenyl rings (C4 → C9 and C15 → C20) as well as the indolyl heterocycle are planar conformations. The two pyrrolidinyl rings (C2–N3–C10–C12–C13) and (C12–C14–C21–N22–C23) are envelope conformations with the flap atoms being C12, lies 0.223 (3) Å out of the mean plane of the remaining four atoms, and N22, lies 0.662 (4) Å out of the mean plane of the remaining atoms, respectively. The sum of angles around the N22 atom is approximately 330° confirming its sp^3^ hybridization. The C14 and C23 atoms occupy axial and equatorial positions with respect to the pyrrolidinedione. In the crystal, molecules are linked together by set of intermolecular C–H⋯O hydrogen-bonding interactions forming supramolecular assemblies (Fig. 3 and Table 1).

**Table 1:** Hydrogen-bond geometry (Å, °) for compound 8c^a^

D–H⋯A	D–H	H⋯A	D⋯A	D–H⋯A
C9–H91⋯O1^a^	0.95(3)	2.40(5)	3.308(8)	161(2)
C21–H212⋯O25^a^	0.95(2)	2.56(3)	3.287(5)	133(4)

a Symmetry codes: −1 + *x*, *y*, *z*.

### Biological studies

#### Cholinesterase inhibitory properties

AChE and BChE inhibitory properties of the synthesized dispiro-compounds 8a–l along with Donepezil (standard reference) were presented in Table 2. From the observed experimental data it has been cleared that compounds 8e and 8g are superior among all the synthesized molecules with promising efficacy against both AChE and BChE (IC_50_ = 3.35, 5.63; 3.15, 4.74 μM for 8e and 8g against AChE and BChE, respectively). Compound 8h also shows good cholinesterase properties (IC_50_ = 6.27, 5.34 μM against AChE and BChE, respectively). Donepezil seems more selective towards AChE rather than BChE (SI_(BChE/AChE)_ = 1.31) due to its higher potency towards AChE than BChE (IC_50_ = 0.59, 0.77 μM against AChE and BChE, respectively). Similar observations are also shown by the potent synthesized agents (SI_(BChE/AChE)_ = 1.68, 1.50 for compounds 8e and 8g, respectively). However, compound 8h shows a different behavior (SI = 1.17, 0.85 for SI_(AChE/BChE)_, SI_(BChE/AChE)_, respectively).

**Table 2:** Cholinesterase inhibition properties of the synthesized dispiro-compounds 8a–l and Donepezil

Entry	Compd.	IC_50_ of AChE (μM) ± SD	IC_50_ of BChE (μM) ± SD	SI_(AChE/BChE)_	SI_(BChE/AChE)_
1	8a	116.58 ± 12.28	81.75 ± 9.55	1.43	0.70
2	8b	13.60 ± 0.62	42.45 ± 6.26	0.32	3.12
3	8c	102.89 ± 10.13	71.75 ± 2.58	1.43	0.70
4	8d	24.30 ± 4.08	21.33 ± 2.81	1.14	0.88
5	8e	3.35 ± 0.03	5.63 ± 0.60	0.60	1.68
6	8f	12.25 ± 1.72	20.10 ± 0.16	0.61	1.64
7	8g	3.15 ± 0.63	4.74 ± 0.43	0.66	1.50
8	8h	6.27 ± 0.08	5.34 ± 0.76	1.17	0.85
9	8i	39.41 ± 2.23	35.12 ± 0.11	1.12	0.89
10	8j	20.98 ± 1.54	13.58 ± 0.37	1.54	0.65
11	8k	13.93 ± 0.18	10.22 ± 0.76	1.36	0.73
12	8l	21.97 ± 2.82	35.54 ± 0.33	0.62	1.62
13	Donepezil	0.59 ± 0.08	0.77 ± 0.01	0.77	1.31

Structure–activity relationship (SAR) based on the exhibited biological observations explain that, attachment of phenyl ring at pyrrolidinyl C-4′ seems more favorable than the *p*-chlorophenyl ring for AChE inhibitory properties (8k is an exception) as shown in pairs 8b/8d and 8h/8j (IC_50_ = 13.60, 24.30, 6.27, 20.98 μM, for 8b, 8d, 8h and 8j, respectively). Meanwhile, when *p*-methoxyphenyl ring is considered at pyrrolidinyl C-4′, better biologically active agents are optimized as shown in pairs 8b/8f and 8c/8g (IC_50_ = 13.60, 12.25, 102.89, 3.15 μM, for 8b, 8f, 8c and 8g, respectively). However, different SAR was noticed for BChE inhibitory properties. Where, the *p*-chlorophenyl ring containing compounds at pyrrolidinyl C-4′ are of higher BChE inhibitory properties than those of phenyl ring (compound 8h is an exception) as shown in pairs 8b/8d, 8c/8e and 8i/8k (IC_50_ = 42.45, 21.33, 71.75, 5.63, 35.12, 10.22 μM, for 8b, 8d, 8c, 8e, 8i and 8k, respectively). The same observation was also noticed for *p*-methoxyphenyl ring relative to phenyl ring due to BChE inhibitory properties similar to that of AChE as shown in pairs 8b/8f and 8c/8g (IC_50_ = 42.45, 20.10, 71.75 and 4.74 μM, for 8b, 8f, 8c and 8g, respectively). Molecular modeling studies can make the biological properties more understandable and identify the rules optimizing biological properties.

#### Antiproliferative properties

Antiproliferative properties of the synthesized agents 8a–l were considered against RPE1 (human immortalized retinal pigment epithelial cell line) normal cell line utilizing the standard MTT (tetrazolium salt) technique.^37^ The adopted technique is useful for exploring the cytotoxicity properties of the synthesized agents. From the obtained results (ESI Fig. S39†), it has been noticed that none of the synthesized agents reveal any cytotoxicological properties against the tested cell line (IC_50_ = >100.0 μM).

#### Acute toxicological bio-assay

The most effective agents synthesized with cholinesterase properties (8e, 8g and 8h) were subjected for acute toxicological bio-assay in mice utilizing the standard technique.^38^ None of the tested agents reveal any mortality rates or toxicological symptoms (including animal body, legs, hair or tail) at the tested doses (50, 100 and 250 mg kg^−1^ mice body weight) supporting the safe utility of the tested agents at the mentioned doses.

### Molecular modeling studies

Molecular modeling is an efficient technique useful for validating biological properties and identifying the parameters necessary for bio-properties beside its unique importance for developing/estimating novel hits/leads.

#### 2D-QSAR

The cholinesterase properties observed were undertaken by CODESSA-Pro software accessible for optimizing 2D-QSAR models.^39–41^ This is useful for well understanding the bio-observations and identifying the parameters necessary for bio-potency. Three descriptor QSAR models were developed due to the AChE and BChE inhibitory properties of the synthesized dispiro-compounds 8a–l (ESI Tables S4–S9, Fig. S40 and S41, details of the QSAR descriptors are also mentioned in the ESI).†

Goodness of the QSAR models was supported by the comparative values of the correlation coefficient (*R*^2^) with their cross validation of leave one-out (*R*^2^cvOO) and leave many-out (*R*^2^cvMO) [*R*^2^ = 0.923, 0.979; *R*^2^cvOO = 0.882, 0.936; *R*^2^cvMO = 0.904, 0.956, for AChE and BChE models, respectively]. Standard deviation (*s*^2^) and Fisher criteria (*F*) values of the models also support their goodness (*s*^2^ = 0.026, 0.005; *F* = 31.948, 122.564, for AChE and BChE models, respectively). Additionally, the correlations of the observed and predicted values “specially the high potent synthesized compounds” add good support for the attained models.

#### 3D-pharmacophore

3D-pharmacophore modelling of the synthesized agents 8a–l was utilized by Discovery Studio 2.5 software (ESI Fig. S42–S45, Tables S10 and S11†). It has been noticed that, 3D-pharmacophoric modelling of the tested compounds as AChE inhibitors comprises four chemical features (two hydrophobics “H-1, H-2”, one hydrogen bonding acceptor “HBA” and one positive ionisable “PosIon”). The tested compounds were fitted with variable affinity with the mentioned chemical features giving rise to different estimated properties. Aryl groups attached to C-4′ and N-1 are mapped with the hydrophobics H-1 and H-2, respectively. Meanwhile, the pyrrolidinyl carbonyl at C-2′′ and pyrrolidinyl N-1′ are mapped with the HBA and PosIon, respectively. Mapping of the aryl rings at H-1 and H-2 explains their necessity in optimizing the AChE inhibitory properties. This observation supports the mentioned SAR due to the experimentally obtained results.

The 3D-pharmacophore modelling of the synthesized agents as BChE inhibitors exhibits three chemical features (hydrophobic “H”, hydrogen bonding acceptor “HBA” and hydrogen bonding donor “HBD”). The aryl ring attached to the pyrrolidinyl N-1′′ is mapped with the hydrophobic “H”. Meanwhile, the pyrrolidinyl carbonyl at C-2′′ and indolyl N-1 are mapped with the HBA and HBD, receptively. Again, mapping of the aryl ring at N-1′′ supported its importance for the revealed BChE inhibitory properties.

## Conclusion

In conclusion it can be stated that, the synthesized dispiro-compounds are good cholinesterase inhibitors especially compounds 8e and 8g which show good potency and selectivity index towards AChE over BChE similar to Donepezil (clinically used cholinesterase inhibitory drug). The attained QSAR and 3D-pharmacophore models are good enough to be considered for developing novel effective hits/leads with enhanced potency/efficacy considering the elements controlling bio-observations (mainly the aryl rings attached to the pyrrolidinedione). Additionally, the multi-component azomethine cycloaddition procedure is an accessible technique for developing the targeted dispiro[indoline-3,2′-pyrrolidine-3′,3′′-pyrrolidines] in good yield (66–82%) and high regioselectivity.

## Experimental

Melting points were determined on a capillary point apparatus (Stuart SMP3) equipped with a digital thermometer. IR spectra (KBr) were recorded on a Shimadzu FT-IR 8400S spectrophotometer. Reactions were monitored using thin layer chromatography (TLC) on 0.2 mm silica gel F254 plates (Merck) utilizing various solvents for elution. The chemical structures of the synthesized compounds were characterized by nuclear magnetic resonance spectra (^1^H-NMR, ^13^C-NMR) and determined on a Bruker NMR spectrometer (500 MHz, 125 MHz for ^1^H and ^13^C, respectively). ^13^C-NMR spectra are fully decoupled. Chemical shifts were reported in parts per million (ppm) using the deuterated solvent peak or tetramethylsilane as an internal standard. Colorimetric enzyme inhibitory assays were performed in 96-well plates and the absorbance was recorded utilizing a microplate reader (Infinite F50, Tecan, Switzerland).

### Synthesis of dispiro[indoline-3,2′-pyrrolidine-3′,3′′-pyrrolidines] 8a–l (general procedure)

A mixture of equimolar amount of the appropriate 5a–e (5 mmol), isatin 6a–c and sarcosine 7 in absolute ethanol (25 mL) was boiled under reflux for the specific time. The separated solid while boiling, was collected and crystallized from a suitable solvent affording the corresponding 8a–g,i–k. In case of 8h,l the clear reaction mixture was stored at room temperature (20–25 °C) overnight. So, the separated solid was collected and purified by crystallization from a suitable solvent.

### 1′-Methyl-1′′,4′-diphenyldispiro[indoline-3,2′-pyrrolidine-3′,3′′-pyrrolidine]-2,2′′,5′′-trione (8a)

It was obtained from the reaction of 5a with 6a and sarcosine for 10 h as colorless microcrystals from *n*-butanol with mp 236–238 °C and yield 76% (1.65 g). IR: *ν*_max_/cm^−1^ 3480, 3067, 2870, 1786, 1713, 1616, 1597. ^1^H-NMR (DMSO-*d*_6_) *δ* (ppm): 2.10 (s, 3H, NCH_3_), 2.37 (d, *J* = 18.3 Hz, 1H, upfield H of pyrrolidinedionyl H_2_C-4′′), 2.71 (d, *J* = 18.3 Hz, 1H, downfield H of pyrrolidinedionyl H_2_C-4′′), 3.49 (t, *J* = 8.6 Hz, 1H, upfield H of pyrrolidinyl H_2_C-5′), 3.84 (t, *J* = 9.3 Hz, 1H, downfield H of pyrrolidinyl H_2_C-5′), 4.39 (t, *J* = 8.9 Hz, 1H, pyrrolidinyl HC-4′), 6.75 (dd, *J* = 1.8, 7.7 Hz, 2H, arom. H), 6.89 (d, *J* = 7.7 Hz, 1H, arom. H), 6.99 (t, *J* = 7.6 Hz, 1H, arom. H), 7.21 (d, *J* = 7.4 Hz, 1H, arom. H), 7.30–7.43 (m, 7H, arom. H), 7.49 (d, *J* = 7.4 Hz, 2H, arom. H), 10.78 (s, 1H, NH). ^13^C-NMR (DMSO-*d*_6_) *δ* (ppm): 34.6 (NCH_3_), 36.7 [pyrrolidinedionyl CH_2_ (C-4′′)], 48.4 [pyrrolidinyl CH (C-4′)], 58.4 [pyrrolidinyl CH_2_ (C-5′)], 61.0 [C-3′ (C-3′′)], 77.6 [C-3 (C-2′)], 110.0, 122.0, 125.0, 126.5, 126.8, 127.4, 128.4, 128.6, 128.7, 129.9, 131.6, 137.9, 142.7 (arom. C), 173.0, 177.1, 177.5 (CO). Anal. calcd for C_27_H_23_N_3_O_3_ (437.50): C, 74.13; H, 5.30; N, 9.60. Found: C, 74.24; H, 5.49; N, 9.68.

### 5-Chloro-1′-methyl-1′′,4′-diphenyldispiro[indoline-3,2′-pyrrolidine-3′,3′′-pyrrolidine]-2,2′′,5′′-trione (8b)

It was obtained from the reaction of 5a with 6b and sarcosine for 12 h as colorless microcrystals from *N*,*N*-dimethylformamide–water (2–1 v/v) with mp 253–255 °C and yield 82% (1.94 g). IR: *ν*_max_/cm^−1^ 3472, 3063, 2874, 1782, 1713, 1616, 1597. ^1^H-NMR (DMSO-*d*_6_) *δ* (ppm): 2.11 (s, 3H, NCH_3_), 2.39 (d, *J* = 18.2 Hz, 1H, upfield H of pyrrolidinedionyl H_2_C-4′′), 2.70 (d, *J* = 18.2 Hz, 1H, downfield H of pyrrolidinedionyl H_2_C-4′′), 3.51 (t, *J* = 8.6 Hz, 1H, upfield H of pyrrolidinyl H_2_C-5′), 3.80 (t, *J* = 9.3 Hz, 1H, downfield H of pyrrolidinyl H_2_C-5′), 4.37 (t, *J* = 9.0 Hz, 1H, pyrrolidinyl HC-4′), 6.82 (dd, *J* = 1.6, 8.0 Hz, 2H, arom. H), 6.91 (d, *J* = 8.3 Hz, 1H, arom. H), 7.19 (d, *J* = 2.2 Hz, 1H, arom. H), 7.33 (t, *J* = 7.3 Hz, 1H, arom. H), 7.37–7.43 (m, 6H, arom. H), 7.48 (d, *J* = 7.4 Hz, 2H, arom. H), 10.94 (s, 1H, NH). ^13^C-NMR (DMSO-*d*_6_) *δ* (ppm): 34.6 (NCH_3_), 36.5 [pyrrolidinedionyl CH_2_ (C-4′′)], 48.5 [pyrrolidinyl CH (C-4′)], 58.3 [pyrrolidinyl CH_2_ (C-5′)], 61.0 [C-3′ (C-3′′)], 77.5 [C-3 (C-2′)], 111.5, 126.2, 126.3, 126.6, 127.0, 127.4, 128.5, 128.6, 128.7, 129.9, 130.0, 131.5, 137.6, 141.7 (arom. C), 172.8, 176.7, 177.4 (CO). Anal. calcd for C_27_H_22_ClN_3_O_3_ (471.94): C, 68.72; H, 4.70; N, 8.90. Found: C, 68.96; H, 4.84; N, 9.06.

### 5-Methoxy-1′-methyl-1′′,4′-diphenyldispiro[indoline-3,2′-pyrrolidine-3′,3′′-pyrrolidine]-2,2′′,5′′-trione (8c)

It was obtained from the reaction of 5a with 6c and sarcosine for 12 h as colorless microcrystals from *n*-butanol with mp 253–255 °C and yield 73% (1.70 g). IR: *ν*_max_/cm^−1^ 3472, 3063, 2870, 1778, 1713, 1697, 1605, 1497. ^1^H-NMR (DMSO-*d*_6_) *δ* (ppm): 2.11 (s, 3H, NCH_3_), 2.36 (d, *J* = 18.2 Hz, 1H, upfield H of pyrrolidinedionyl H_2_C-4′′), 2.64 (d, *J* = 18.2 Hz, 1H, downfield H of pyrrolidinedionyl H_2_C-4′′), 3.49 (t, *J* = 8.5 Hz, 1H, upfield H of pyrrolidinyl H_2_C-5′), 3.55 (s, 3H, OCH_3_), 3.83 (t, *J* = 9.3 Hz, 1H, downfield H of pyrrolidinyl H_2_C-5′), 4.37 (t, *J* = 8.9 Hz, 1H, pyrrolidinyl HC-4′), 6.73–6.75 (m, 3H, arom. H), 6.82 (d, *J* = 8.4 Hz, 1H, arom. H), 6.90 (dd, *J* = 2.6, 8.5 Hz, 1H, arom. H), 7.31–7.42 (m, 6H, arom. H), 7.51 (d, *J* = 7.4 Hz, 2H, arom. H), 10.63 (s, 1H, NH). ^13^C-NMR (DMSO-*d*_6_) *δ* (ppm): 34.6 (NCH_3_), 36.8 [pyrrolidinedionyl CH_2_ (C-4′′)], 48.1 [pyrrolidinyl CH (C-4′)], 55.1 (OCH_3_), 58.7 [pyrrolidinyl CH_2_ (C-5′)], 61.4 [C-3′ (C-3′′)], 77.9 [C-3 (C-2′)], 110.4, 113.2, 114.6, 126.2, 126.7, 127.3, 128.4, 128.57, 128.61, 130.1, 131.6, 135.8, 138.1, 154.9 (arom. C), 172.9, 177.0, 177.6 (CO). Anal. calcd for C_28_H_25_N_3_O_4_ (467.53): C, 71.93; H, 5.39; N, 8.99. Found: C, 71.99; H, 5.60; N, 9.22.

### 5-Chloro-4′-(4-chlorophenyl)-1′-methyl-1′′-phenyldispiro[indoline-3,2′-pyrrolidine-3′,3′′-pyrrolidine]-2,2′′,5′′-trione (8d)

It was obtained from the reaction of 5b with 6b and sarcosine for 12 h as colorless microcrystals from *n*-butanol with mp 232–234 °C and yield 75% (1.89 g). IR: *ν*_max_/cm^−1^ 3487, 3059, 2874, 1790, 1713, 1620, 1597. ^1^H-NMR (DMSO-*d*_6_) *δ* (ppm): 2.11 (s, 3H, NCH_3_), 2.42 (d, *J* = 18.1 Hz, 1H, upfield H of pyrrolidinedionyl H_2_C-4′′), 2.67 (d, *J* = 18.2 Hz, 1H, downfield H of pyrrolidinedionyl H_2_C-4′′), 3.52 (t, *J* = 8.7 Hz, 1H, upfield H of pyrrolidinyl H_2_C-5′), 3.74 (t, *J* = 9.3 Hz, 1H, downfield H of pyrrolidinyl H_2_C-5′), 4.37 (t, *J* = 8.9 Hz, 1H, pyrrolidinyl HC-4′), 6.83 (dd, *J* = 1.5, 8.0 Hz, 2H, arom. H), 6.92 (d, *J* = 8.3 Hz, 1H, arom. H), 7.16 (d, *J* = 2.2 Hz, 1H, arom. H), 7.38–7.41 (m, 4H, arom. H), 7.47 (d, *J* = 8.5 Hz, 2H, arom. H), 7.53 (d, *J* = 8.5 Hz, 2H, arom. H), 10.97 (s, 1H, NH). ^13^C-NMR (DMSO-*d*_6_) *δ* (ppm): 34.6 (NCH_3_), 36.6 [pyrrolidinedionyl CH_2_ (C-4′′)], 47.7 [pyrrolidinyl CH (C-4′)], 58.7 [pyrrolidinyl CH_2_ (C-5′)], 61.0 [C-3′ (C-3′′)], 77.6 [C-3 (C-2′)], 111.6, 126.2, 126.3, 126.6, 126.9, 128.46, 128.54, 128.7, 130.0, 131.5, 132.0, 132.1, 136.8, 141.7 (arom. C), 172.8, 176.8, 177.3 (CO). Anal. calcd for C_27_H_21_Cl_2_N_3_O_3_ (506.38): C, 64.04; H, 4.18; N, 8.30. Found: C, 63.74; H, 4.35; N, 8.44.

### 4′-(4-Chlorophenyl)-5-methoxy-1′-methyl-1′′-phenyldispiro[indoline-3,2′-pyrrolidine-3′,3′′-pyrrolidine]-2,2′′,5′′-trione (8e)

It was obtained from the reaction of 5b with 6c and sarcosine for 14 h as colorless microcrystals from *n*-butanol with mp 228–230 °C and yield 80% (2.00 g). IR: *ν*_max_/cm^−1^ 3487, 3059, 2874, 1786, 1713, 1601, 1489. ^1^H-NMR (DMSO-*d*_6_) *δ* (ppm): 2.11 (s, 3H, NCH_3_), 2.39 (d, *J* = 18.1 Hz, 1H, upfield H of pyrrolidinedionyl H_2_C-4′′), 2.62 (d, *J* = 18.1 Hz, 1H, downfield H of pyrrolidinedionyl H_2_C-4′′), 3.51 (t, *J* = 8.6 Hz, 1H, upfield H of pyrrolidinyl H_2_C-5′), 3.54 (s, 3H, OCH_3_), 3.77 (t, *J* = 9.2 Hz, 1H, downfield H of pyrrolidinyl H_2_C-5′), 4.37 (t, *J* = 8.9 Hz, 1H, pyrrolidinyl HC-4′), 6.70 (d, *J* = 2.5 Hz, 1H, arom. H), 6.75 (dd, *J* = 2.0, 7.4 Hz, 2H, arom. H), 6.83 (d, *J* = 8.4 Hz, 1H, arom. H), 6.90 (dd, *J* = 2.6, 8.5 Hz, 1H, arom. H), 7.36–7.38 (m, 3H, arom. H), 7.47 (d, *J* = 8.5 Hz, 2H, arom. H), 7.55 (d, *J* = 8.4 Hz, 2H, arom. H), 10.66 (s, 1H, NH). ^13^C-NMR (DMSO-*d*_6_) *δ* (ppm): 34.5 (NCH_3_), 36.9 [pyrrolidinedionyl CH_2_ (C-4′′)], 47.3 [pyrrolidinyl CH (C-4′)], 55.1 (OCH_3_), 59.0 [pyrrolidinyl CH_2_ (C-5′)], 61.3 [C-3′ (C-3′′)], 77.9 [C-3 (C-2′)], 110.5, 113.0, 114.6, 126.1, 126.7, 128.4, 128.5, 128.53, 131.6, 132.0, 132.1, 135.8, 137.3, 155.0 (arom. C), 172.9, 177.1, 177.4 (CO). Anal. calcd for C_28_H_24_ClN_3_O_4_ (501.97): C, 67.00; H, 4.82; N, 8.37. Found: C, 67.19; H, 5.04; N, 8.42.

### 5-Chloro-4′-(4-methoxyphenyl)-1′-methyl-1′′-phenyldispiro[indoline-3,2′-pyrrolidine-3′,3′′-pyrrolidine]-2,2′′,5′′-trione (8f)

It was obtained from the reaction of 5c with 6b and sarcosine for 12 h as colorless microcrystals from *n*-butanol with mp 223–225 °C and yield 72% (1.80 g). IR: *ν*_max_/cm^−1^ 3483, 3078, 2832, 1786, 1713, 1616. ^1^H-NMR (DMSO-*d*_6_) *δ* (ppm): 2.11 (s, 3H, NCH_3_), 2.42 (d, *J* = 18.2 Hz, 1H, upfield H of pyrrolidinedionyl H_2_C-4′′), 2.68 (d, *J* = 18.2 Hz, 1H, downfield H of pyrrolidinedionyl H_2_C-4′′), 3.48 (t, *J* = 8.6 Hz, 1H, upfield H of pyrrolidinyl H_2_C-5′), 3.76 (s, 3H, OCH_3_), 3.76 (t, *J* = 9.3 Hz, 1H, downfield H of pyrrolidinyl H_2_C-5′), 4.32 (t, *J* = 9.0 Hz, 1H, pyrrolidinyl HC-4′), 6.84 (dd, *J* = 1.4, 8.0 Hz, 2H, arom. H), 6.91 (d, *J* = 8.3 Hz, 1H, arom. H), 6.97 (d, *J* = 8.7 Hz, 2H, arom. H), 7.21 (d, *J* = 2.1 Hz, 1H, arom. H), 7.37–7.42 (m, 6H, arom. H), 10.92 (s, 1H, NH). ^13^C-NMR (DMSO-*d*_6_) *δ* (ppm): 34.6 (NCH_3_), 36.4 [pyrrolidinedionyl CH_2_ (C-4′′)], 48.0 [pyrrolidinyl CH (C-4′)], 55.0 (OCH_3_), 58.4 [pyrrolidinyl CH_2_ (C-5′)], 61.0 [C-3′ (C-3′′)], 77.5 [C-3 (C-2′)], 111.5, 114.0, 126.2, 126.4, 126.6, 127.1, 128.5, 128.8, 129.3, 129.8, 131.0, 131.5, 141.7, 158.5 (arom. C), 172.9, 176.7, 177.6 (CO). Anal. calcd for C_28_H_24_ClN_3_O_4_ (501.97): C, 67.00; H, 4.82; N, 8.37. Found: C, 67.20; H, 4.66; N, 8.18.

### 5-Methoxy-4′-(4-methoxyphenyl)-1′-methyl-1′′-phenyldispiro[indoline-3,2′-pyrrolidine-3′,3′′-pyrrolidine]-2,2′′,5′′-trione (8g)

It was obtained from the reaction of 5c with 6c and sarcosine for 15 h as colorless microcrystals from *n*-butanol with mp 217–219 °C and yield 68% (1.68 g). IR: *ν*_max_/cm^−1^ 3480, 3075, 2959, 1782, 1713, 1601. ^1^H-NMR (DMSO-*d*_6_) *δ* (ppm): 2.11 (s, 3H, NCH_3_), 2.38 (d, *J* = 18.3 Hz, 1H, upfield H of pyrrolidinedionyl H_2_C-4′′), 2.62 (d, *J* = 18.2 Hz, 1H, downfield H of pyrrolidinedionyl H_2_C-4′′), 3.46 (t, *J* = 8.5 Hz, 1H, upfield H of pyrrolidinyl H_2_C-5′), 3.55 (s, 3H, OCH_3_), 3.76 (s, 3H, OCH_3_), 3.79 (t, *J* = 9.2 Hz, 1H, downfield H of pyrrolidinyl H_2_C-5′), 4.32 (t, *J* = 8.9 Hz, 1H, pyrrolidinyl HC-4′), 6.74–6.76 (m, 3H, arom. H), 6.81 (d, *J* = 8.4 Hz, 1H, arom. H), 6.89 (dd, *J* = 2.6, 8.5 Hz, 1H, arom. H), 6.97 (d, *J* = 8.8 Hz, 2H, arom. H), 7.37–7.43 (m, 5H, arom. H), 10.61 (s, 1H, NH). ^13^C-NMR (DMSO-*d*_6_) *δ* (ppm): 34.6 (NCH_3_), 36.7 [pyrrolidinedionyl CH_2_ (C-4′′)], 47.6 [pyrrolidinyl CH (C-4′)], 55.0 (OCH_3_), 55.1 (OCH_3_), 58.8 [pyrrolidinyl CH_2_ (C-5′)], 61.4 [C-3′ (C-3′′)], 77.9 [C-3 (C-2′)], 110.4, 113.2, 114.0, 114.5, 126.3, 126.7, 128.4, 128.6, 129.8, 131.1, 131.6, 135.8, 154.9, 158.4 (arom. C), 173.0, 177.1, 177.7 (CO). Anal. calcd for C_29_H_27_N_3_O_5_ (497.55): C, 70.01; H, 5.47; N, 8.45. Found: C, 69.87; H, 5.40; N, 8.25.

### 1′′-(4-Chlorophenyl)-1′-methyl-4′-phenyldispiro[indoline-3,2′-pyrrolidine-3′,3′′-pyrrolidine]-2,2′′,5′′-trione (8h)

It was obtained from the reaction of 5d with 6a and sarcosine for 10 h as pale yellow microcrystals from *n*-butanol with mp 208–210 °C (lit. 189–190 °C (ref. 33)) and yield 81% (1.90 g). IR: *ν*_max_/cm^−1^ 3480, 3067, 2951, 1778, 1713, 1616, 1597. ^1^H-NMR (DMSO-*d*_6_) *δ* (ppm): 2.09 (s, 3H, NCH_3_), 2.39 (d, *J* = 18.2 Hz, 1H, upfield H of pyrrolidinedionyl H_2_C-4′′), 2.67 (d, *J* = 18.2 Hz, 1H, downfield H of pyrrolidinedionyl H_2_C-4′′), 3.49 (t, *J* = 8.6 Hz, 1H, upfield H of pyrrolidinyl H_2_C-5′), 3.82 (t, *J* = 9.3 Hz, 1H, downfield H of pyrrolidinyl H_2_C-5′), 4.37 (t, *J* = 8.9 Hz, 1H, pyrrolidinyl HC-4′), 6.79 (dd, *J* = 2.0, 6.8 Hz, 2H, arom. H), 6.88 (d, *J* = 7.7 Hz, 1H, arom. H), 6.97 (t, *J* = 7.6 Hz, 1H, arom. H), 7.16 (d, *J* = 7.4 Hz, 1H, arom. H), 7.28–7.33 (m, 2H, arom. H), 7.39–7.50 (m, 6H, arom. H), 10.78 (s, 1H, NH). ^13^C-NMR (DMSO-*d*_6_) *δ* (ppm): 34.5 (NCH_3_), 36.9 [pyrrolidinedionyl CH_2_ (C-4′′)], 48.2 [pyrrolidinyl CH (C-4′)], 58.6 [pyrrolidinyl CH_2_ (C-5′)], 61.2 [C-3′ (C-3′′)], 77.6 [C-3 (C-2′)], 110.0, 122.0, 125.0, 126.2, 127.3, 128.5, 128.6, 128.7, 129.99, 130.0, 130.3, 132.9, 138.0, 142.7 (arom. C), 172.7, 177.1, 177.3 (CO). Anal. calcd for C_27_H_22_ClN_3_O_3_ (471.94): C, 68.72; H, 4.70; N, 8.90. Found: C, 68.95; H, 4.86; N, 8.81.

### 5-Chloro-1′′-(4-chlorophenyl)-1′-methyl-4′-phenyldispiro[indoline-3,2′-pyrrolidine-3′,3′′-pyrrolidine]-2,2′′,5′′-trione (8i)

It was obtained from the reaction of 5d with 6b and sarcosine for 10 h as colorless microcrystals from *n*-butanol with mp 240–242 °C and yield 87% (2.20 g). IR: *ν*_max_/cm^−1^ 3476, 3102, 2839, 1778, 1713, 1620. ^1^H-NMR (DMSO-*d*_6_) *δ* (ppm): 2.12 (s, 3H, NCH_3_), 2.42 (d, *J* = 18.2 Hz, 1H, upfield H of pyrrolidinedionyl H_2_C-4′′), 2.70 (d, *J* = 18.2 Hz, 1H, downfield H of pyrrolidinedionyl H_2_C-4′′), 3.51 (t, *J* = 8.6 Hz, 1H, upfield H of pyrrolidinyl H_2_C-5′), 3.81 (t, *J* = 9.3 Hz, 1H, downfield H of pyrrolidinyl H_2_C-5′), 4.37 (t, *J* = 8.9 Hz, 1H, pyrrolidinyl HC-4′), 6.88 (d, *J* = 8.7 Hz, 2H, arom. H), 6.92 (d, *J* = 8.3 Hz, 1H, arom. H), 7.17 (d, *J* = 2.2 Hz, 1H, arom. H), 7.31–7.42 (m, 4H, arom. H), 7.48–7.50 (m, 4H, arom. H), 10.95 (s, 1H, NH). ^13^C-NMR (DMSO-*d*_6_) *δ* (ppm): 34.6 (NCH_3_), 36.6 [pyrrolidinedionyl CH_2_ (C-4′′)], 48.5 [pyrrolidinyl CH (C-4′)], 58.5 [pyrrolidinyl CH_2_ (C-5′)], 61.1 [C-3′ (C-3′′)], 77.5 [C-3 (C-2′)], 111.6, 126.22, 126.24, 127.0, 127.4, 128.3, 128.6, 128.8, 129.9, 130.1, 130.3, 133.0, 137.6, 141.7 (arom. C), 172.6, 176.7, 177.2 (CO). Anal. calcd for C_27_H_21_Cl_2_N_3_O_3_ (506.38): C, 64.04; H, 4.18; N, 8.30. Found: C, 64.21; H, 4.26; N, 8.41.

### 1′′,4′-Bis(4-chlorophenyl)-1′-methyldispiro[indoline-3,2′-pyrrolidine-3′,3′′-pyrrolidine]-2,2′′,5′′-trione (8j)

It was obtained from the reaction of 5e with 6a and sarcosine for 10 h as colorless microcrystals from *n*-butanol with mp 225–227 °C (lit. 224–226 °C (ref. 33)) and yield 81% (2.05 g). IR: *ν*_max_/cm^−1^ 3487, 3063, 2878, 1790, 1717, 1620, 1597, 1493. ^1^H-NMR (DMSO-*d*_6_) *δ* (ppm): 2.08 (s, 3H, NCH_3_), 2.41 (d, *J* = 18.1 Hz, 1H, upfield H of pyrrolidinedionyl H_2_C-4′′), 2.64 (d, *J* = 18.1 Hz, 1H, downfield H of pyrrolidinedionyl H_2_C-4′′), 3.51 (t, *J* = 8.6 Hz, 1H, upfield H of pyrrolidinyl H_2_C-5′), 3.75 (t, *J* = 9.2 Hz, 1H, downfield H of pyrrolidinyl H_2_C-5′), 4.36 (t, *J* = 8.9 Hz, 1H, pyrrolidinyl HC-4′), 6.79 (dd, *J* = 1.9, 6.8 Hz, 2H, arom. H), 6.89 (d, *J* = 7.7 Hz, 1H, arom. H), 6.97 (t, *J* = 7.7 Hz, 1H, arom. H), 7.13 (d, *J* = 7.4 Hz, 1H, arom. H), 7.30 (dt, *J* = 1.0, 7.7 Hz, 1H, arom. H), 7.44–7.47 (m, 4H, arom. H), 7.53 (d, *J* = 8.5 Hz, 2H, arom. H), 10.80 (s, 1H, NH). ^13^C-NMR (DMSO-*d*_6_) *δ* (ppm): 34.5 (NCH_3_), 36.9 [pyrrolidinedionyl CH_2_ (C-4′′)], 47.4 [pyrrolidinyl CH (C-4′)], 58.9 [pyrrolidinyl CH_2_ (C-5′)], 61.1 [C-3′ (C-3′′)], 77.6 [C-3 (C-2′)], 110.1, 122.1, 124.8, 126.1, 128.5, 128.7, 130.1, 130.3, 132.0, 132.1, 132.9, 137.2, 142.7 (arom. C), 172.7, 177.2 (CO). Anal. calcd for C_27_H_21_Cl_2_N_3_O_3_ (506.38): C, 64.04; H, 4.18; N, 8.30. Found: C, 63.88; H, 3.97; N, 8.06.

### 5-Chloro-1′′,4′-bis(4-chlorophenyl)-1′-methyldispiro[indoline-3,2′-pyrrolidine-3′,3′′-pyrrolidine]-2,2′′,5′′-trione (8k)

It was obtained from the reaction of 5e with 6b and sarcosine for 9 h as colorless microcrystals from *n*-butanol with mp 236–238 °C and yield 66% (1.77 g). IR: *ν*_max_/cm^−1^ 3487, 3105, 2874, 1786, 1713, 1620, 1493. ^1^H-NMR (DMSO-*d*_6_) *δ* (ppm): 2.10 (s, 3H, NCH_3_), 2.42 (d, *J* = 18.1 Hz, 1H, upfield H of pyrrolidinedionyl H_2_C-4′′), 2.65 (d, *J* = 18.0 Hz, 1H, downfield H of pyrrolidinedionyl H_2_C-4′′), 3.52 (t, *J* = 8.7 Hz, 1H, upfield H of pyrrolidinyl H_2_C-5′), 3.72 (t, *J* = 9.3 Hz, 1H, downfield H of pyrrolidinyl H_2_C-5′), 4.35 (t, *J* = 8.9 Hz, 1H, pyrrolidinyl HC-4′), 6.87 (dd, *J* = 2.0, 6.8 Hz, 2H, arom. H), 6.91 (d, *J* = 8.3 Hz, 1H, arom. H), 7.11 (d, *J* = 2.2 Hz, 1H, arom. H), 7.37 (dd, *J* = 2.2, 8.3 Hz, 1H, arom. H), 7.47–7.54 (m, 6H, arom. H), 10.97 (s, 1H, NH). ^13^C-NMR (DMSO-*d*_6_) *δ* (ppm): 34.6 (NCH_3_), 36.7 [pyrrolidinedionyl CH_2_ (C-4′′)], 47.5 [pyrrolidinyl CH (C-4′)], 58.8 [pyrrolidinyl CH_2_ (C-5′)], 61.1 [C-3′ (C-3′′)], 77.5 [C-3 (C-2′)], 111.6, 126.0, 126.3, 126.9, 128.2, 128.5, 128.8, 130.0, 130.3, 132.06, 132.1, 132.9, 136.8, 141.7 (arom. C), 172.5, 176.7, 177.1 (CO). Anal. calcd for C_27_H_20_Cl_3_N_3_O_3_ (540.83): C, 59.96; H, 3.73; N, 7.77. Found: C, 60.10; H, 3.84; N, 7.64.

### 1′′,4′-Bis(4-chlorophenyl)-5-methoxy-1′-methyldispiro[indoline-3,2′-pyrrolidine-3′,3′′-pyrrolidine]-2,2′′,5′′-trione (8l)

It was obtained from the reaction of 5e with 6c and sarcosine for 12 h as colorless microcrystals from *n*-butanol with mp 247–249 °C and yield 69% (1.84 g). IR: *ν*_max_/cm^−1^ 3476, 3210, 2870, 1778, 1697, 1632, 1605. ^1^H-NMR (DMSO-*d*_6_) *δ* (ppm): 2.11 (s, 3H, NCH_3_), 2.41 (d, *J* = 18.0 Hz, 1H, upfield H of pyrrolidinedionyl H_2_C-4′′), 2.62 (d, *J* = 18.0 Hz, 1H, downfield H of pyrrolidinedionyl H_2_C-4′′), 3.51 (t, *J* = 8.6 Hz, 1H, upfield H of pyrrolidinyl H_2_C-5′), 3.55 (s, 3H, OCH_3_), 3.77 (t, *J* = 9.2 Hz, 1H, downfield H of pyrrolidinyl H_2_C-5′), 4.38 (t, *J* = 8.9 Hz, 1H, pyrrolidinyl HC-4′), 6.68 (d, *J* = 2.5 Hz, 1H, arom. H), 6.78–6.91 (m, 4H, arom. H), 7.45–7.48 (m, 3H, arom. H), 7.56 (d, *J* = 8.5 Hz, 2H, arom. H), 10.67 (s, 1H, NH). ^13^C-NMR (DMSO-*d*_6_) *δ* (ppm): 34.5 (NCH_3_), 37.0 [pyrrolidinedionyl CH_2_ (C-4′′)], 47.2 [pyrrolidinyl CH (C-4′)], 55.1 (OCH_3_), 59.2 [pyrrolidinyl CH_2_ (C-5′)], 61.5 [C-3' (C-3′′)], 77.9 [C-3 (C-2′)], 110.5, 112.9, 114.6, 126.1, 128.4, 128.5, 128.6, 130.4, 132.0, 132.2, 132.9, 135.7, 137.3, 155.0 (arom. C), 172.7, 177.1 (CO). Anal. calcd for C_28_H_23_Cl_2_N_3_O_4_ (536.41): C, 62.70; H, 4.32; N, 7.83. Found: C, 62.91; H, 4.14; N, 8.00.

### X-ray studies

The experimental procedure was mentioned in the ESI.†

### Biological studies

All the biological procedures utilized obey the standards and approved by the Research Ethics Committee, National Research Centre, Egypt (associated with project ID: 12060101). All the experiments were performed following the relevant guidelines and regulations.

#### Chloinesterase inhibitory activity studies

The assays were undertaken by the standard technique.^42^ Briefly, 170 μL of Tris–HCl buffer (200 mM, pH 7.5) was added followed by 20 μL at different concentrations of tested compounds (125–0.977 μg mL^−1^) and then 20 μL of the enzyme solution (0.1 U mL^−1^). After incubation period of 10 min at 25 °C, 40 μL of DTNB (dithio-bis-(2-nitrobenzoic acid)) and then 20 μL of the substrate (1.11 mM) were added. Butyrylthiocholine iodide and acetylthiocholine were utilized as substrates in BChE and AChE assays, respectively, where DTNB was served as indicator. All compounds were dissolved in MeOH. The intensity of the developed color was measured at 405 nm using a microplate reader (reading A) and control without the inhibitor were measured (reading B). Blank assays were performed by replacing the enzyme (20 μL) with buffer and their absorbances were recorded for correction of the spontaneous lysis of the indicator or inherent color of the inhibitor. Linear regression was performed for calculation of the IC_50_ (50% inhibitory concentration). Microsoft EXCEL 2010 program and graph pad instate 6.0 software were used for the data analysis.% Inhibition = [1 − (corrected A/corrected B)] × 100

Selectivity index (SI) for acetyl and butyryl cholinesterases was calculated as follow:SI_(AChE/BChE)_ = IC_50_ (AChE)/IC_50_ (BChE)SI_(BChE/AChE)_ = IC_50_ (AChE)/IC_50_ (BChE)

#### Antiproliferative properties

The synthesized agents 8a–l were screened against RPE1 (human immortalized retinal pigment epithelial cell line) normal cell line to investigate their cytotoxicity by the standard mitochondrial dependent reduction of yellow MTT [3-(4,5-dimethylthiazol-2-yl)-2,5-diphenyl-tetrazolium bromide] to purple formazan technique.^37^ Cells were suspended in DMEM in addition to 1% antibiotic–antimycotic mixture (10 000 μg mL^−1^ potassium penicillin, 10 000 μg mL^−1^ streptomycin sulfate and 25 μg mL^−1^ amphotericin B), 10% fetal bovine serum and 1% l-glutamine at 37 °C, under 5% CO_2_ and 95% humidity. Cells were seeded at concentration of 30 000 cells per well in fresh complete growth medium in 96-well tissue culture microtiter plates for 24 h. Media was aspirated, fresh complete medium was added and cells were incubated with different concentrations of the tested compound to give a final concentration of (100, 50, 25 and 12.5 μM). 0.5% DMSO was used as negative control. Triplicate wells were prepared for each individual dose. After 72 h of incubation, medium was aspirated, 40 μl MTT salt (2.5 mg mL^−1^) were added to each well and incubated for further 4 h at 37 °C. To stop the reaction and dissolve the formed crystals, 150 μL of 10% sodium dodecyl sulfate (SDS) in deionized water were added to each well and incubated overnight at 37 °C. The absorbance was then measured at 570 nm and a reference wavelength of 595 nm.

Data were collected as mean values for experiments performed in triplicates for each individual dose which had been measured by MTT assay. Control experiments did not exhibit significant change compared to the DMSO vehicle. The percentage of cell survival was calculated according to the following equation.
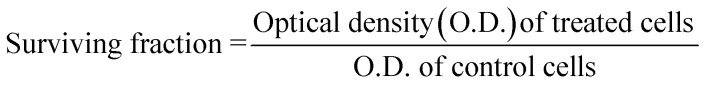


The IC_50_ (concentration required to produce 50% inhibition of cell growth compared to the control experiment) can was determined using Graph-Pad PRISM version-5 software. Statistical calculations for determination of the mean and standard error values were determined by SPSS 16 software. The observed anti-proliferative properties are presented ESI Fig. S39.†

#### Acute toxicological bio-assay

The most effective agents synthesized with cholinesterase properties (8e, 8g and 8h) were subjected for acute toxicological bio-assay in mice utilizing the standard technique.^38^ Albino mice weighing 20–25 g were divided into 4 groups of 6 mice each. Administrations of the tested compounds dissolved in saline solution (0.9%) by the aid of few drops of Tween 80 were given intraperitoneally in 50, 100 and 250 mg kg^−1^ (mice body weight). The control group was given a saline solution only with few drops of Tween 80. The toxic symptoms and mortality rates were recorded 24 h post-administration in each group.

### Molecular modeling studies

The experimental procedures in details were mentioned in the ESI.†

## Conflicts of interest

There is no conflict to declare.

## Supplementary Material

RA-010-D0RA03064C-s001

RA-010-D0RA03064C-s002
