# Supplementary material for: Synthesis and molecular modeling studies of cholinesterase inhibitor dispiro[indoline-3,2′-pyrrolidine-3′,3′′-pyrrolidines]
Source: RSC Adv. 2020 Jun 8;10(37):21830–8. doi: 10.1039/d0ra03064c (PMC9054546; doi:10.1039/d0ra03064c)
Supplement: RA-010-D0RA03064C-s001 [file RA-010-D0RA03064C-s001.pdf]

Electronic Supplementary Material (ESI) for RSC Advances.
This journal is © The Royal Society of Chemistry 2020

Synthesis and molecular modeling studies of cholinesterase inhibitor dispiro[indoline-3,2'-pyrrolidine-3',3''-pyrrolidines]

M. Adel Youssef,[a] Siva S. Panda,[b] Riham A. El-Shiekh,[c] ElSayed M. Shalaby,[d] Dalia R. Aboshouk,[e] Walid Fayad,[f] Nehmedo G. Fawzy[e] and Adel S. Girgis*[e]

[a] *Department of Chemistry, Faculty of Science, Helwan University, Helwan, Egypt*

[b] *Department of Chemistry and Physics, Augusta University, Augusta, GA 30912, USA*

[c] *Department of Pharmacognosy, Faculty of Pharmacy, Cairo University, Cairo 11562, Egypt*

[d] *X-Ray Crystallography Lab., Physics Division, National Research Centre, Dokki, Giza 12622, Egypt*

[e] *Department of Pesticide Chemistry, National Research Centre, Dokki, Giza 12622, Egypt*

[f] *Drug Bioassay-Cell Culture Laboratory, Pharmacognosy Department, National Research Centre, Dokki, Giza, 12622, Egypt*

*E-mail: girgisas10@yahoo.com

Supplementary material

Table titles

Table S1. Crystal data and structure refinement parameters for compound **8c.**

Table S2. Bond lengths (Å) of compound **8c**.

Table S3. Bond angles (°) for compound **8c**.

Table S4. Descriptor of the BMLR-QSAR model for the tested compounds as AChE inhibitors.

Table S5. Observed and estimated activity values for the tested compounds as AChE inhibitors according to the BMLR-QSAR model.

Table S6. Molecular descriptor values of the BMLR-QSAR model for the synthesized compounds as AChE inhibitors.

Table S7. Descriptor of the BMLR-QSAR model for the tested compounds as BChE inhibitors.

Table S8. Observed and estimated activity values for the tested compounds as BChE inhibitors according to the BMLR-QSAR model.

Table S9. Molecular descriptor values of the BMLR-QSAR model for the synthesized compounds as BChE inhibitors.

Table S10. Observed and estimated activity values for the tested compounds **8a–l** as AChE inhibitors according to the 3D-pharmacophore model.

Table S11. Observed and estimated activity values for the tested compounds **8a–l** as BChE inhibitors according to the 3D-pharmacophore model.

Figure captions

Fig. S1. IR spectrum of compound **8a** (KBr pellet).

Fig. S2. ^{1}H-NMR spectrum of compound **8a** in DMSO-d_6.

Fig. S3. ^{13}C-NMR spectrum of compound **8a** in DMSO-d_6.

Fig. S4. IR spectrum of compound **8b** (KBr pellet).

Fig. S5. ^{1}H-NMR spectrum of compound **8b** in DMSO-d_6.

Fig. S6. ^{13}C-NMR spectrum of compound **8b** in DMSO-d_6.

Fig. S7. HSQC spectrum of compound **8b** in DMSO-d_6.

Fig. S8. IR spectrum of compound **8c** (KBr pellet).

Fig. S9. ^{1}H-NMR spectrum of compound **8c** in DMSO-d_6.

Fig. S10. ^{13}C-NMR spectrum of compound **8c** in DMSO-d_6.

Fig. S11. IR spectrum of compound **8d** (KBr pellet).

Fig. S12. ^{1}H-NMR spectrum of compound **8d** in DMSO-d_6.

Fig. S13. ^{13}C-NMR spectrum of compound **8d** in DMSO-d_6.

Fig. S14. IR spectrum of compound **8e** (KBr pellet).

Fig. S15. ^{1}H-NMR spectrum of compound **8e** in DMSO-d_6.

Fig. S16. ^{13}C-NMR spectrum of compound **8e** in DMSO-d_6.

Fig. S17. IR spectrum of compound **8f** (KBr pellet).

Fig. S18. ^{1}H-NMR spectrum of compound **8f** in DMSO-d_6.

Fig. S19. ^{13}C-NMR spectrum of compound **8f** in DMSO-d_6.

Fig. S20. IR spectrum of compound **8g** (KBr pellet).

Fig. S21. ^{1}H-NMR spectrum of compound **8g** in DMSO-d_6.

Fig. S22. ^{13}C-NMR spectrum of compound **8g** in DMSO-d_6.

Fig. S23. HSQC spectrum of compound **8g** in DMSO-d_6.

Fig. S24. IR spectrum of compound **8h** (KBr pellet).

Fig. S25. ^{1}H-NMR spectrum of compound **8h** in DMSO-d_6.

Fig. S26. ^{13}C-NMR spectrum of compound **8h** in DMSO-d_6.

Fig. S27. IR spectrum of compound **8i** (KBr pellet).

Fig. S28. ^{1}H-NMR spectrum of compound **8i** in DMSO-d_6.

Fig. S29. ^{13}C-NMR spectrum of compound **8i** in DMSO-d_6.

Fig. S30. IR spectrum of compound **8j** (KBr pellet).

Fig. S31. ^{1}H-NMR spectrum of compound **8j** in DMSO-d_6.

Fig. S32. ^{13}C-NMR spectrum of compound **8j** in DMSO-d_6.

Fig. S33. IR spectrum of compound **8k** (KBr pellet).

Fig. S34. ^{1}H-NMR spectrum of compound **8k** in DMSO-d_6.

Fig. S35. ^{13}C-NMR spectrum of compound **8k** in DMSO-d_6.

Fig. S36. IR spectrum of compound **8l** (KBr pellet).

Fig. S37. ^{1}H-NMR spectrum of compound **8l** in DMSO-d_6.

Fig. S38. ^{13}C-NMR spectrum of compound **8l** in DMSO-d_6.

Fig. S39. Dose-response curve for the tested compounds against RPE1 (retinal pigment epithelium) cell line.

Fig. S40. BMLR-QSAR model plot of correlations representing the observed *vs.* predicted log/(IC_{50}, μM) values for the tested compounds as AChE inhibitor (compound **8d** is an outlier).

Fig. S41. BMLR-QSAR model plot of correlations representing the observed *vs.* predicted log/(IC_{50}, μM) values for the tested compounds as BChE inhibitor.

Fig. S42. **(A)** Constraint distances "H-1 – H-2 = 6.872, H-1 – PosIon = 5.599, H-1 – HBA = 5.148, H-2 – HBA = 4.747, H-2 – PosIon = 8.062, HBA – PosIon = 3.514 Å"; (B) Constraint angles "H-1 – H-2 – PosIon = 43.12, H-2 – H-1 – HBA = 43.65, HBA – H-1 – PosIon = 37.87 °" of the generated 3D-pharmacophore for the tested compounds

8a–l as AChE inhibitor which contains two hydrophobics (H-1, H-2; light blue), one hydrogen bonding acceptor (HBA; green) and one positive ionizable (PosIon; red).

Fig. S43. 3D-pharmacophore model mapped on the tested compounds **8a–l** as AChE inhibitor.

Fig. S44. (A) Constraint distances "H – HBA = 4.695, H – HBD = 7.860, HBA – HBD = 3.326 Å"; (B) Constraint angle "H – HBD – HBA = 13.64 ^{o}" of the generated 3D-pharmacophore for the tested compounds **8a–l** as BChE inhibitor which contains one hydrophobic (H; light blue), one hydrogen bonding acceptor (HBA; green) and one hydrogen bonding donor (HBD; purple).

Fig. S45. 3D-pharmacophore model mapped on the tested compounds **8a–l** as BChE inhibitor.

Single crystal X-ray studies

Suitable colorless single crystals of compound **8c** were selected for X-ray diffraction analysis. The X-ray diffraction data were collected at room temperature (298 K) on an Enraf-Nonius 590 diffractometer with a Kappa CCD detector using graphite monochromated Mo-*Kα* (λ = 0.71073Å) radiation.[1] Reflection data has been recorded in the rotation mode using the ϕ and ω scan technique with $2\theta_{max}$ = 27.912. In absence of significant anomalous scattering, Friedel pairs have been merged. Changes in illuminated volume were kept to a minimum, and were taken into account by the multi-scan inter-frame scaling .[2,3] Unit cell parameters were determined from least-squares refinement with θ in the range $3 \leq \theta \leq 27$. The structure was solved using *SUPERFLIP*[4] implemented in *CRYSTALS* program suit.[5] The refinement was carried out by full-matrix least-squares method on the positional and anisotropic temperature parameters of all non-hydrogen atoms based on F^2 using *CRYSTALS* package. All hydrogen atoms were positioned geometrically and were initially refined with soft restraints on the bond lengths and angles to regularize their geometry (C—H in the range 0.93–0.98 and N—H in the range 0.86–0.89) and U_{iso}(H) (in the range 1.2-1.5 times U_{eq} of the parent atom). Then, the positions were refined with riding constraints.[6] The general-purpose crystallographic tool *PLATON* [7] was used for the structure analysis and presentation of the results. *ORTEP-3* for Windows[8] and *MERCURY*[9] programs were used for molecular graphics. Details of the data collection conditions and the parameters of the refinement process for compound **8c** are given in Table S1.

Molecular modeling studies

The synthesized dispiro[indoline-3,2'-pyrrolidine-3',3''-pyrrolidines] **8a–l** revealing variable AChE and BChE inhibitory properties were utilized for developing the 2D-QSAR modeling by CODESSA-Pro (comprehensive descriptors for structural and statistical analysis) software. Geometry of the compounds was initially optimized by AM1 technique using hyperChem 8.0 then, uploaded to CODESSA-Pro for final geometrical structure optimization by MOPAC.[10–12] CODESSA-Pro calculated 656 molecular descriptors (constitutional, topological, geometrical, charge-related, semi-empirical, thermodynamical, molecular-type, atomic-type and bond-type descriptors) for

the exported bio-active agents. Mathematical transformation of the experimental values [including IC_{50}, $1/IC_{50}$, $\log(IC_{50})$ and $1/\log(IC_{50})$ μM] were used searching for the best QSAR model. The best multi-linear regression (BMLR) technique was utilized which is a stepwise search for the best *n*-parameter regression equations (where *n* stands for the number of descriptors used), based on the highest R^2 (squared correlation coefficient), R^2cvOO (squared cross-validation "leave one-out, LOO" coefficient), R^2cvMO (squared cross-validation "leave many-out up to 20% of the training set, LMO" coefficient), *F* (Fisher statistical significance criteria) values, and s^2 (standard deviation). The QSAR up to 3-descriptor model describing the biological activity of the bio-active agents were generated (obeying the thumb rule of 4:1 which is the ratio between the data points and the number of QSAR descriptor).

Minimum (>0.1) bond order for atom C is an atomic type descriptor with the highest coefficient value (466.922) among all the descriptors of AChE model. This is why the higher descriptor value, the lower AChE inhibitory efficacy of the tested agent as shown in compounds **8a** and **8e** (descriptor value = 0.10497, 0.10232 with estimated IC_{50} value = 129.99, 3.25 μM for compounds **8a** and **8e**, respectively). HA dependent HDCA-2/SQRT(TMSA) (MOPAC PC) is a charge related descriptor with relatively high coefficient value (85.5021). Compounds with high descriptor value predicted low AChE property and vice versa as shown for compounds **8a** and **8e** (descriptor value = 0.1247, 0.11715). HDCA2 (area weighed surface charge of hydrogen bonding donor atoms) can be calculated by equ. (1).[13]

$$HDCA2 = \sum_{D} \frac{q_D \sqrt{S_D}}{\sqrt{S_{tot}}} \quad D \in H_{H-donor} \;\ldots\ldots\ldots\ldots (1)$$

Where, S_D stands for the solvent accessible surface area of H-bonding donor H atoms (selected by threshold charge), q_D is the partial charge on H-bonding donor H atoms (selected by threshold charge) and S_{tot} is the total solvent accessible molecular surface area.

Square root of surface area for atom C is also a charge related descriptor with negative sign coefficient value (-0.164864). For this reason, the higher descriptor value of an agent, the higher predicted AChE inhibitory activity and vice versa as revealed by

compounds **8c** and **8e** (descriptor value = 55.9192, 57.81774 corresponding to predicted IC_{50} = 84.39, 3.25 μM for compounds **8c** and **8e**, respectively). Atomic charge weighed partial positively and negatively charged surface area can be calculated by equs. (2) and (3), respectively.[13]

$$PPSA3 = \sum_{A} q_A . S_A \quad A \in \{\delta_A > 0\} \text{ .... (2)}$$

$$PNSA3 = \sum_{A} q_A . S_A \quad A \in \{\delta_A > 0\} \text{ .... (3)}$$

Where, S_A is the positively/negatively charged solvent accessible atomic surface area and q_A is the atomic partial charge.

ZX Shadow/ZX rectangle is a geometrical descriptor with the highest coefficient value (10.5556) among all the 3 descriptor BChE model. The higher descriptor value of an agent the lower potency of the molecule as shown by compounds **8c** and **8e** (descriptor value = 0.68051, 0.59553 corresponding to estimated properties = 64.31, 4.77 for compounds **8c** and **8e**, respectively). Relative shadow areas of a molecule can be determined by equ. (4).[13]

$$S_k^r = \frac{\int_{(C)} (\nu d\rho - \rho d\nu)}{S^{(k)}} \text{ .... (4)}$$

Where, *C* is contour of the projection of the molecule on the plane defined by two principal axes of the molecule (*k = XY, XZ* or *YZ* plane), $\nu - x \text{ or } y$, $\rho - y \text{ or } z$, $S^{(k)} = X \bullet Y; X \bullet Z \text{ or } Y \bullet Z.$

Maximum atomic state energy for atom O is an atomic type descriptor. It is the second highest coefficient value of the model (2.22981). Again the higher descriptor value of an agent the lower potency of the molecule as shown by compounds **8a** and **8e** (descriptor value = 308.443, 308.2474 corresponding to estimated properties = 68.95, 4.77 for compounds **8a** and **8e**, respectively). Shadow plane XY is a molecular type descriptor with lowest coefficient value of the model (0.0220702). Its effect is the same similar to the previously mentioned BChE descriptor model for the potency of the

molecule as shown by compounds **8c** and **8e** (descriptor value = 107.2, 96.96 for compounds **8c** and **8e**, respectively).

References

1 R. W. W. Hooft, Collect: Data collection software, Nonius BV, Delft, The Netherlands, 1998.

2 C. Görbitz, What is the best crystal size for collection of X-ray data? Refinement of the structure of glycyl-L-serine based on data from a very large crystal, *Acta Crystallogr. B.*, 1999, **55**, 1090–1098.

3 Z. Otwinowski, W. Minor, Processing of X-ray Diffraction Data Collected in Oscillation Mode, in Methods in Enzymology, vol. 27, C.W. Carter, Jr. Sweet, Eds., New York Academic Press, 1997.

4 L. Palatinus and G. Chapuis, SUPERFLIP – a computer program for the solution of crystal structures by charge flipping in arbitrary dimensions, *J. Appl. Cryst.*, 2007, **40**, 786–790.

5 P. W. Betteridge, J. R. Carruthers, R. I. Cooper, K. Prout and D. J. Watkin, CRYSTALS version 12: software for guided crystal structure analysis, *J. Appl. Crystallogr.*, 2003, **36**, 1487.

6 R. I. Cooper, A. L. Thompson and D. J. Watkin, CRYSTALS enhancements: dealing with hydrogen atoms in refinement, *J. Appl. Crystallogr.*, 2010, **43**, 1100–1107.

7 A. L. Spek, Structure validation in chemical crystallography, *Acta Crystallogr. Sect. D Biol. Crystallogr.*, 2009, **65**, 148–155.

8 L. J. Farrugia, ORTEP -3 for Windows - a version of ORTEP -III with a Graphical User Interface (GUI), *J. Appl. Crystallogr.*, 1997, **30**, 565–565.

9 C. F. Macrae, I. J. Bruno, J. A. Chisholm, P. R. Edgington, P. McCabe, E. Pidcock, L. Rodriguez-Monge, R. Taylor, J. van de Streek and P. A. Wood, Mercury CSD 2.0 – new features for the visualization and investigation of crystal structures, *J. Appl. Crystallogr.*, 2008, **41**, 466–470.

10 I. A. Seliem, S. S. Panda, A. S. Girgis, Y. I. Nagy, R. F. George, W. Fayad, N. G. Fawzy, T. S. Ibrahim, A. M. M. Al-Mahmoudy, R. Sakhuja and Z. K. M. Abdel-

samii, Design, synthesis, antimicrobial and DNA gyrase inhibitory properties of fluoroquinolone-dichloroacetic acid hybrids, *Chem. Biol. Drug Des.*, 2020, **95**, 248–259.

11 N. G. Fawzy, S. S. Panda, W. Fayad, E. M. Shalaby, A. M. Srour and A. S. Girgis, Synthesis, human topoisomerase IIα inhibitory properties and molecular modeling studies of anti-proliferative curcumin mimics, *RSC Adv.*, 2019, **9**, 33761–33774.

12 A. R. Katritzky, A. S. Girgis, S. Slavov, S. R. Tala and I. Stoyanova-Slavova, QSAR modeling, synthesis and bioassay of diverse leukemia RPMI-8226 cell line active agents, *Eur. J. Med. Chem.*, 2010, **45**, 5183–5199.

13 A. R. Katritzky, R. Petrukhin, I. Petrukhina, A. Lomaka, D. B. Tatham and M. Karelson, CODESSA-Pro software manual 2005, pp. 48, 54, 63.

Table S1. Crystal data and structure refinement parameters for compound **8c.**

Chemical formula	$C_{28}H_{24}N_3O_4$
Mr	466.52
Crystal system, space group	Monoclinic, $P2_1/c$
Temperature (K)	298
a, *b*, *c* (Å)	6.2910 (5), 16.2664 (11), 23.223 (2)
β (°)	100.00 (18)
V (Å^3)	2340.4 (13)
Z	4
Radiation type	Mo *K*α
μ (mm^{-1})	0.09
Diffractometer	Nonius KappaCCD
Absorption correction	Multi-scan *DENZO*/*SCALEPACK* (Otwinowski & Minor, 1997)
T_{min}, T_{max}	1.00, 1.00
No. of measured, independent and observed [$I > 2.0\sigma(I)$] reflections	5196, 5196, 1340
$(\sin \theta/\lambda)_{max}$ (Å^{-1})	0.659
$R[F^2 > 2\sigma(F^2)]$, $wR(F^2)$, S	0.115, 0.042, 1.06
No. of reflections	1340
No. of parameters	316
$\Delta\rho_{max}$, $\Delta\rho_{min}$ (e Å^{-3})	0.56, −0.64
CCDC Number	CCDC 1988241

Table S2. Bond lengths (Å) of compound **8c**.

Geometric parameters	Bond lengths (Å)	Geometric parameters	Bond lengths (Å)
O1—C2	1.224 (9)	C16—C17	1.392 (11)
C2—N3	1.408 (9)	C17—C18	1.327 (12)
C2—C13	1.484 (12)	C18—C19	1.390 (12)
N3—C4	1.436 (9)	C19—C20	1.391 (11)
N3—C10	1.363 (9)	C21—N22	1.488 (10)
C4—C5	1.363 (11)	N22—C23	1.459 (8)
C4—C9	1.387 (9)	N22—C35	1.497 (9)
C5—C6	1.407 (12)	C23—C24	1.617 (10)
C6—C7	1.382 (12)	C23—C28	1.526 (11)
C7—C8	1.344 (12)	C24—O25	1.202 (9)
C8—C9	1.375 (12)	C24—N26	1.364 (10)
C10—O11	1.219 (8)	N26—C27	1.440 (11)
C10—C12	1.582 (11)	C27—C28	1.414 (10)
C12—C13	1.523 (9)	C27—C34	1.342 (12)
C12—C14	1.555 (10)	C28—C29	1.415 (11)
C12—C23	1.589 (10)	C29—C30	1.358 (11)
C14—C15	1.515 (10)	C30—O31	1.420 (10)
C14—C21	1.513 (9)	C30—C33	1.392 (10)
C15—C16	1.422 (11)	O31—C32	1.440 (9)
C15—C20	1.364 (10)	C33—C34	1.396 (12)

Table S3. Bond angles (°) for compound **8c**.

Geometric parameters	Bond angles (º)	Geometric parameters	Bond angles (º)
O1—C2—N3	122.1 (8)	C16—C17—C18	118.8 (9)
O1—C2—C13	126.0 (8)	C17—C18—C19	122.9 (9)
N3—C2—C13	111.7 (7)	C18—C19—C20	117.9 (8)
C2—N3—C4	126.5 (6)	C19—C20—C15	122.0 (8)
C2—N3—C10	110.5 (7)	C14—C21—N22	102.8 (6)
C4—N3—C10	123.0 (6)	C21—N22—C23	103.0 (6)

N3—C4—C5	119.2 (7)	C21—N22—C35	113.0 (6)
N3—C4—C9	123.0 (7)	C23—N22—C35	114.3 (5)
C5—C4—C9	117.7 (8)	C12—C23—N22	103.4 (5)
C4—C5—C6	120.2 (8)	C12—C23—C24	109.9 (6)
C5—C6—C7	120.8 (9)	N22—C23—C24	110.7 (6)
C6—C7—C8	118.3 (9)	C12—C23—C28	118.5 (6)
C7—C8—C9	121.4 (8)	N22—C23—C28	110.5 (6)
C4—C9—C8	121.5 (8)	C24—C23—C28	103.9 (6)
N3—C10—O11	124.8 (7)	C23—C24—O25	127.4 (7)
N3—C10—C12	108.3 (6)	C23—C24—N26	105.6 (7)
O11—C10—C12	126.9 (7)	O25—C24—N26	127.0 (7)
C10—C12—C13	103.5 (6)	C24—N26—C27	112.4 (7)
C10—C12—C14	109.2 (5)	N26—C27—C28	111.3 (7)
C13—C12—C14	118.6 (6)	N26—C27—C34	128.2 (9)
C10—C12—C23	108.2 (6)	C28—C27—C34	120.0 (8)
C13—C12—C23	114.7 (5)	C23—C28—C27	106.7 (6)
C14—C12—C23	102.4 (6)	C23—C28—C29	133.2 (7)
C12—C13—C2	104.1 (6)	C27—C28—C29	120.0 (8)
C12—C14—C15	119.0 (5)	C28—C29—C30	118.7 (8)
C12—C14—C21	105.5 (6)	C29—C30—O31	123.6 (8)
C15—C14—C21	111.3 (6)	C29—C30—C33	120.7 (9)
C14—C15—C16	123.0 (7)	O31—C30—C33	115.7 (8)
C14—C15—C20	119.8 (7)	C30—O31—C32	119.9 (7)
C16—C15—C20	117.2 (8)	C30—C33—C34	120.5 (8)
C15—C16—C17	121.0 (8)	C33—C34—C27	119.8 (8)

Table S4. Descriptor of the BMLR-QSAR model for the tested compounds as AChE inhibitors.

Entry	ID	Coefficient	*s*	*t*	Descriptor
1	0	-47.747	5.290	-9.026	Intercept
2	D_1	466.922	52.917	8.824	Min. (>0.1) bond order for atom C

3	D_2	85.5021	11.191	7.640	HA dependent HDCA-2/SQRT(TMSA) (MOPAC PC)
4	D_3	-0.164864	0.027	-6.179	Square root of surface area for atom C

$N = 12$, $n = 3$, $R^2 = 0.923$, R^2cvOO = 0.882, R^2cvMO = 0.904, $F = 31.948$, $s^2 = 0.026$

$\log(IC_{50}, \mu M) = –47.747 + (466.922 \times D_1) + (85.5021 \times D_2) – (0.164864 \times D_3)$

Table S5. Observed and estimated activity values for the tested compounds as AChE inhibitors according to the BMLR-QSAR model.

Entry	Compd.	Observed $\log(IC_{50}, \mu M)$	Observed IC_{50}, μM	Estimated $\log(IC_{50}, \mu M)$	Estimated IC_{50}, μM	Error[a]
1	**8a**	2.06662	116.58	2.1139	129.99	-13.41
2	**8b**	1.13354	13.6	1.12937	13.47	0.13
3	**8c**	2.01237	102.89	1.92628	84.39	18.50
4	**8d**	1.38561	24.3	1.01604	10.38	13.92
5	**8e**	0.525045	3.35	0.512188	3.25	0.10
6	**8f**	1.08814	12.25	1.03874	10.93	1.32
7	**8g**	0.498311	3.15	0.693192	4.93	-1.78
8	**8h**	0.797268	6.27	0.863375	7.30	-1.03
9	**8i**	1.59561	39.41	1.63445	43.10	-3.69
10	**8j**	1.32181	20.98	1.3381	21.78	-0.80
11	**8k**	1.14395	13.93	1.16877	14.75	-0.82
12	**8l**	1.34183	21.97	1.47568	29.90	-7.93

[a] Error is the difference between the observed and estimated property values in μM values.

Table S6. Molecular descriptor values of the BMLR-QSAR model for the synthesized compounds as AChE inhibitors.

Entry	Compd.	Descriptors[a]		
		D_1	D_2	D_3
1	**8a**	0.10497	0.1247	59.52144

2	**8b**	0.10146	0.11974	52.98071
3	**8c**	0.10225	0.13039	55.9192
4	**8d**	0.10221	0.11744	54.61403
5	**8e**	0.10232	0.11715	57.81774
6	**8f**	0.10114	0.12772	56.75511
7	**8g**	0.10162	0.11903	55.71342
8	**8h**	0.10053	0.12459	54.48833
9	**8i**	0.10193	0.12444	53.67995
10	**8j**	0.10352	0.11458	54.88521
11	**8k**	0.10199	0.10763	47.96379
12	**8l**	0.10252	0.12131	54.69956

[a] D_1 = Min. (>0.1) bond order for atom C, D_2 = HA dependent HDCA-2/SQRT(TMSA) (MOPAC PC), D_3 = Square root of Surface Area for atom C.

Table S7. Descriptor of the BMLR-QSAR model for the tested compounds as BChE inhibitors.

Entry	ID	Coefficient	*s*	*t*	Descriptor
1	0	-695.08	92.732	-7.496	Intercept
2	D_1	10.5556	0.655	16.119	ZX Shadow / ZX Rectangle
3	D_2	2.22981	0.301	7.400	Max. atomic state energy for atom O
4	D_3	0.0220702	0.004	6.045	Shadow plane XY

$N = 12$, $n = 3$, $R^2 = 0.979$, R^2cvOO = 0.936, R^2cvMO = 0.956, $F = 122.564$, $s^2 = 0.005$

$\log(IC_{50}, \mu M) = -695.08 + (10.5556 \times D_1) + (2.22981 \times D_2) + (0.0220702 \times D_3)$

Table S8. Observed and estimated activity values for the tested compounds as BChE inhibitors according to the BMLR-QSAR model.

Entry	Compd.	Observed log(IC_{50}, μM)	Observed IC_{50}, μM	Estimated log(IC_{50}, μM)	Estimated IC_{50}, μM	Error[a]
1	**8a**	1.91249	81.75	1.83852	68.95	12.80
2	**8b**	1.62788	42.45	1.69097	49.09	-6.64

3	**8c**	1.85582	71.75	1.80826	64.31	7.44
4	**8d**	1.32899	21.33	1.43514	27.24	-5.91
5	**8e**	0.750508	5.63	0.678732	4.77	0.86
6	**8f**	1.3032	20.1	1.26066	18.22	1.88
7	**8g**	0.675778	4.74	0.731632	5.39	-0.65
8	**8h**	0.727541	5.34	0.788595	6.15	-0.81
9	**8i**	1.54555	35.12	1.58656	38.60	-3.48
10	**8j**	1.1329	13.58	1.08186	12.07	1.51
11	**8k**	1.00945	10.22	0.960594	9.13	1.09
12	**8l**	1.55072	35.54	1.55929	36.25	-0.71

[a] Error is the difference between the observed and estimated property values in μM values.

Table S9. Molecular descriptor values of the BMLR-QSAR model for the synthesized compounds as BChE inhibitors.

Entry	Compd.	Descriptors[a]		
		D_1	D_2	D_3
1	**8a**	0.66053	308.443	98.66
2	**8b**	0.70783	308.2048	93.42
3	**8c**	0.68051	308.2503	107.2
4	**8d**	0.64082	308.3275	101.48
5	**8e**	0.59553	308.2474	96.96
6	**8f**	0.65798	308.2169	96.54
7	**8g**	0.57674	308.25	108.08
8	**8h**	0.60684	308.2241	98.88
9	**8i**	0.64727	308.312	106.82
10	**8j**	0.62421	308.2081	105.48
11	**8k**	0.61271	308.1816	108.16
12	**8l**	0.61341	308.3524	117.7

[a] D_1 = ZX Shadow / ZX Rectangle, D_2 = Max. atomic state energy for atom O, D_3 = Shadow plane XY.

Table S10. Observed and estimated activity values for the tested compounds **8a–l** as AChE inhibitors according to the 3D-pharmacophore model.

Entry	Compd.	Observed log($IC_{50,}$ μM)	Observed $IC_{50,}$ μM	Estimated log($IC_{50,}$ μM)	Estimated $IC_{50,}$ μM
1	**8a**	2.06662	116.6	2.14848	140.8
2	**8b**	1.13354	13.6	1.08505	12.2
3	**8c**	2.01237	102.9	1.46648	29.3
4	**8d**	1.38561	24.3	0.922511	8.4
5	**8e**	0.525045	3.4	0.805378	6.4
6	**8f**	1.08814	12.3	1.11632	13.1
7	**8g**	0.498311	3.2	0.642735	4.4
8	**8h**	0.797268	6.3	0.982	9.6
9	**8i**	1.59561	39.4	1.43479	27.2
10	**8j**	1.32181	21.0	1.83182	67.9
11	**8k**	1.14395	13.9	1.20337	16.0
12	**8l**	1.34183	22.0	0.97118	9.4

Table S11. Observed and estimated activity values for the tested compounds **8a–l** as BChE inhibitors according to the 3D-pharmacophore model.

Entry	Compd.	Observed log($IC_{50,}$ μM)	Observed $IC_{50,}$ μM	Estimated log($IC_{50,}$ μM)	Estimated $IC_{50,}$ μM
1	**8a**	1.91249	81.8	1.95644	90.5
2	**8b**	1.62788	42.5	1.48946	30.9
3	**8c**	1.85582	71.7	1.42828	26.8
4	**8d**	1.32899	21.3	1.21379	16.4
5	**8e**	0.750508	5.6	1.05258	11.3
6	**8f**	1.3032	20.1	1.11712	13.1
7	**8g**	0.675778	4.7	0.998164	10.0
8	**8h**	0.727541	5.3	1.06305	11.6
9	**8i**	1.54555	35.1	1.32427	21.1
10	**8j**	1.1329	13.6	0.975985	9.5

11	**8k**	1.00945	10.2	1.01392	10.3
12	**8l**	1.55072	35.5	1.12307	13.3

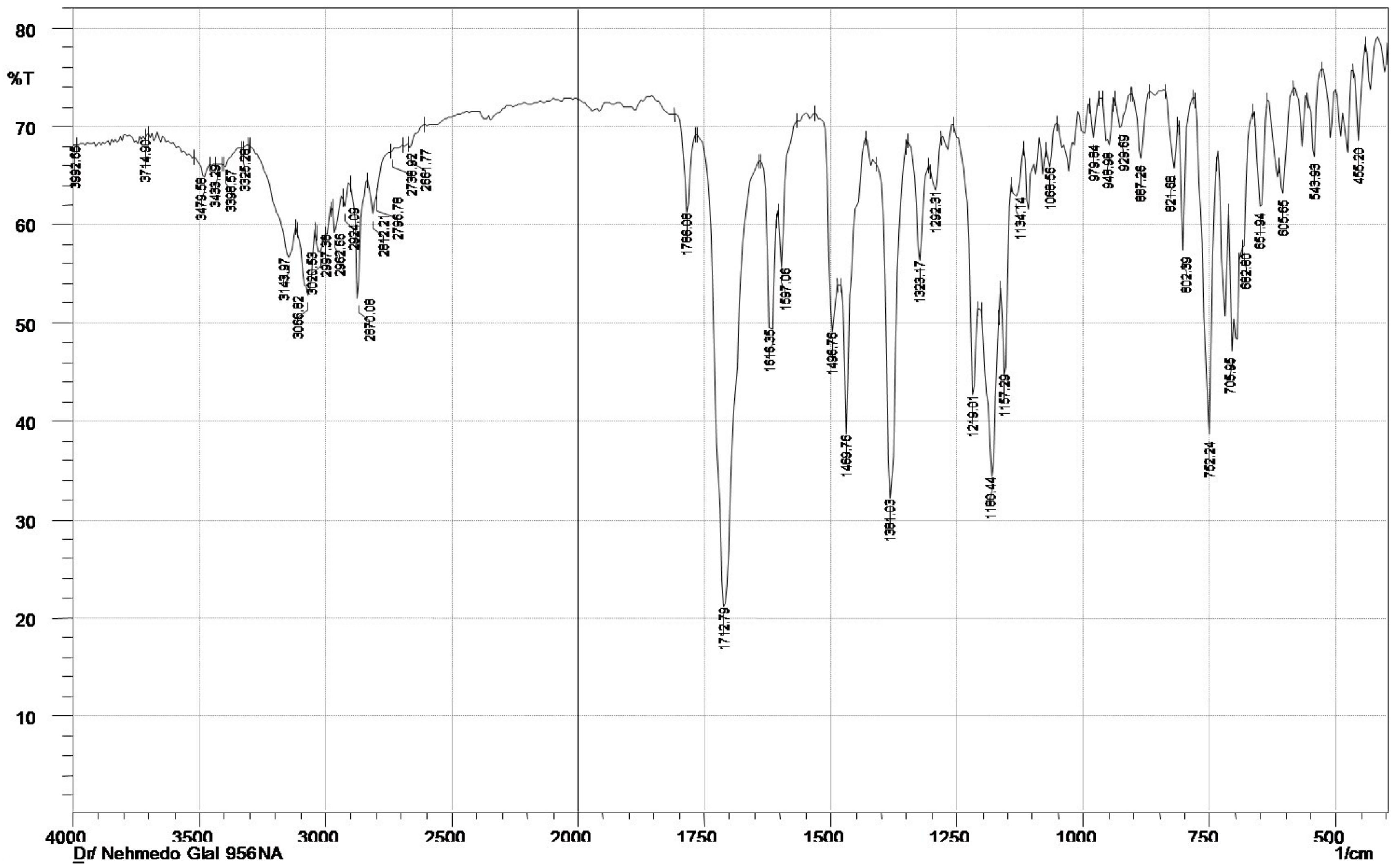

Fig. S1. IR spectrum of compound **8a** (KBr pellet).

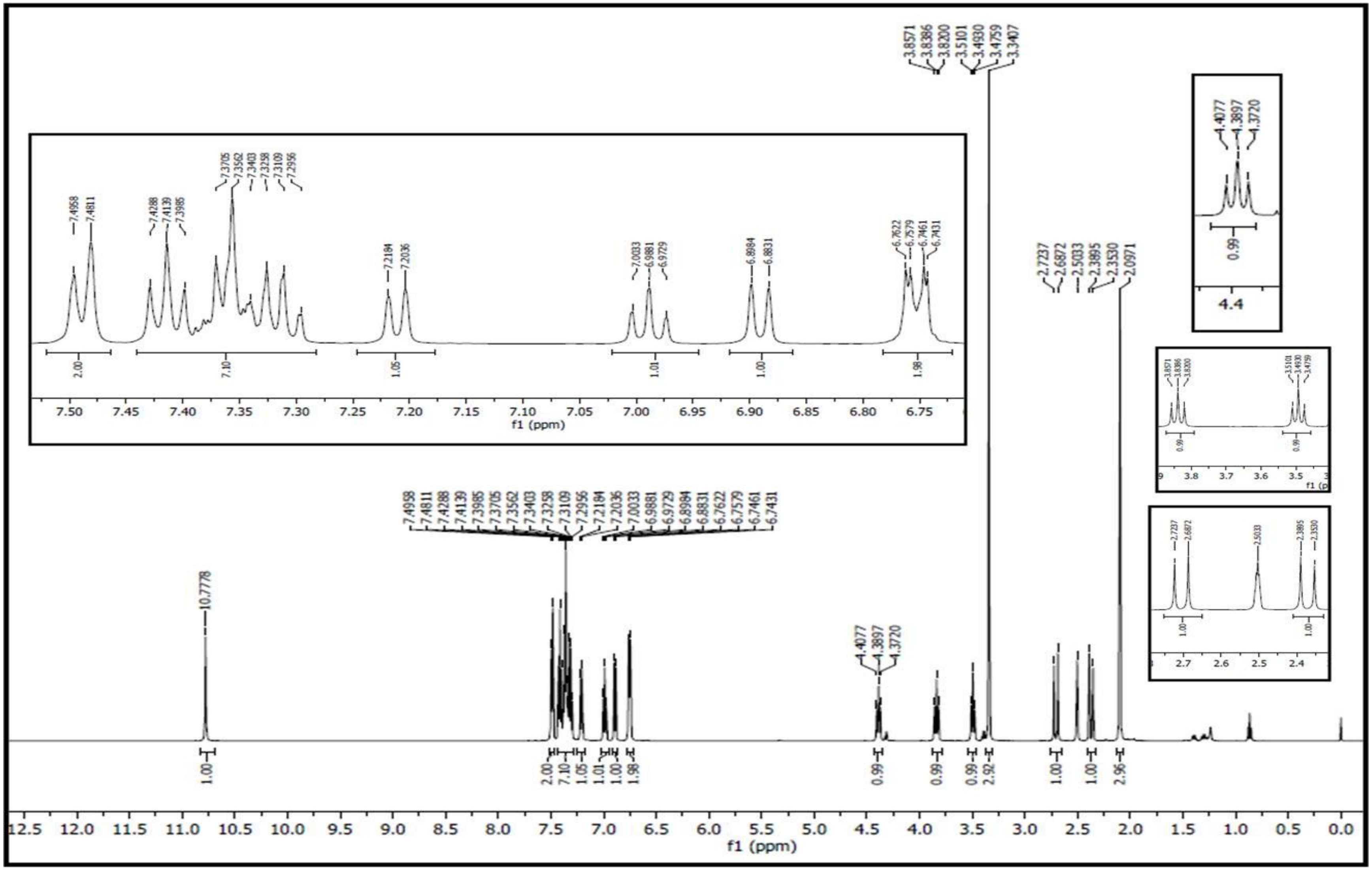

Fig. S2. ^{1}H-NMR spectrum of compound **8a** in DMSO-d_6.

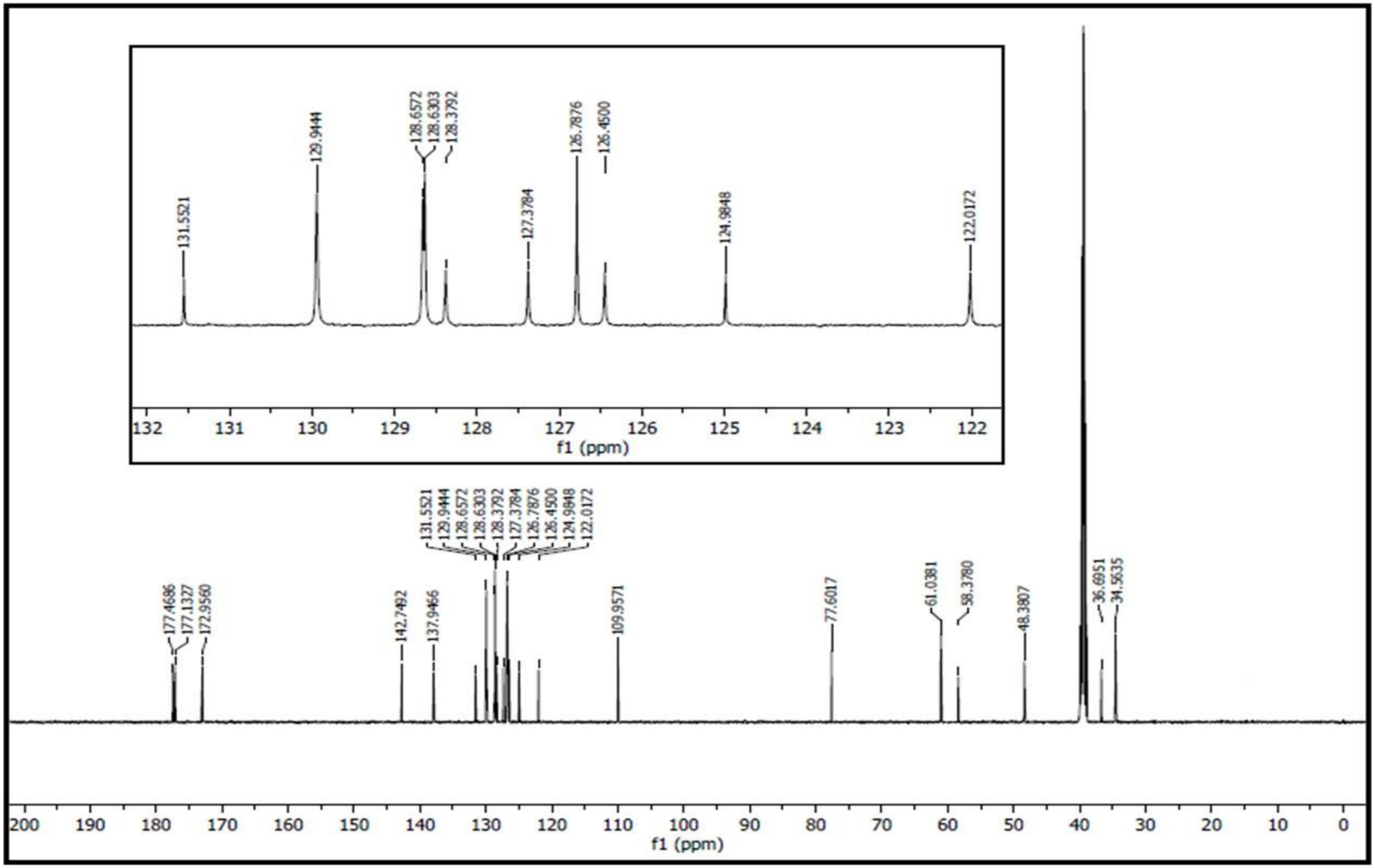

Fig. S3. ^{13}C-NMR spectrum of compound **8a** in DMSO-d_6.

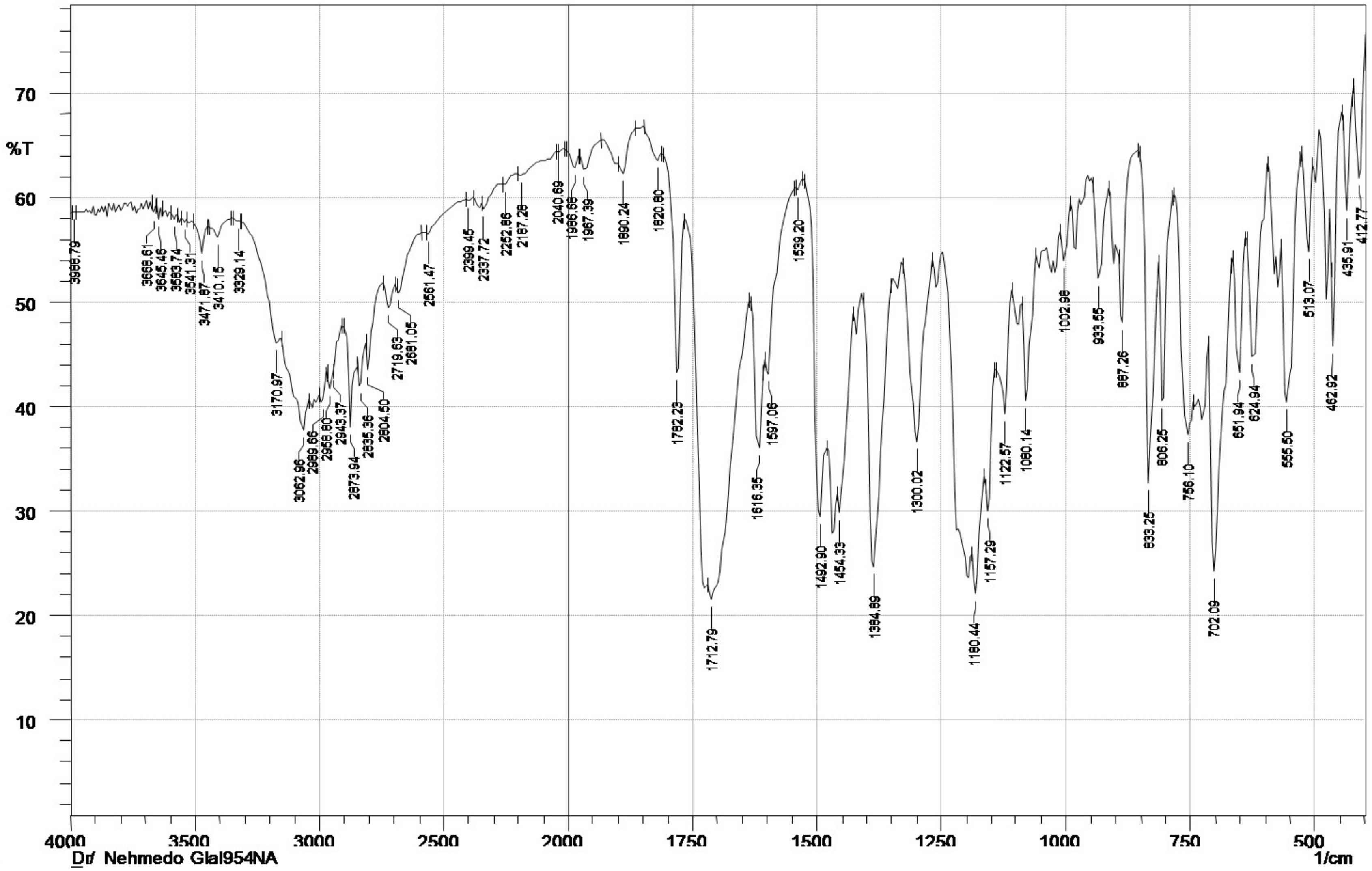

Fig. S4. IR spectrum of compound **8b** (KBr pellet).

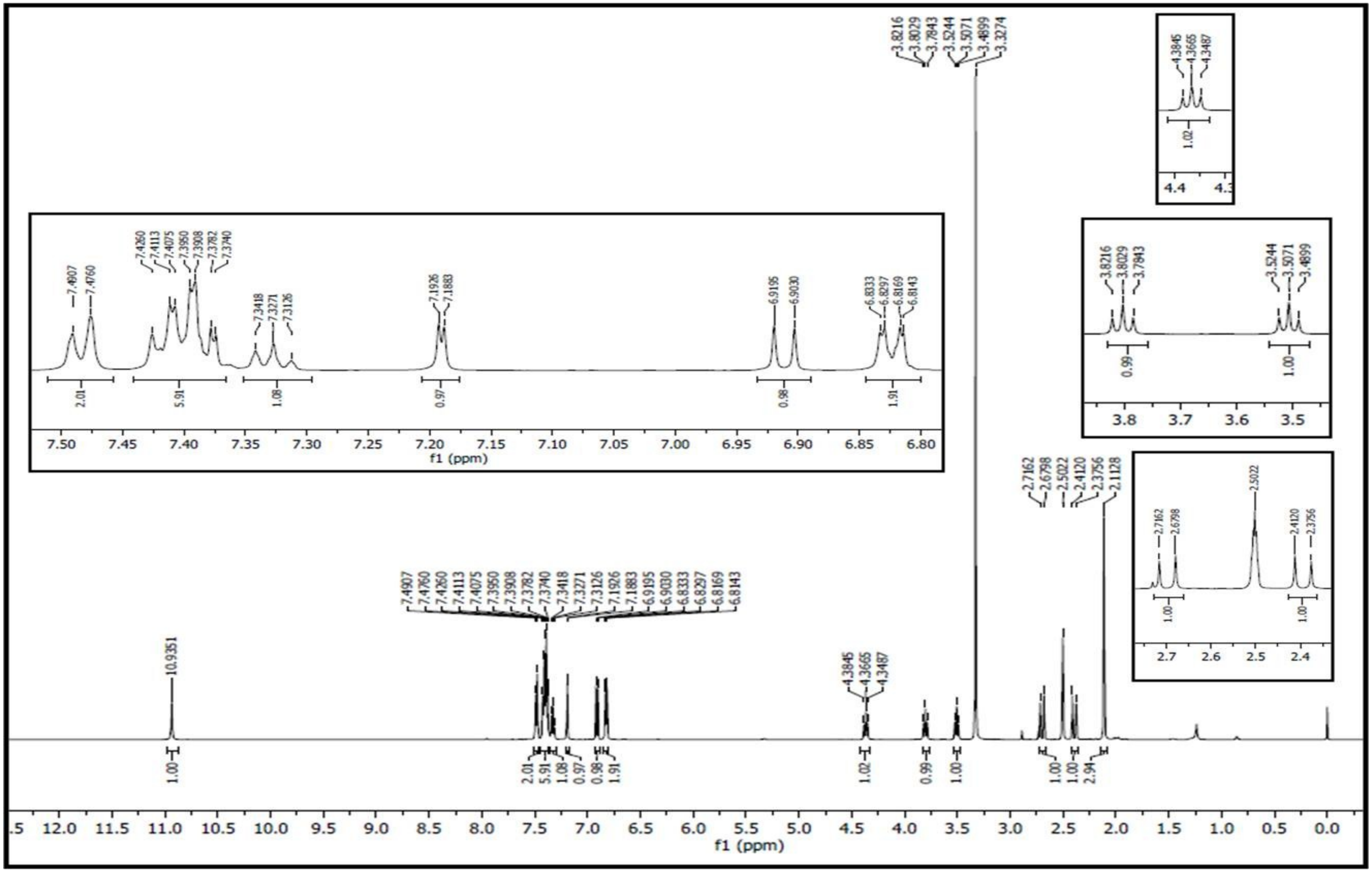

Fig. S5. ^{1}H-NMR spectrum of compound **8b** in DMSO-d_6.

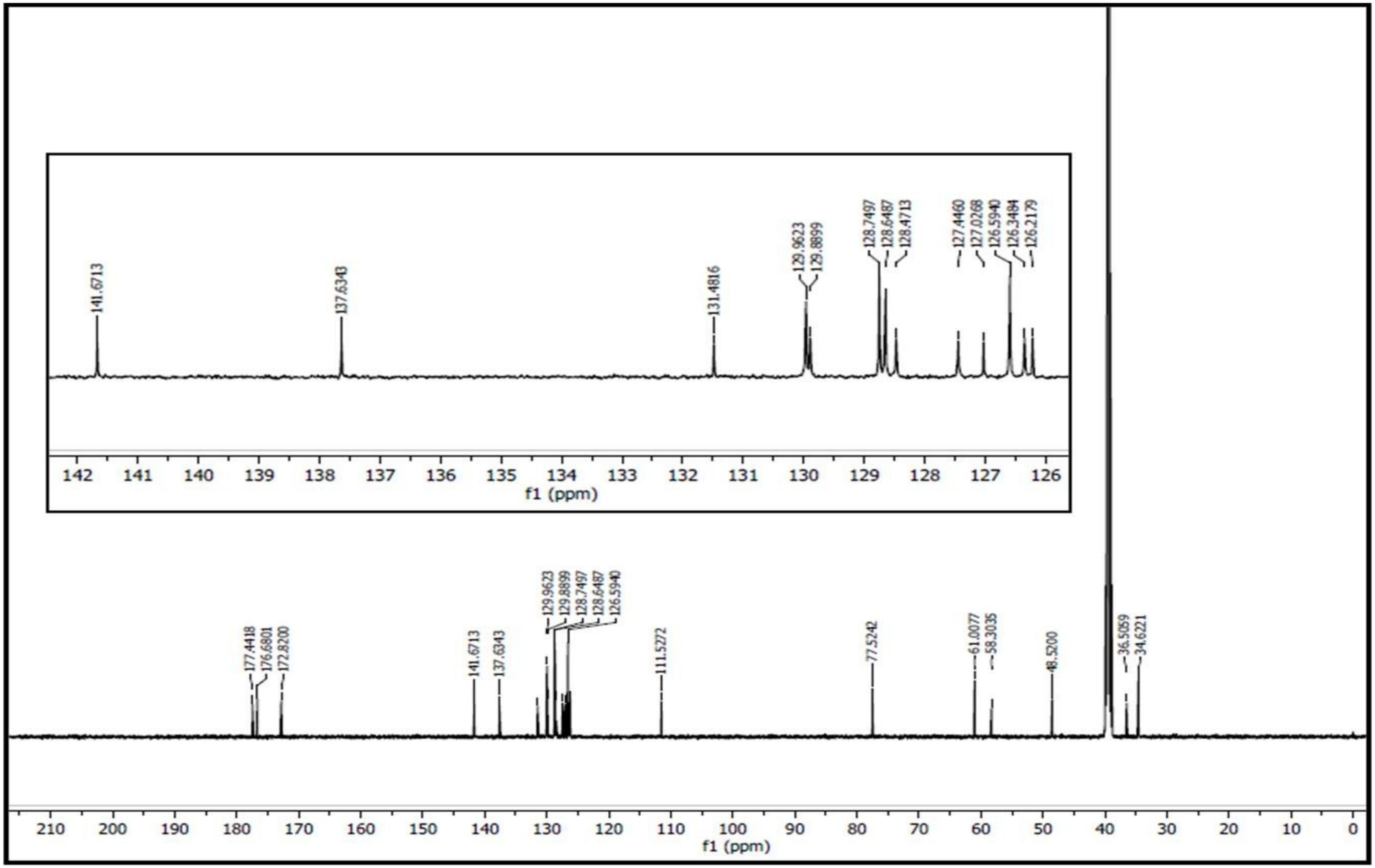

Fig. S6. ^{13}C-NMR spectrum of compound **8b** in DMSO-d_6.

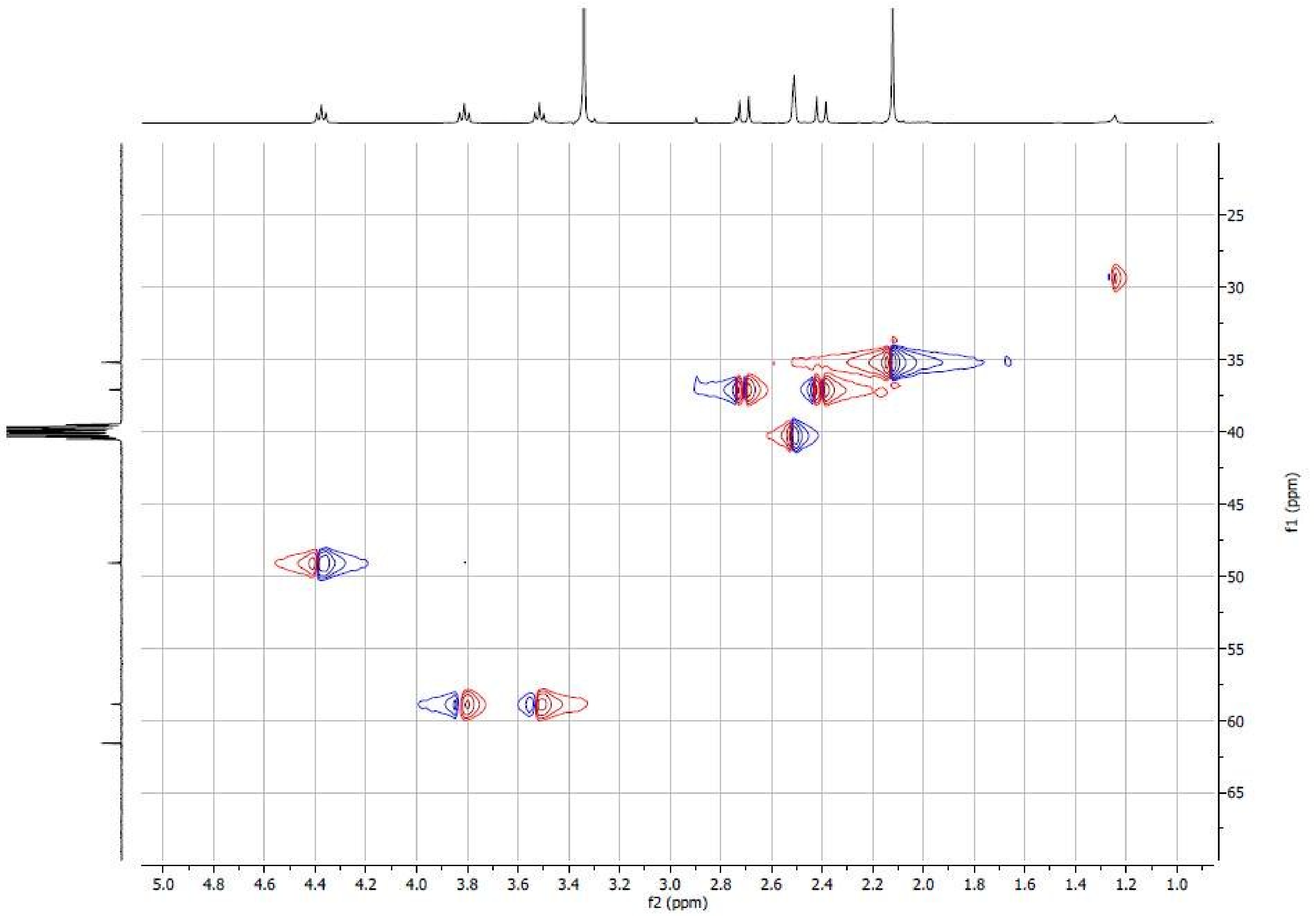

Fig. S7. HSQC spectrum of compound **8b** in DMSO-d_6.

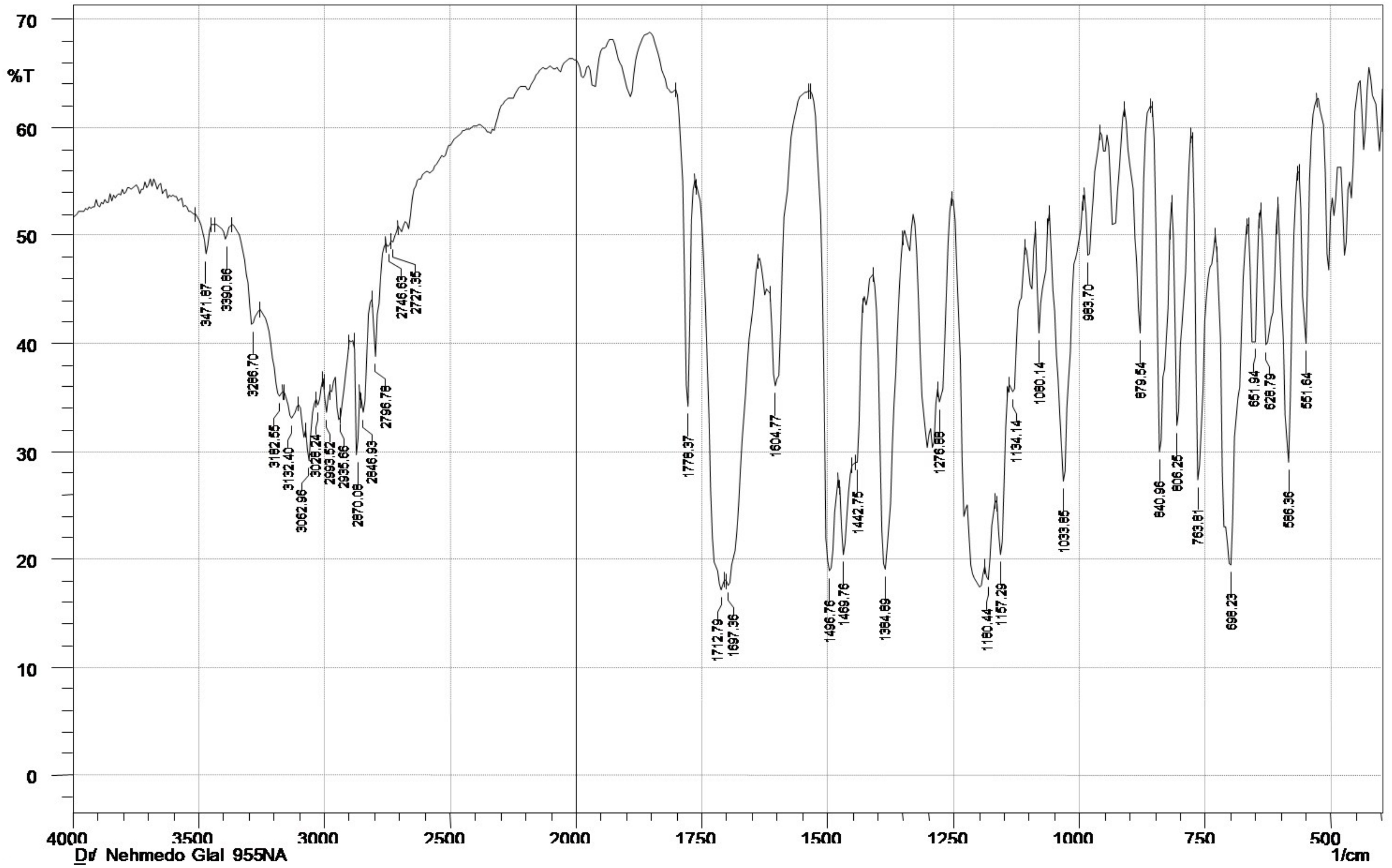

Fig. S8. IR spectrum of compound **8c** (KBr pellet).

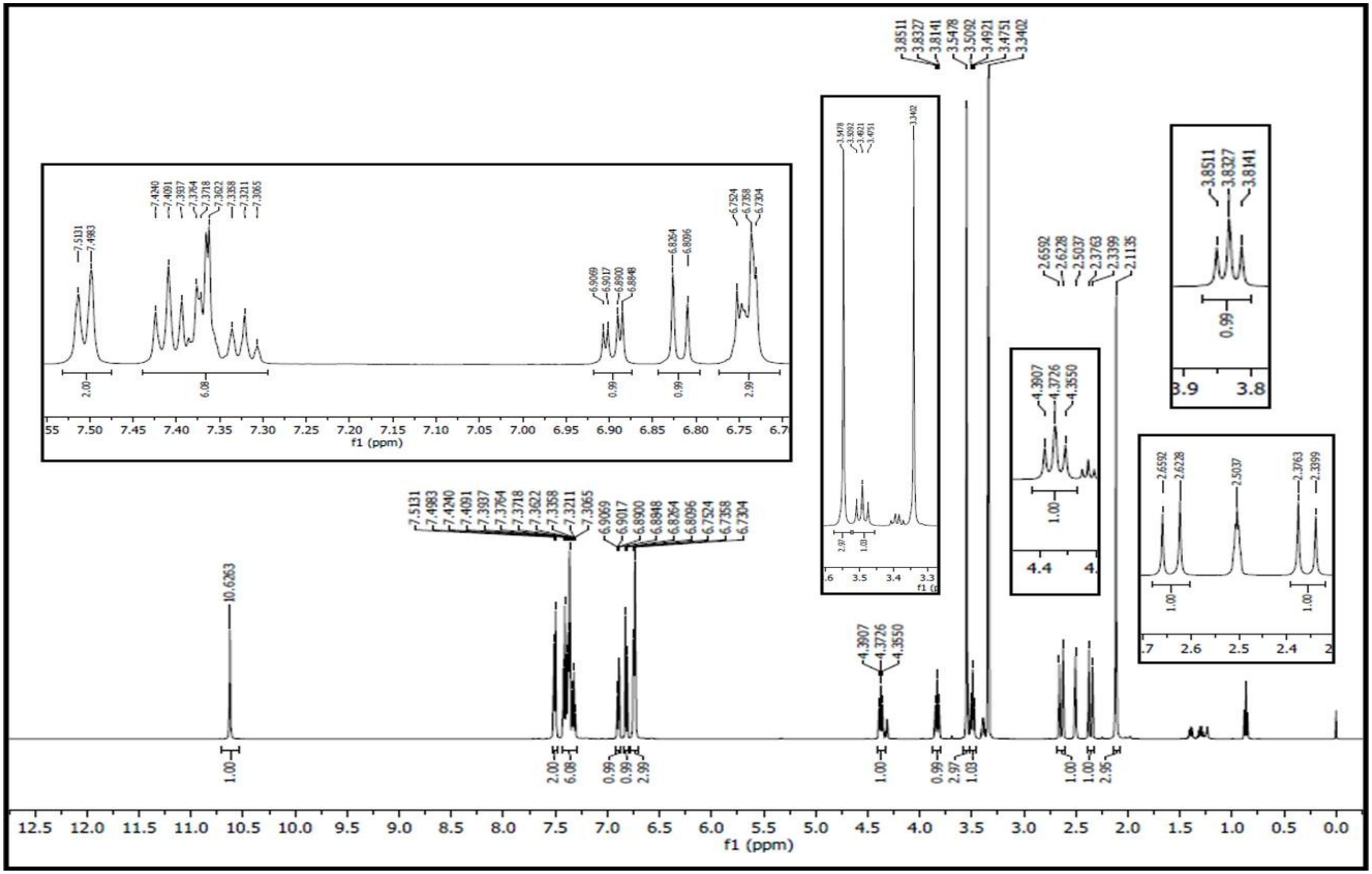

Fig. S9. ^{1}H-NMR spectrum of compound **8c** in DMSO-d_6.

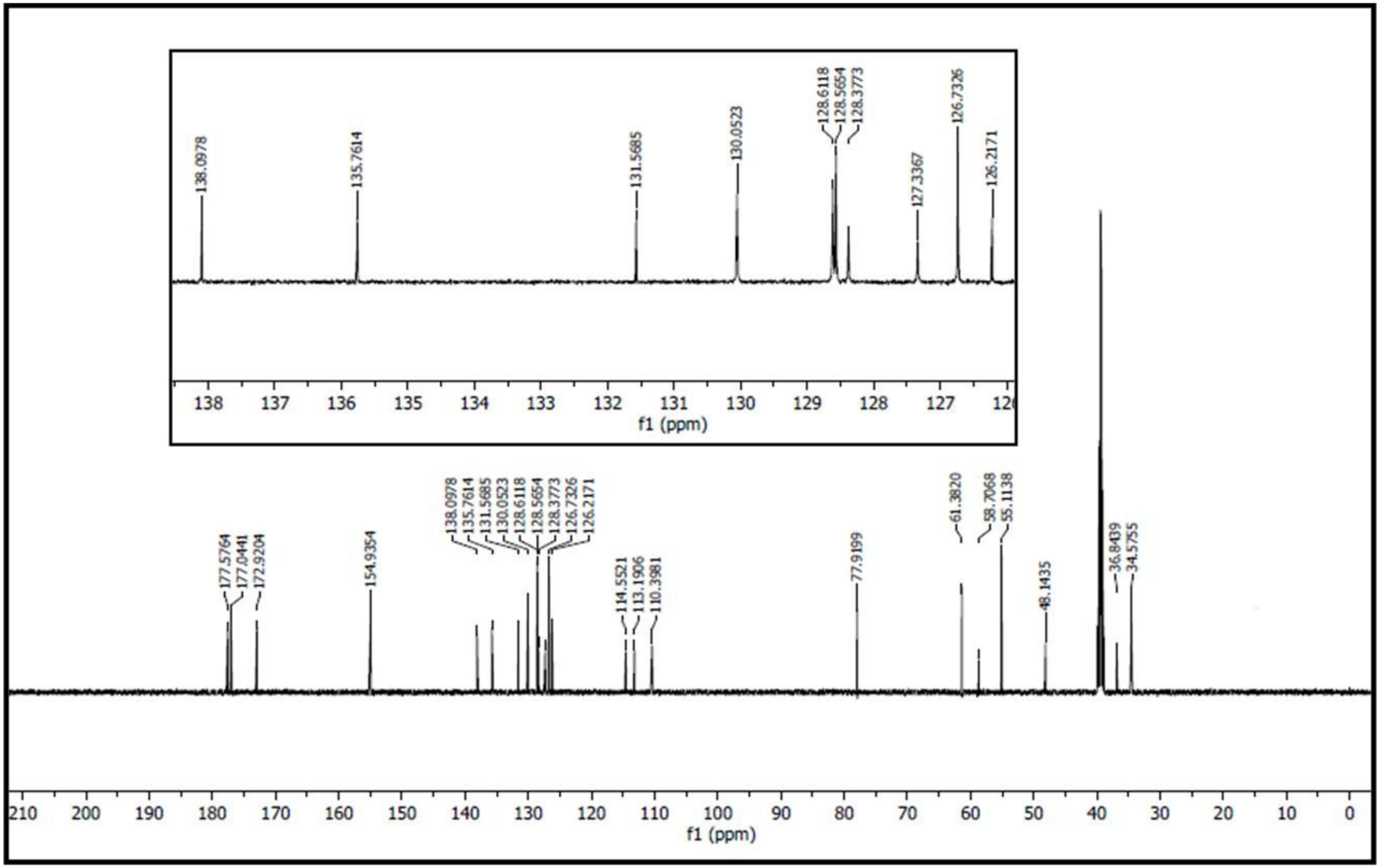

Fig. S10. ^{13}C-NMR spectrum of compound **8c** in DMSO-d_6.

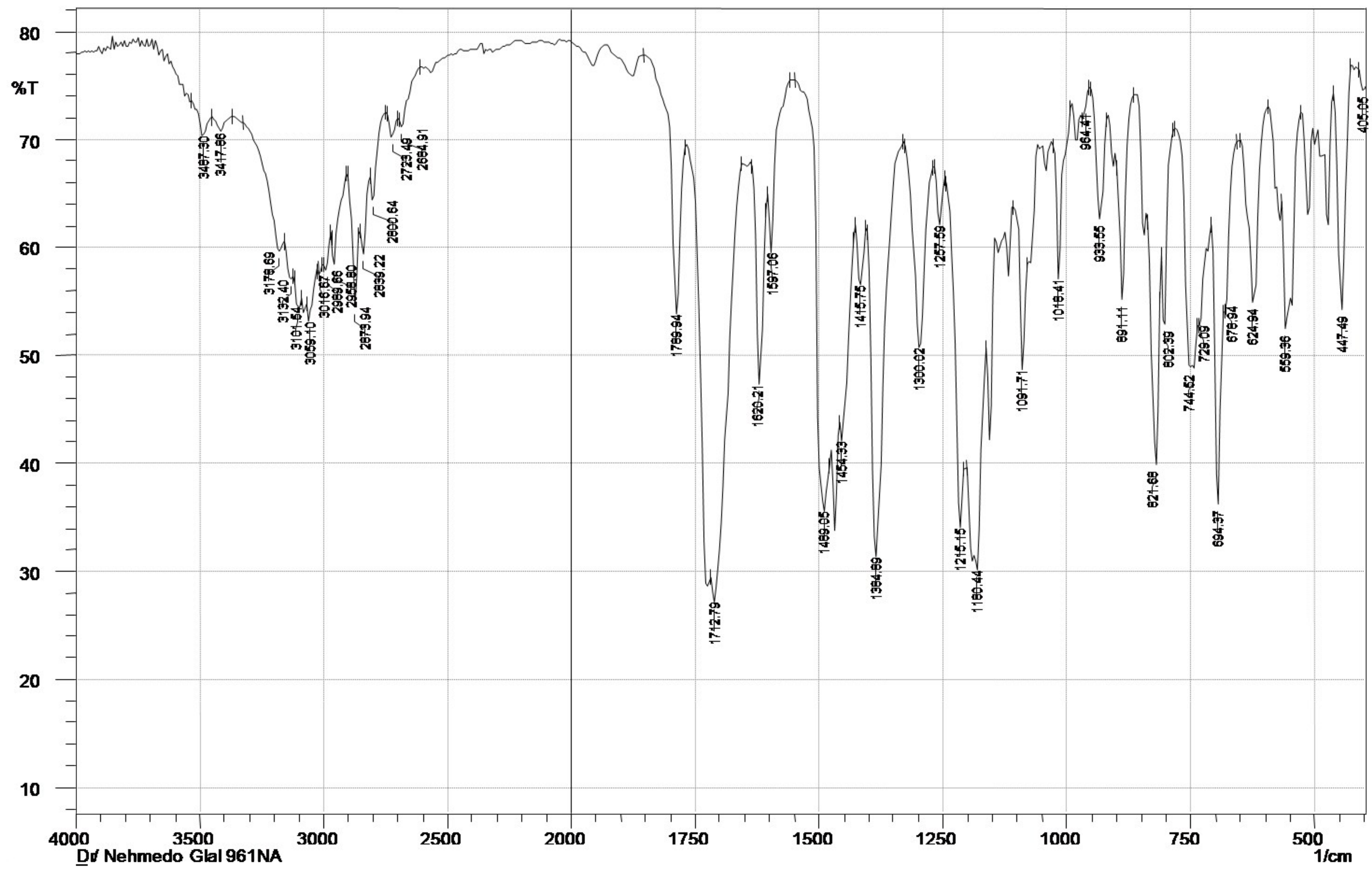

Fig. S11. IR spectrum of compound **8d** (KBr pellet).

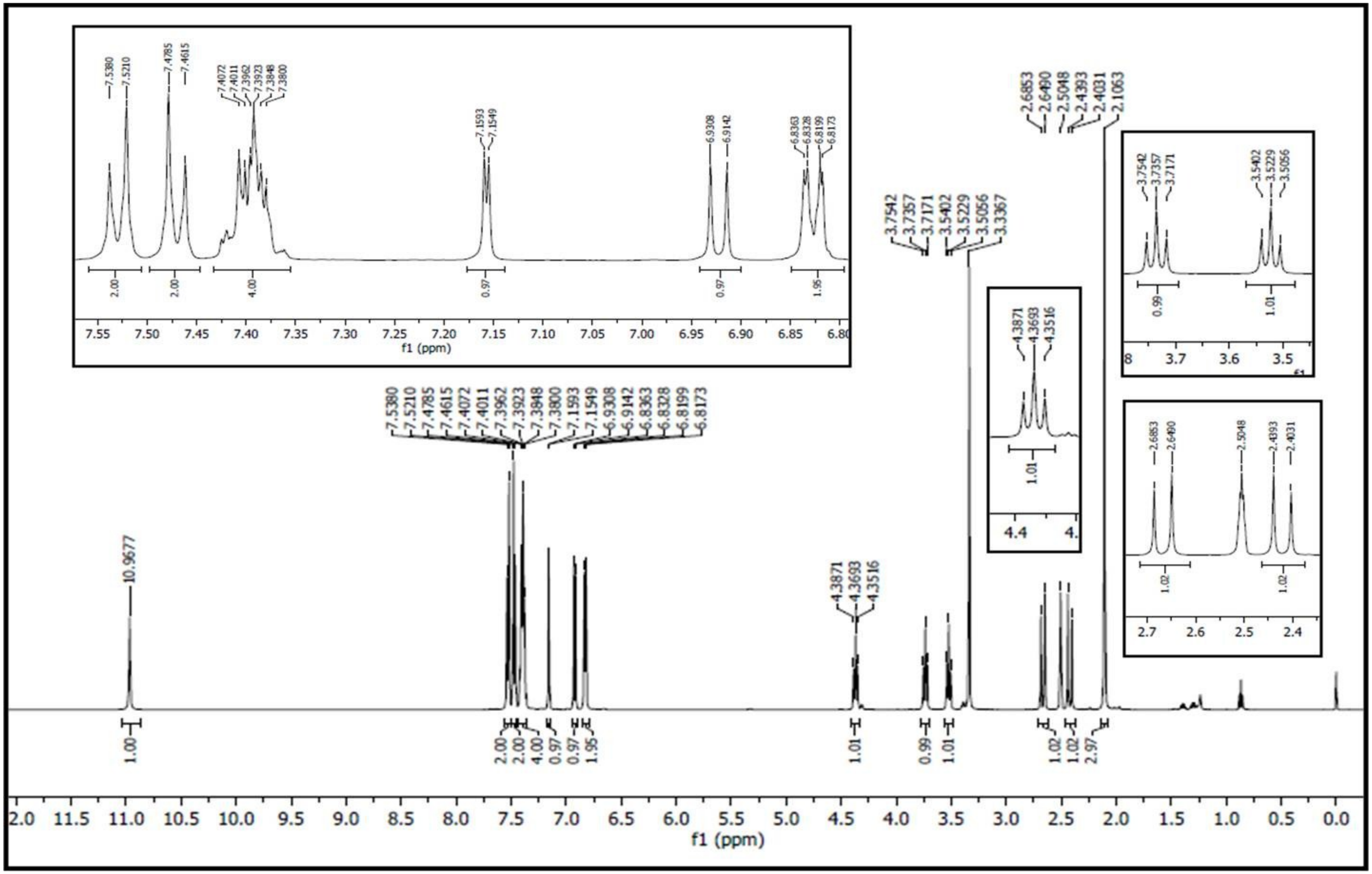

Fig. S12. ^{1}H-NMR spectrum of compound **8d** in DMSO-d_6.

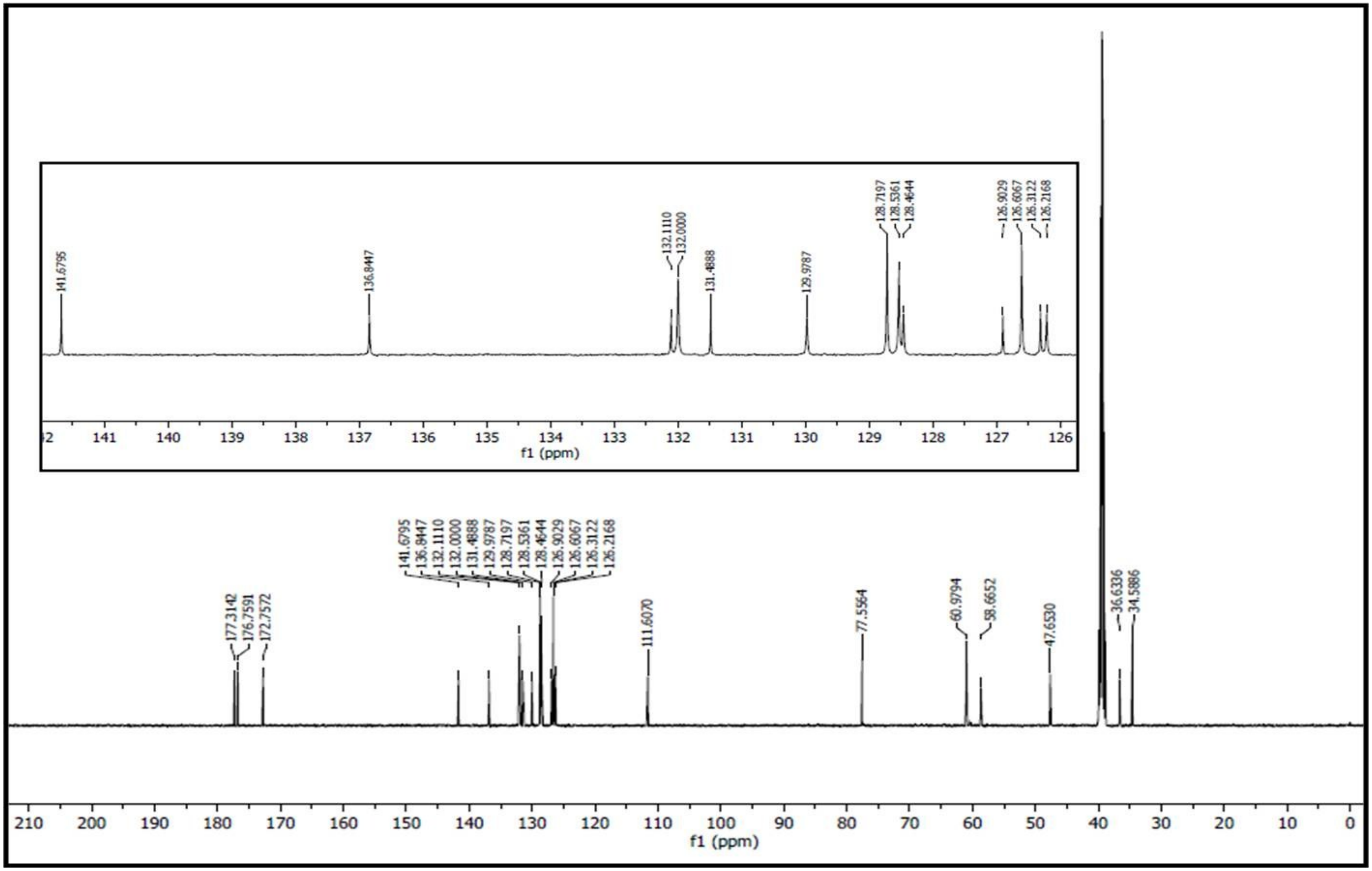

Fig. S13. ^{13}C-NMR spectrum of compound **8d** in DMSO-d_6.

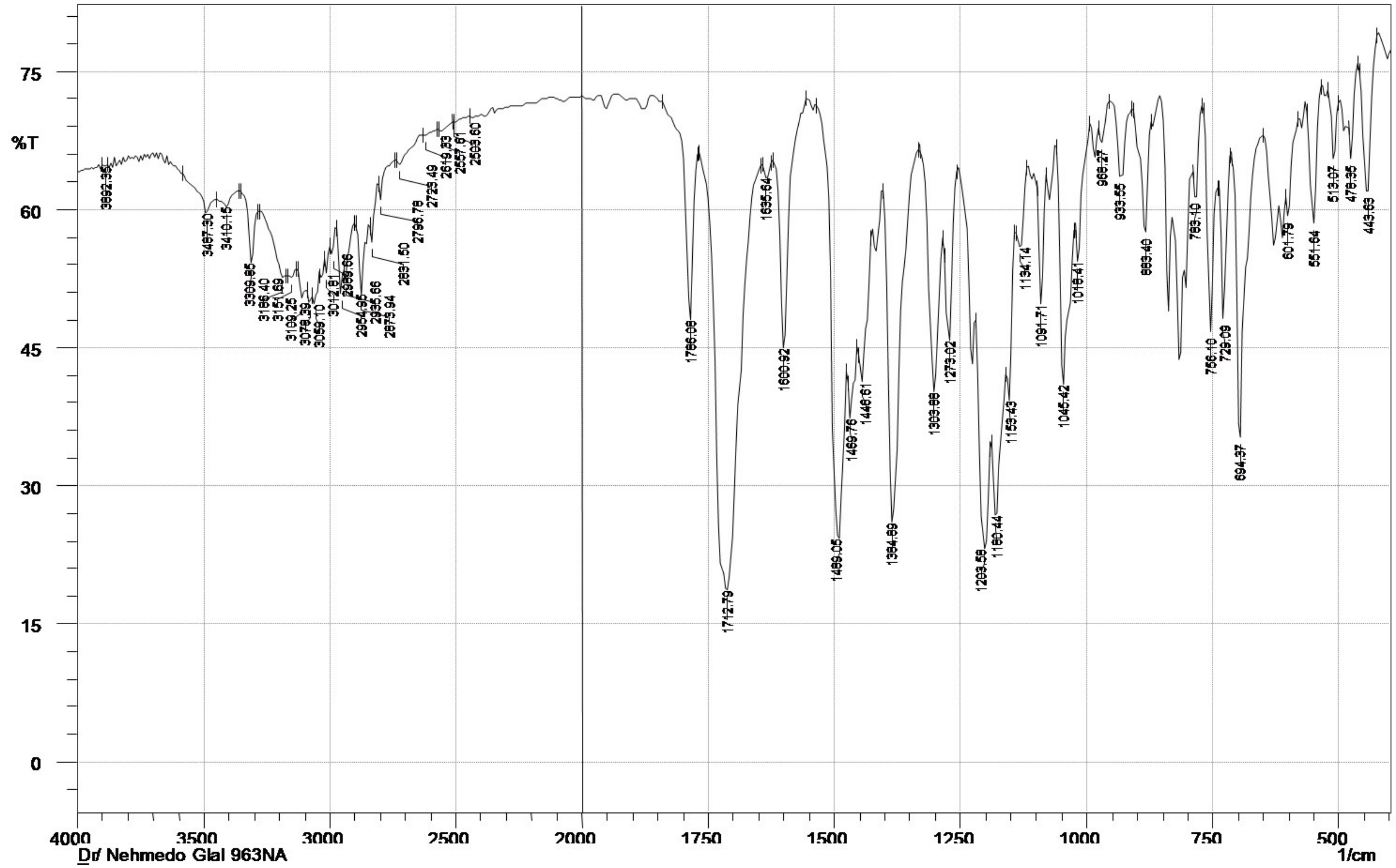

Fig. S14. IR spectrum of compound **8e** (KBr pellet).

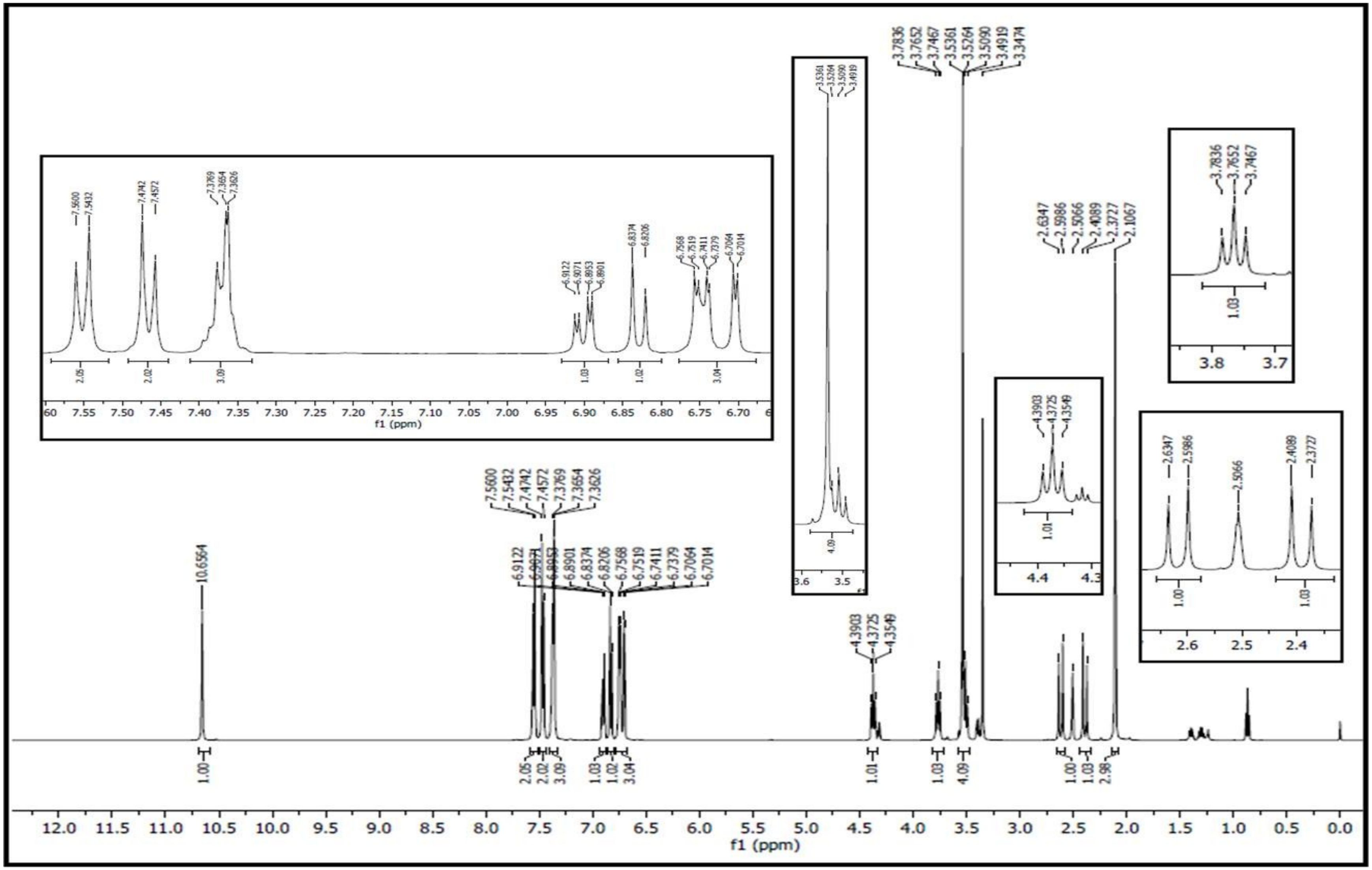

Fig. S15. ^{1}H-NMR spectrum of compound **8e** in DMSO-d_6.

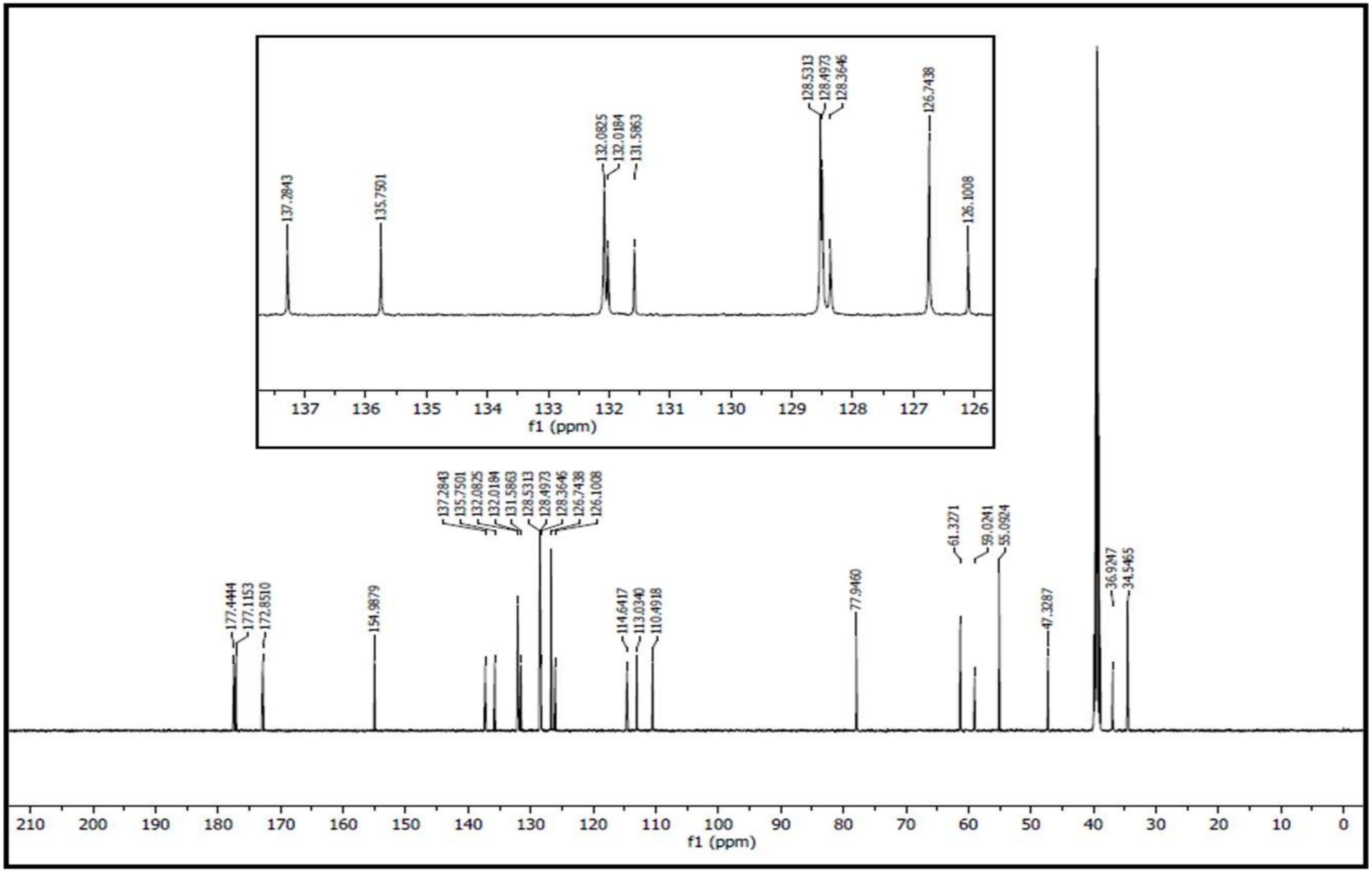

Fig. S16. ^{13}C-NMR spectrum of compound **8e** in DMSO-d_6.

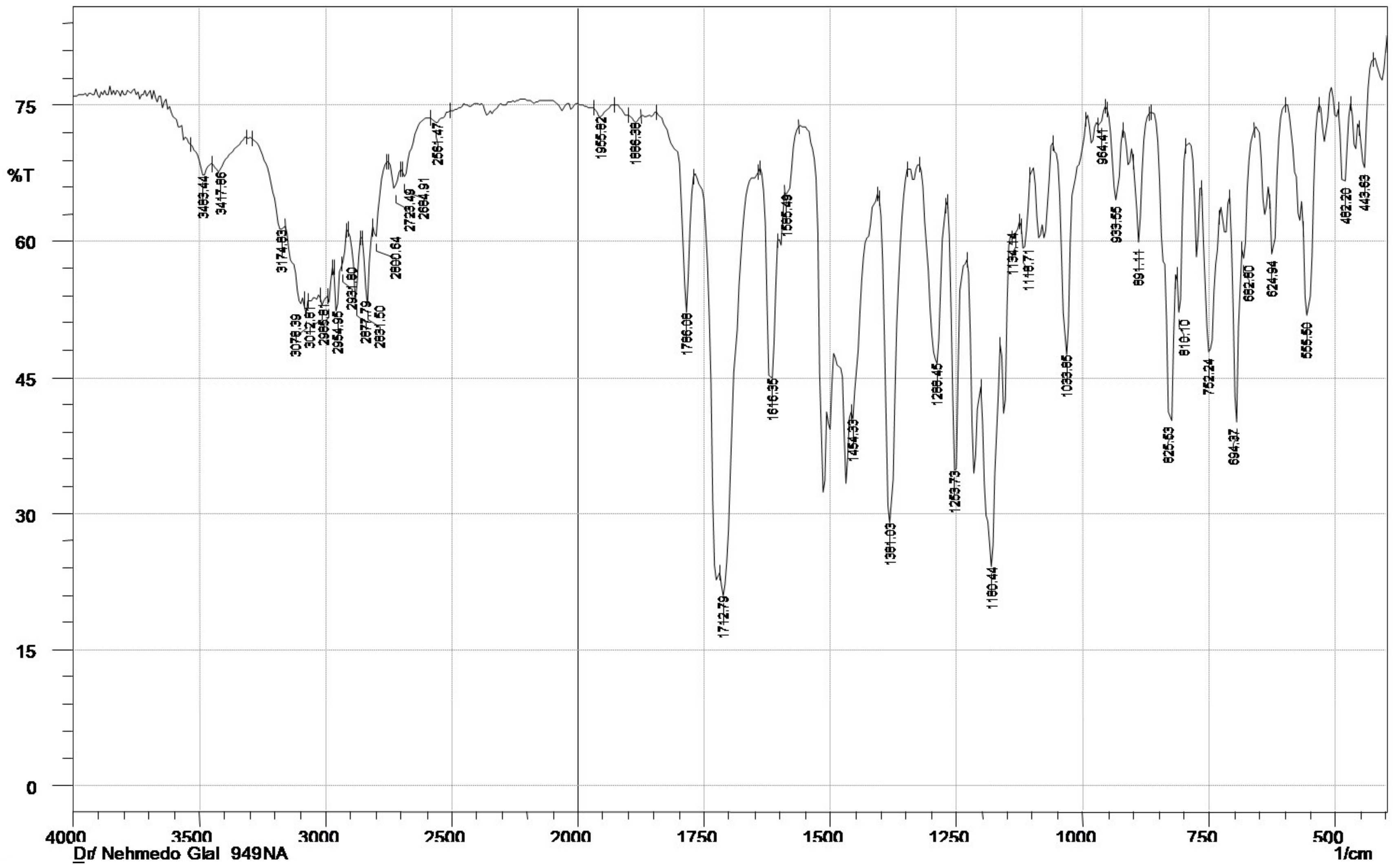

Fig. S17. IR spectrum of compound **8f** (KBr pellet).

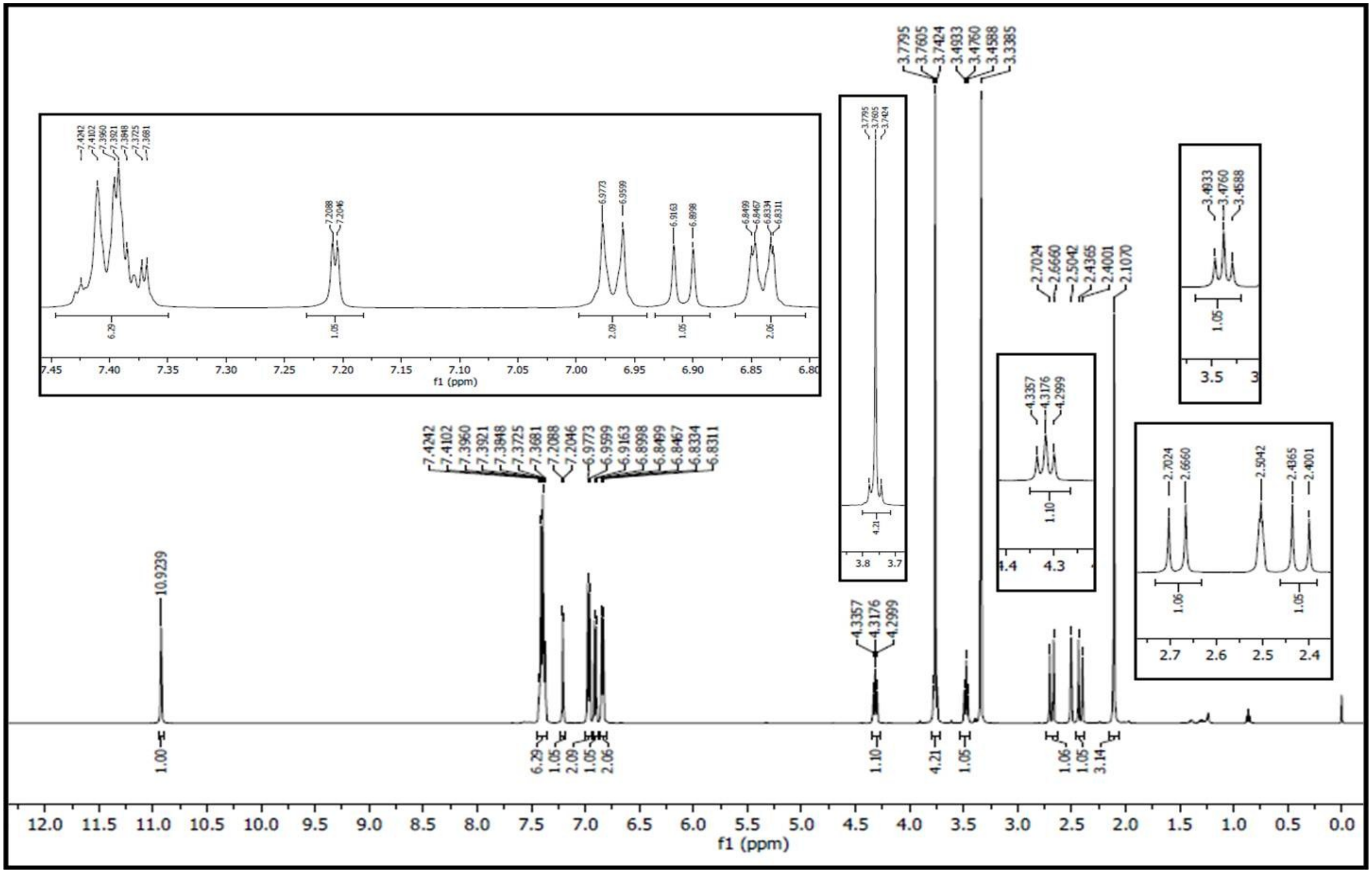

Fig. S18. ^{1}H-NMR spectrum of compound **8f** in DMSO-d_6.

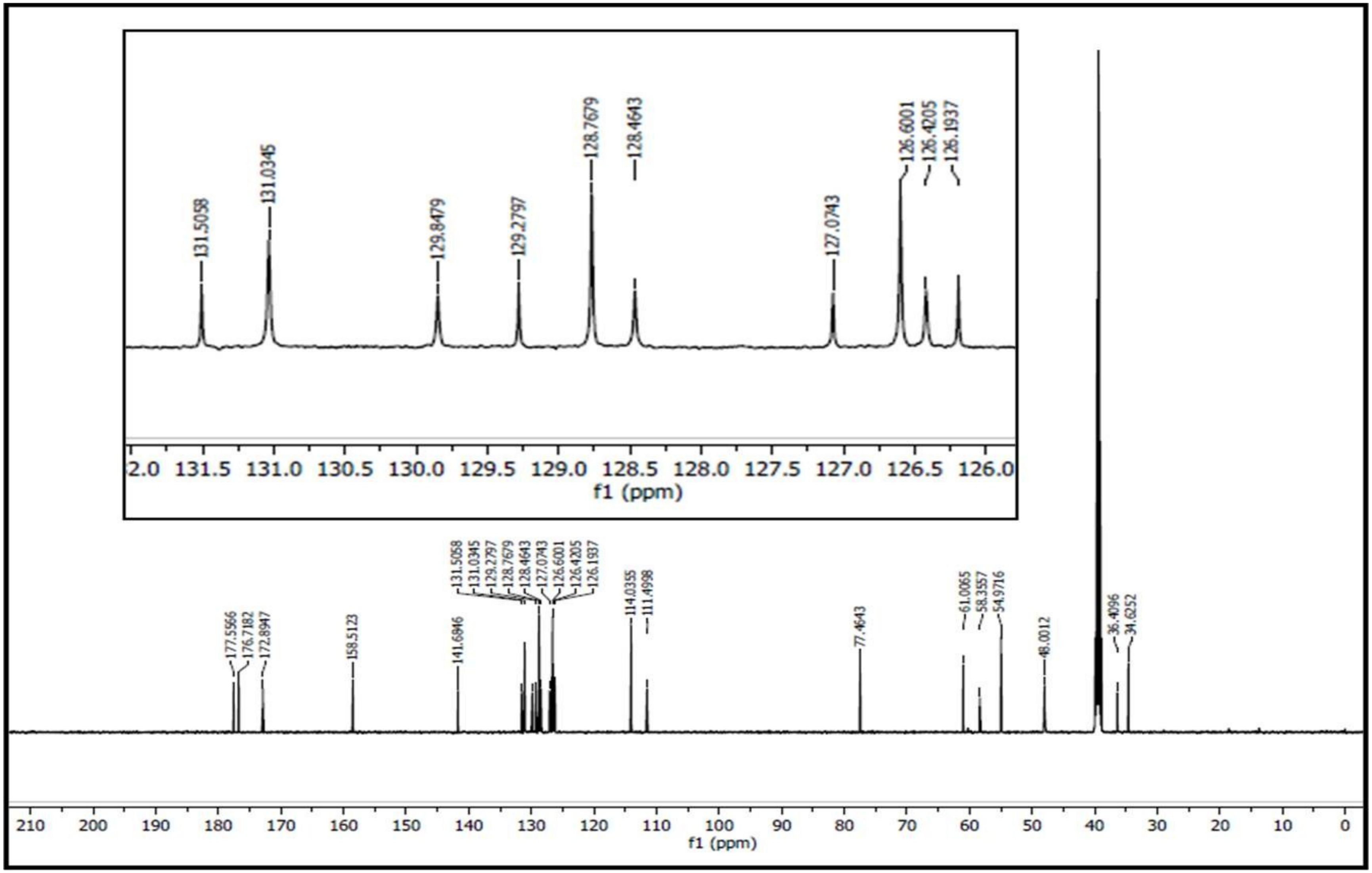

Fig. S19. ^{13}C-NMR spectrum of compound **8f** in DMSO-d_6.

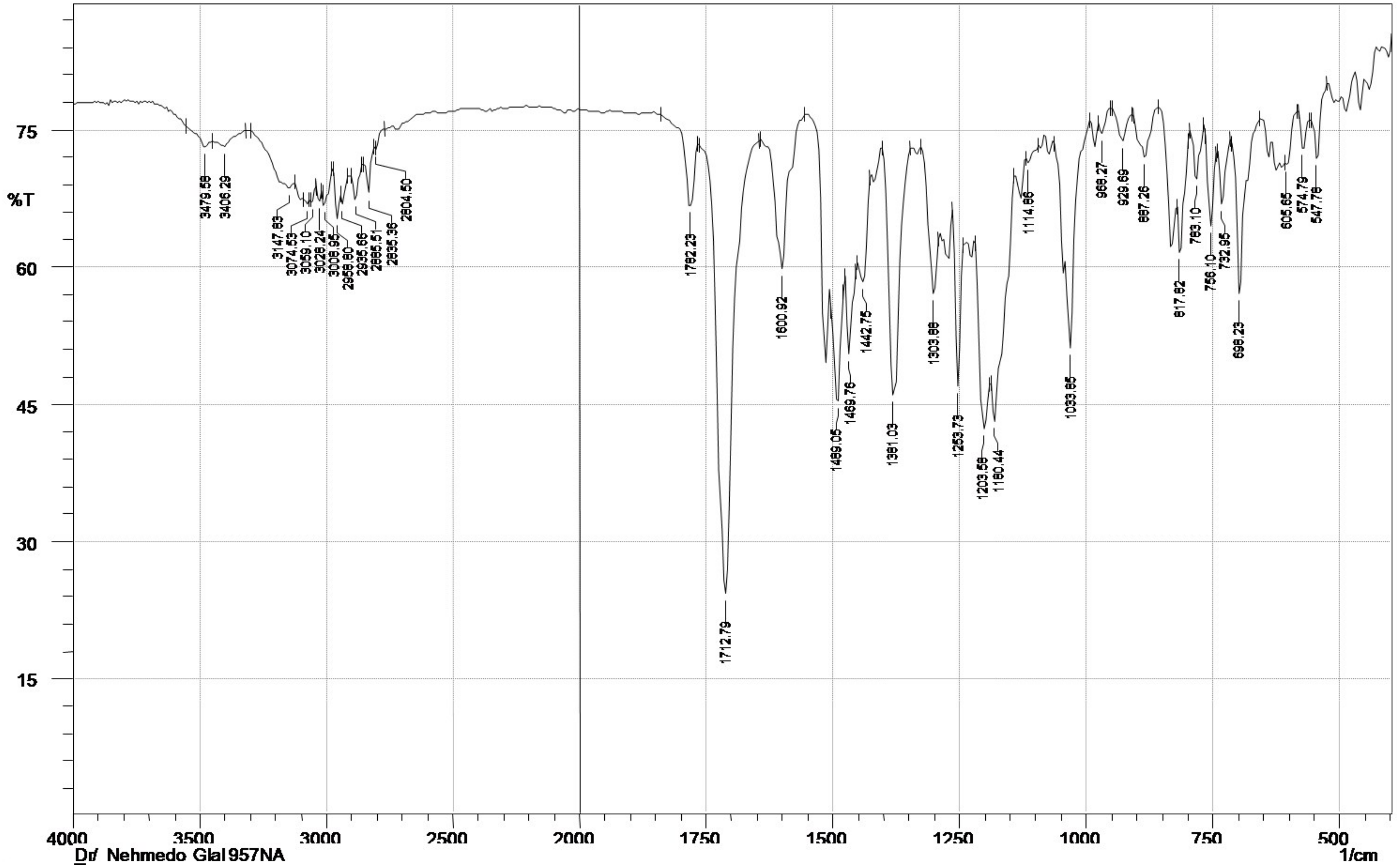

Fig. S20. IR spectrum of compound **8g** (KBr pellet).

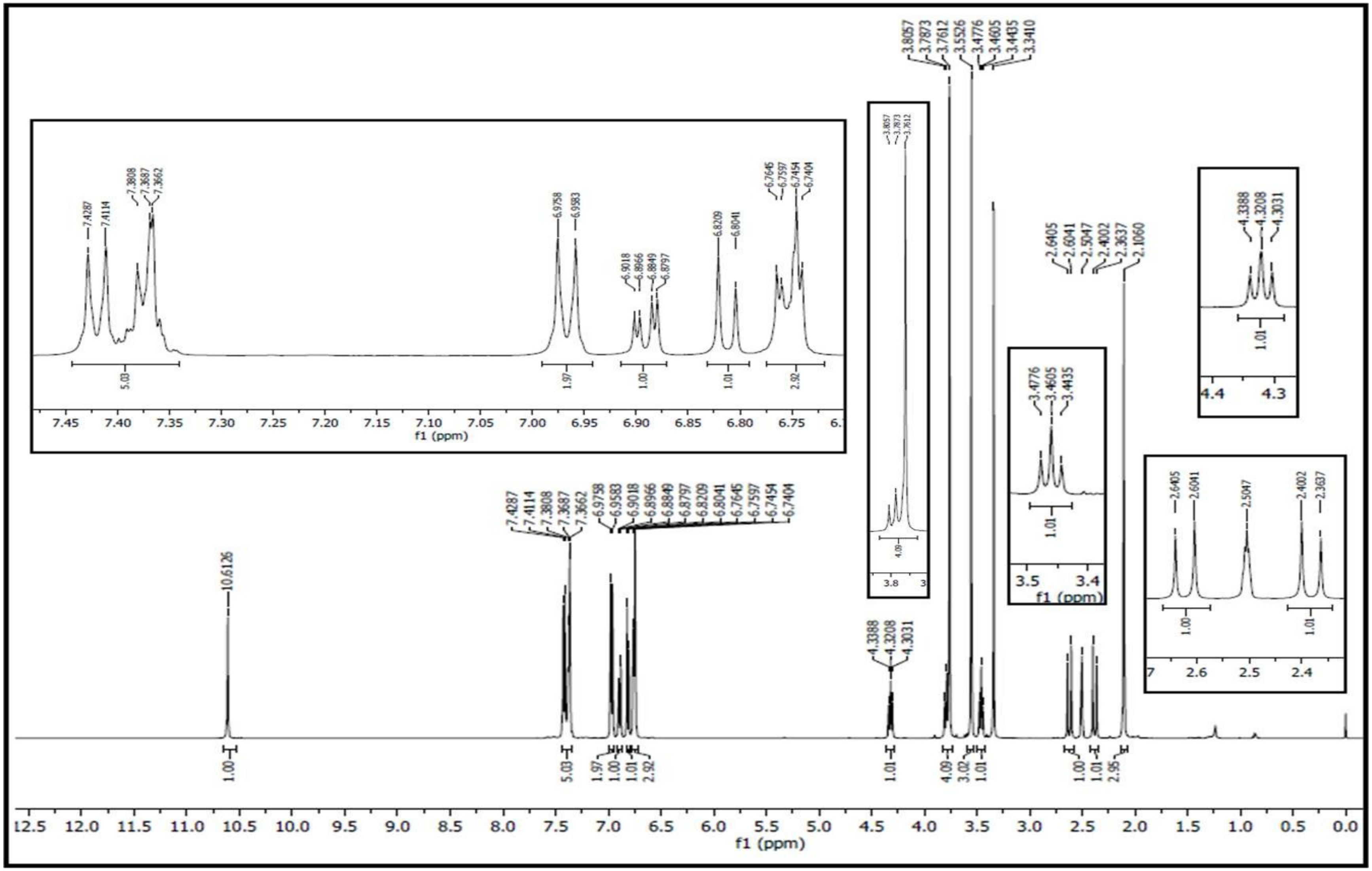

Fig. S21. ^{1}H-NMR spectrum of compound **8g** in DMSO-d_6.

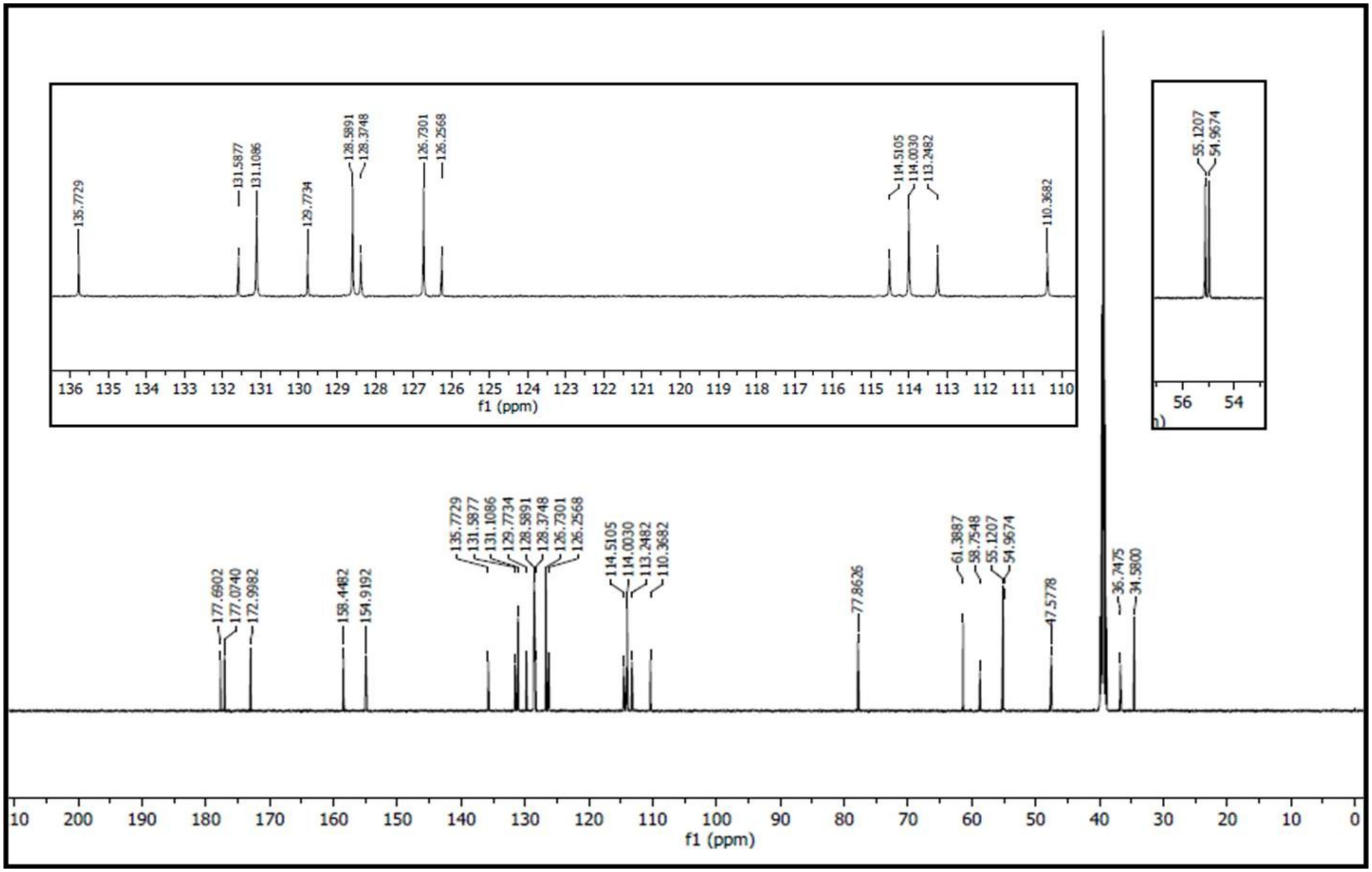

Fig. S22. ^{13}C-NMR spectrum of compound **8g** in DMSO-d_6.

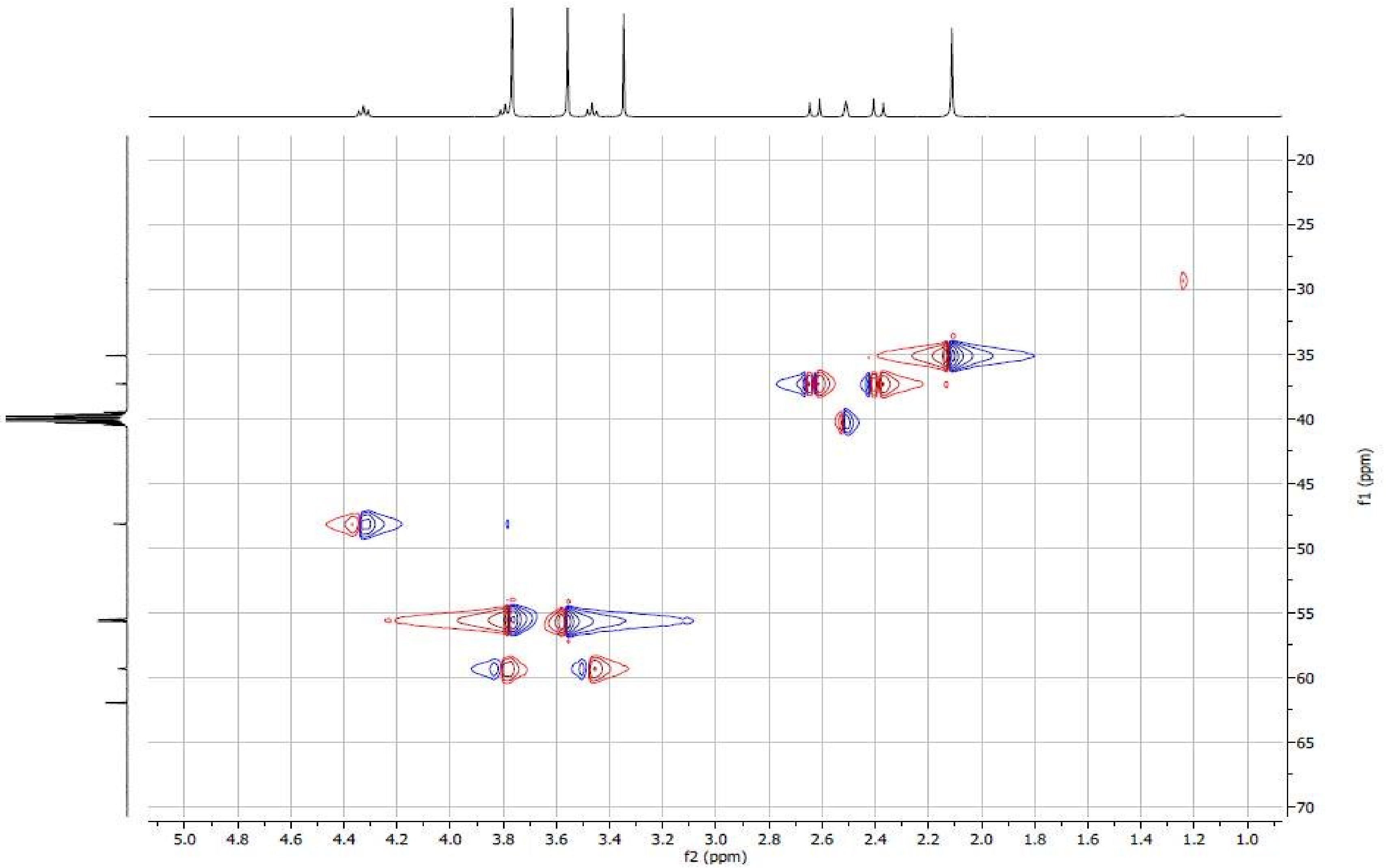

Fig. S23. HSQC spectrum of compound **8g** in DMSO-d_6.

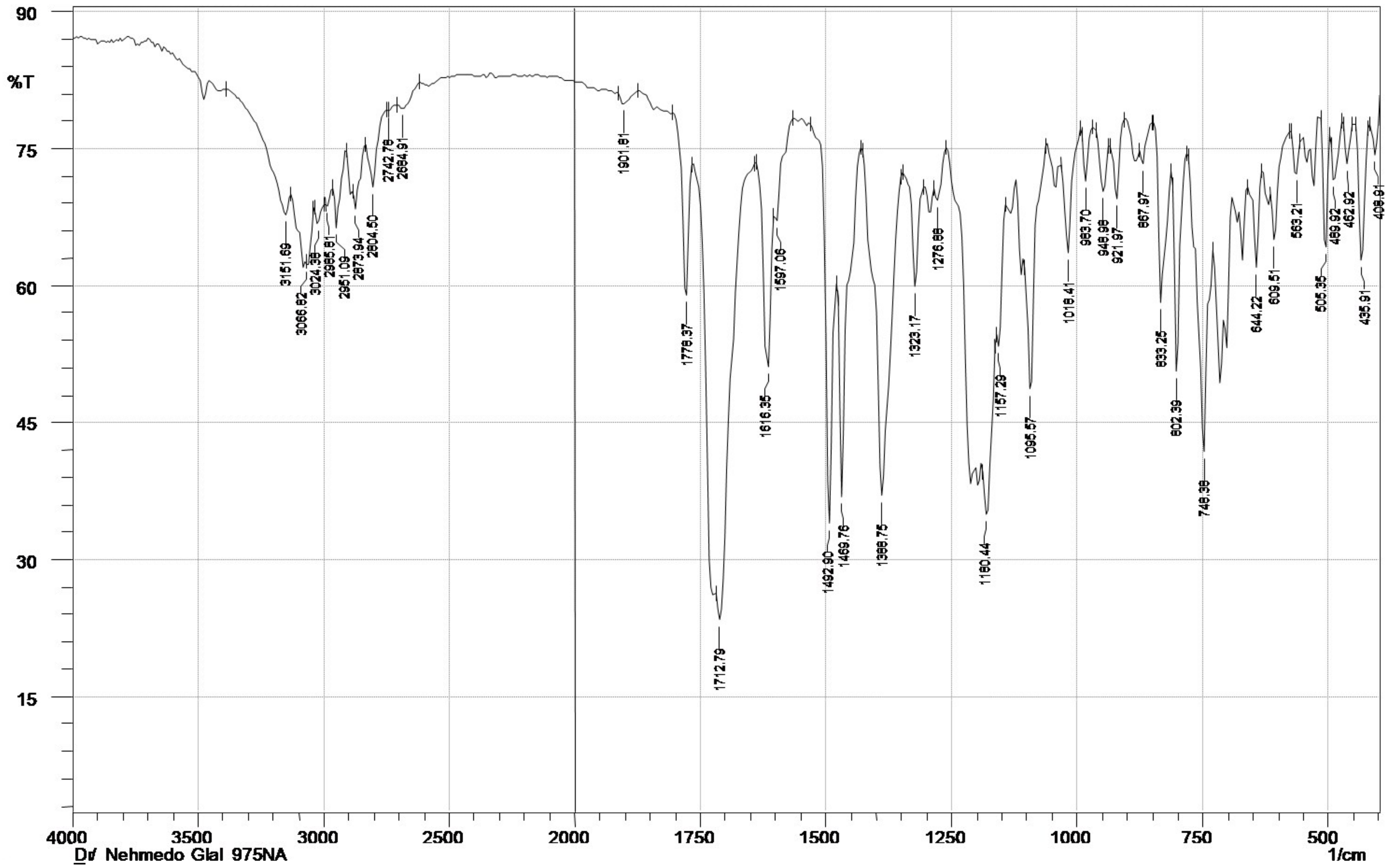

Fig. S24. IR spectrum of compound **8h** (KBr pellet).

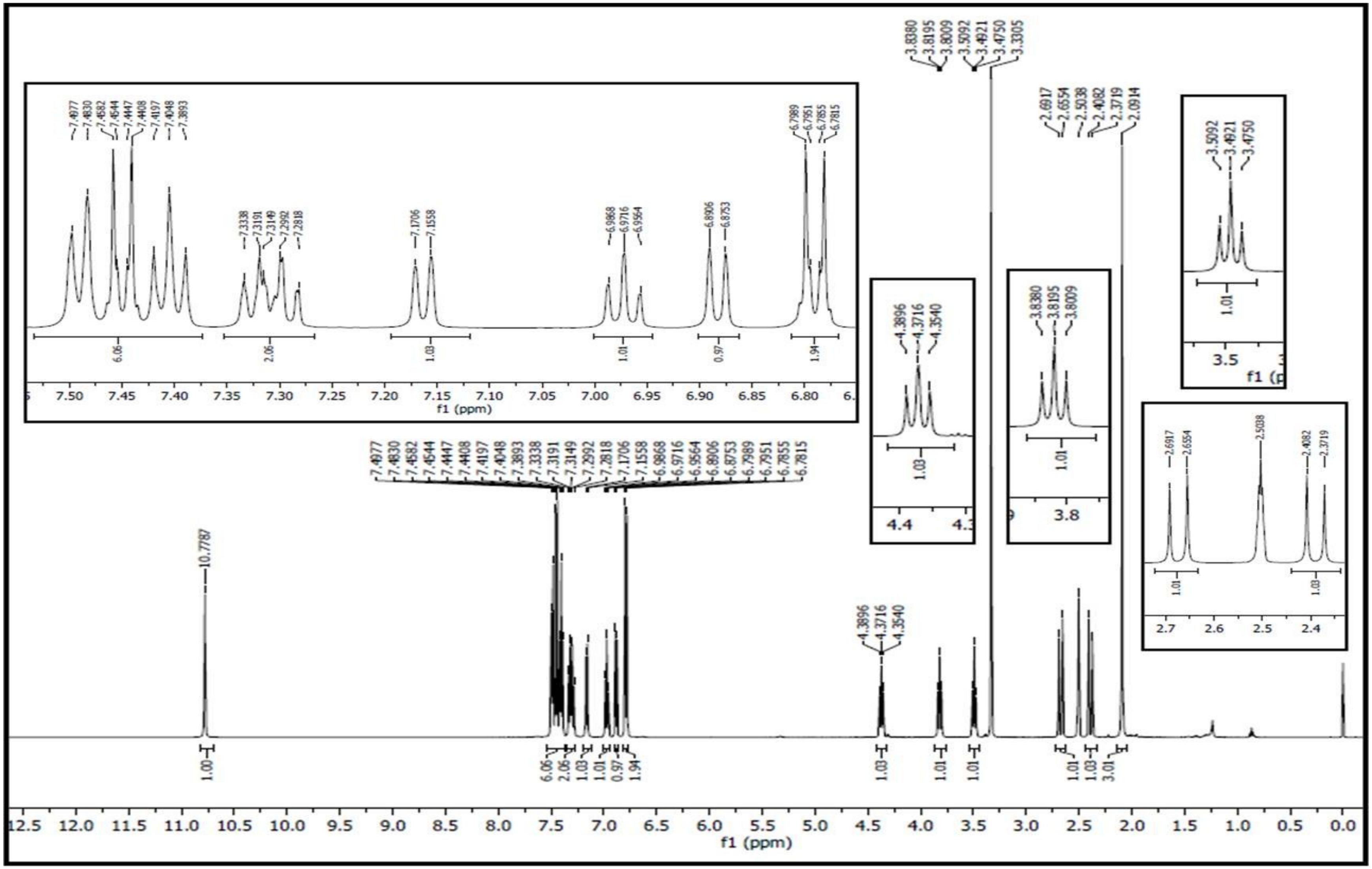

Fig. S25. ^{1}H-NMR spectrum of compound **8h** in DMSO-d_6.

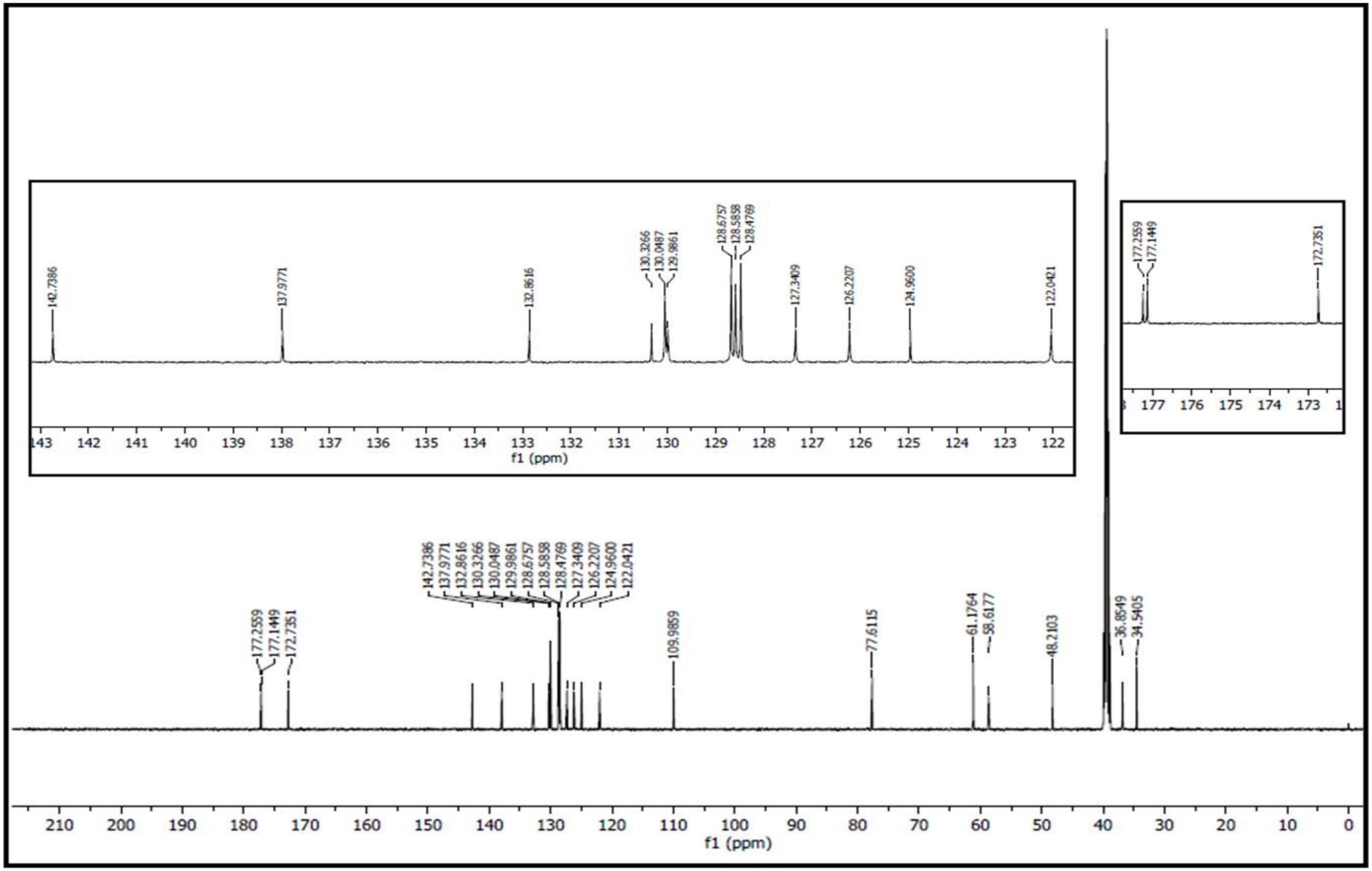

Fig. S26. ^{13}C-NMR spectrum of compound **8h** in DMSO-d_6.

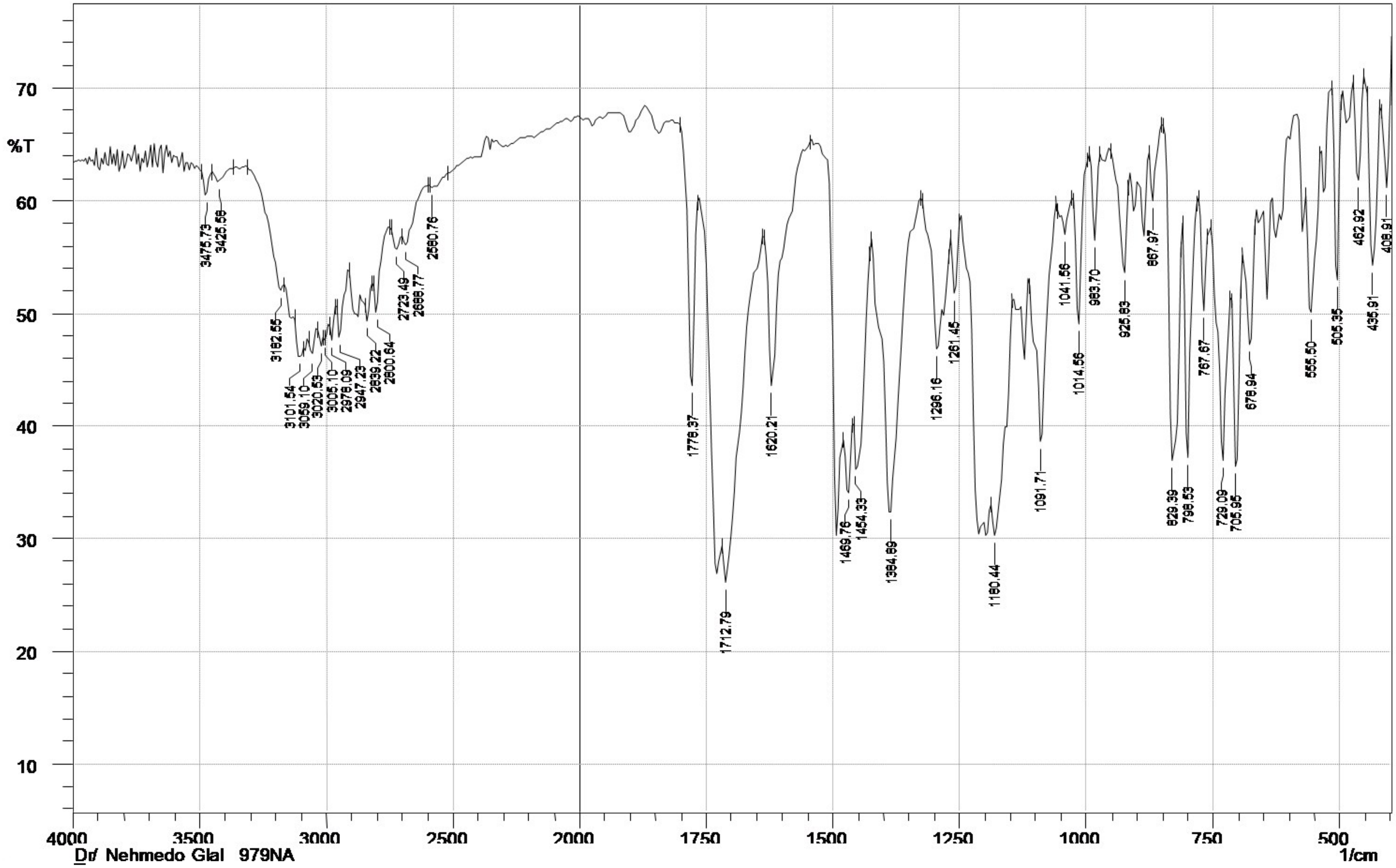

Fig. S27. IR spectrum of compound **8i** (KBr pellet).

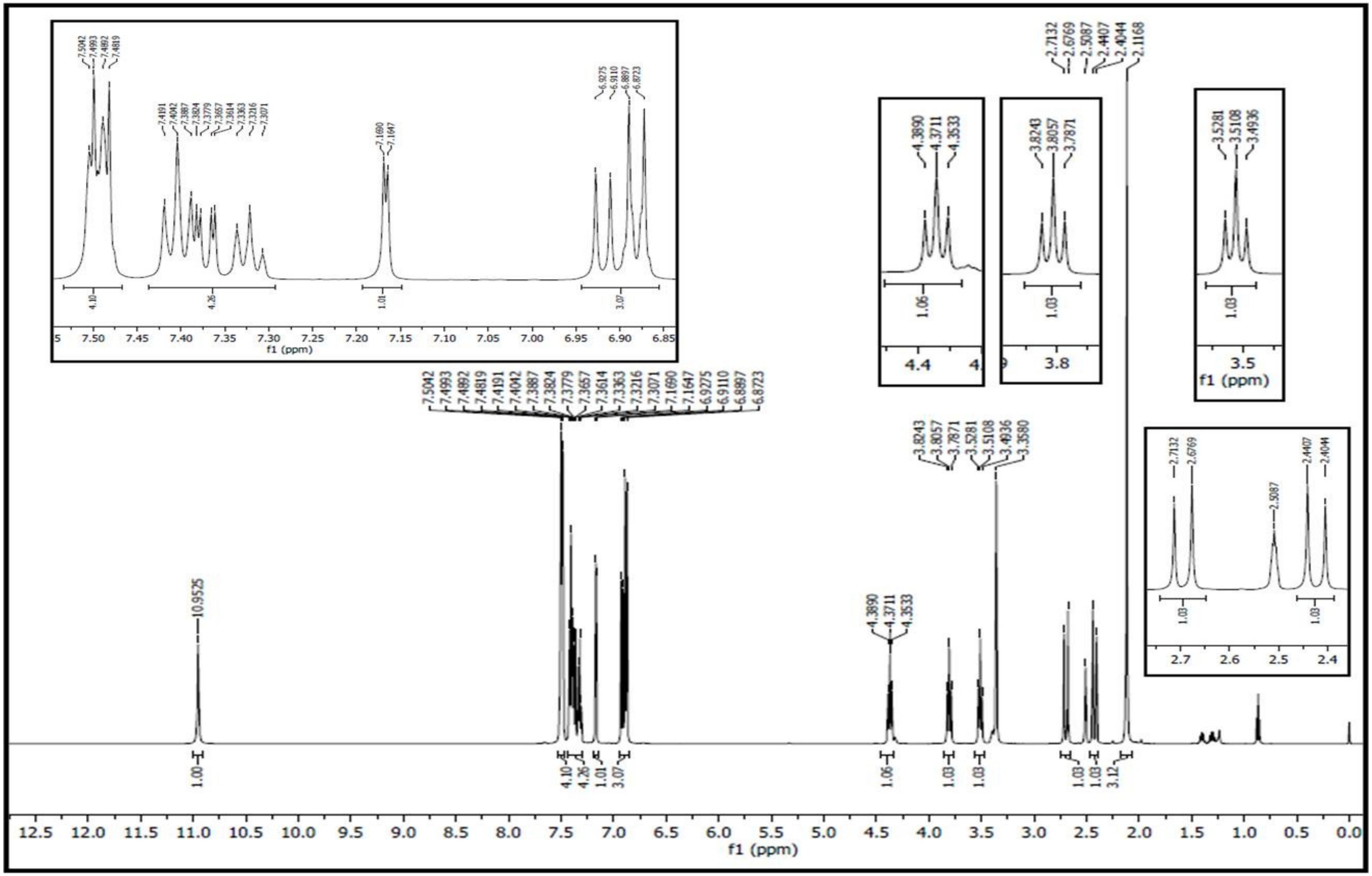

Fig. S28. ^{1}H-NMR spectrum of compound **8i** in DMSO-d_6.

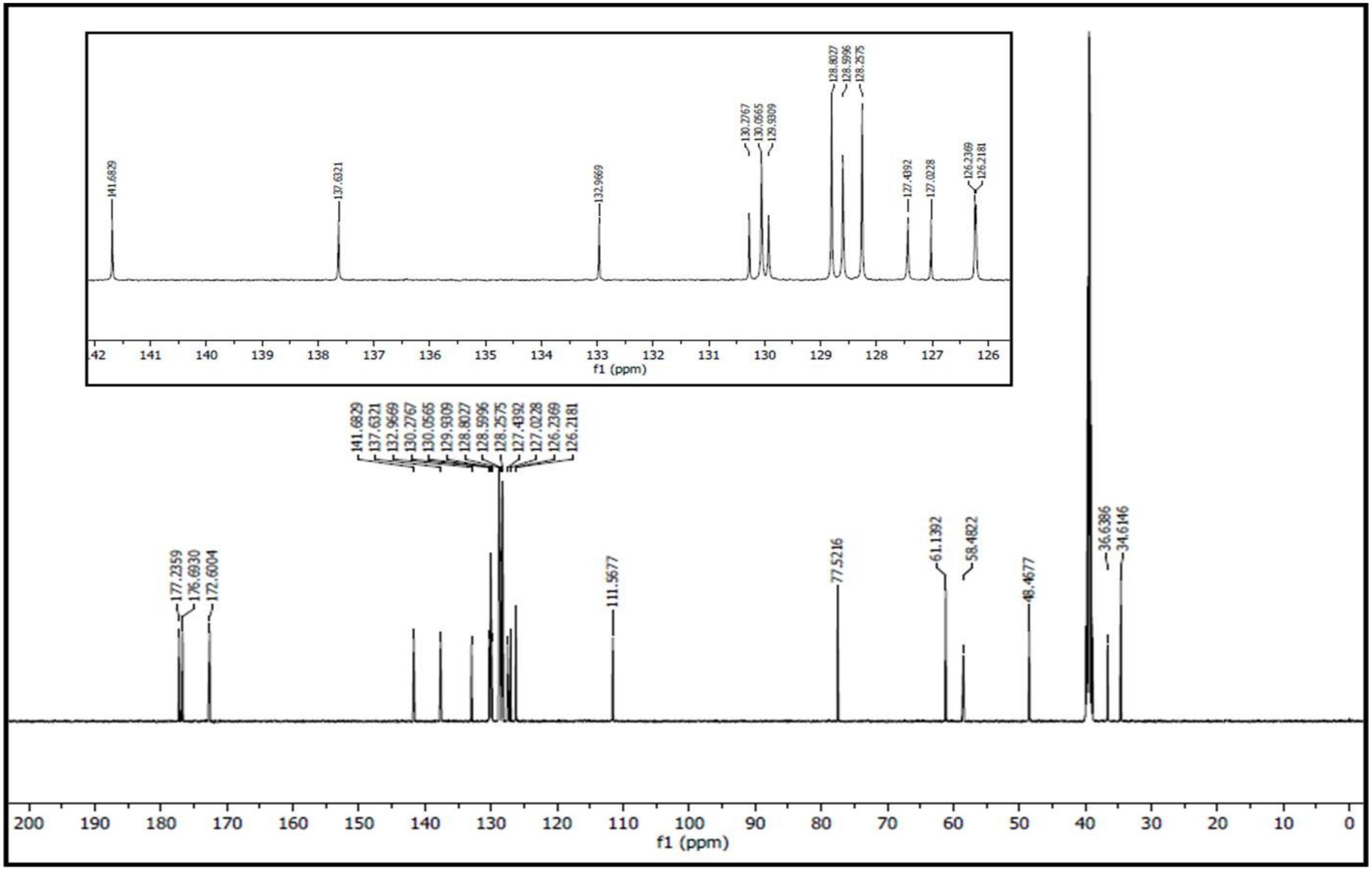

Fig. S29. ^{13}C-NMR spectrum of compound **8i** in DMSO-d_6.

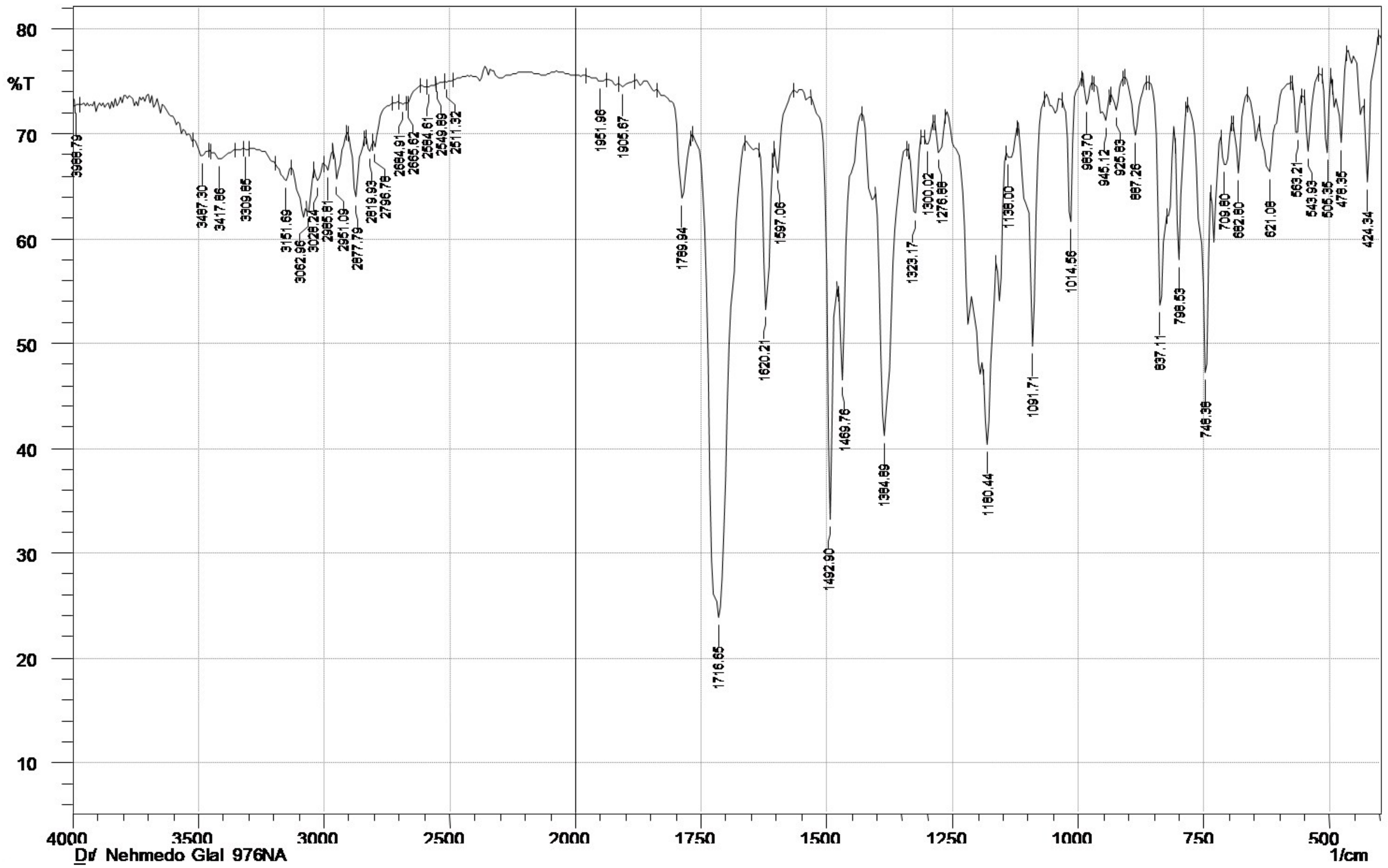

Fig. S30. IR spectrum of compound **8j** (KBr pellet).

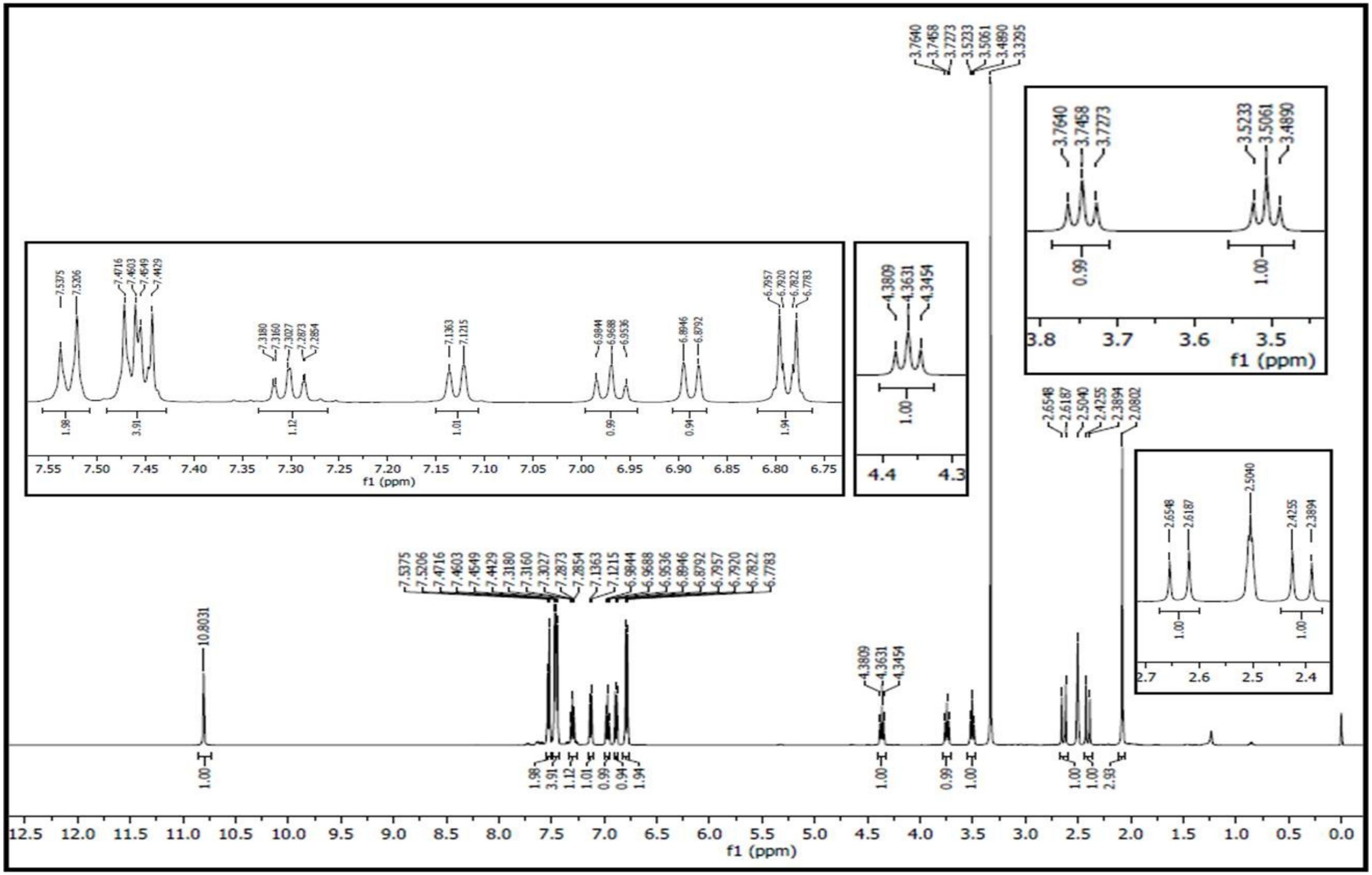

Fig. S31. ^{1}H-NMR spectrum of compound **8j** in DMSO-d_6.

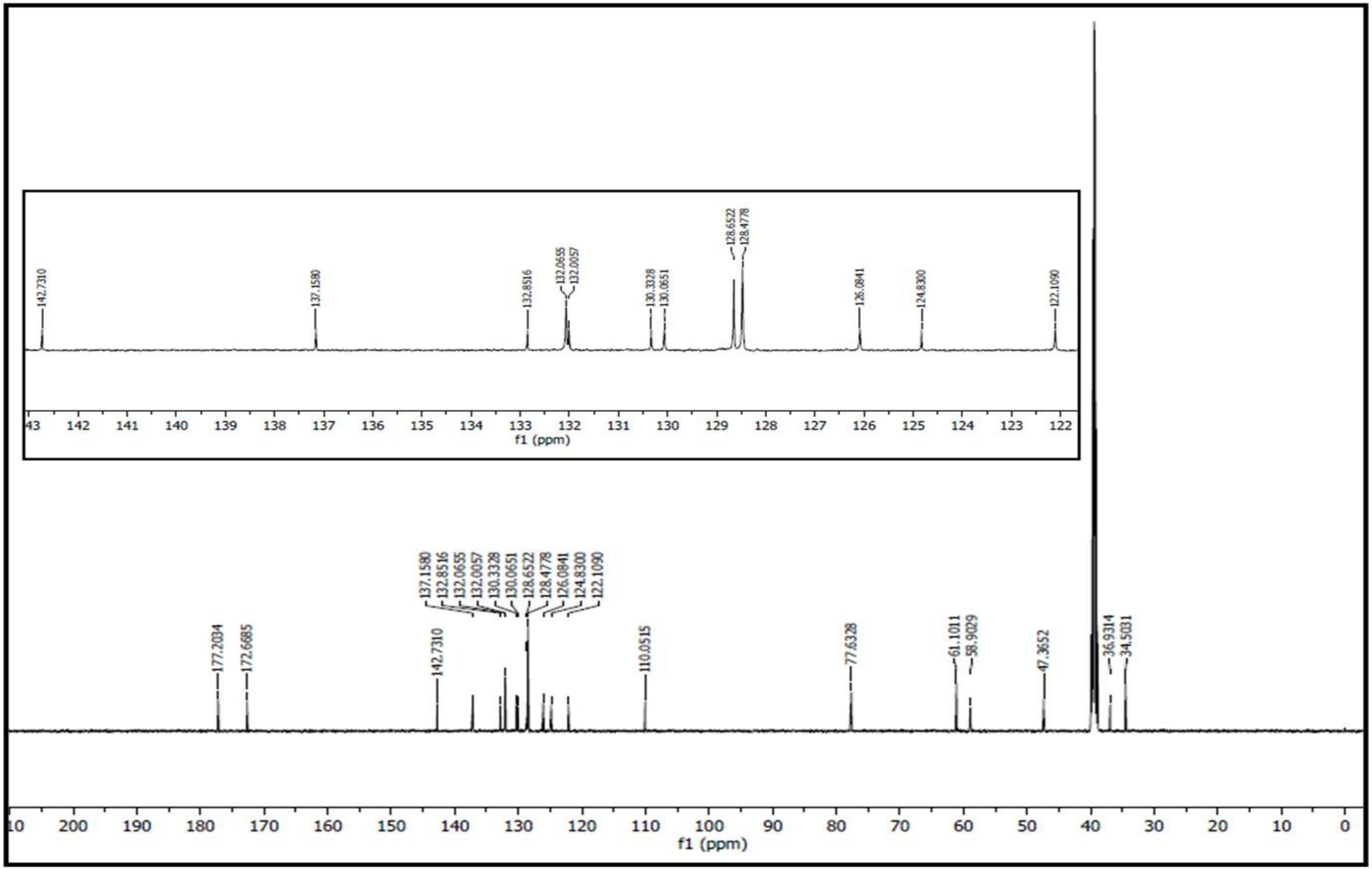

Fig. S32. ^{13}C-NMR spectrum of compound **8j** in DMSO-d_6.

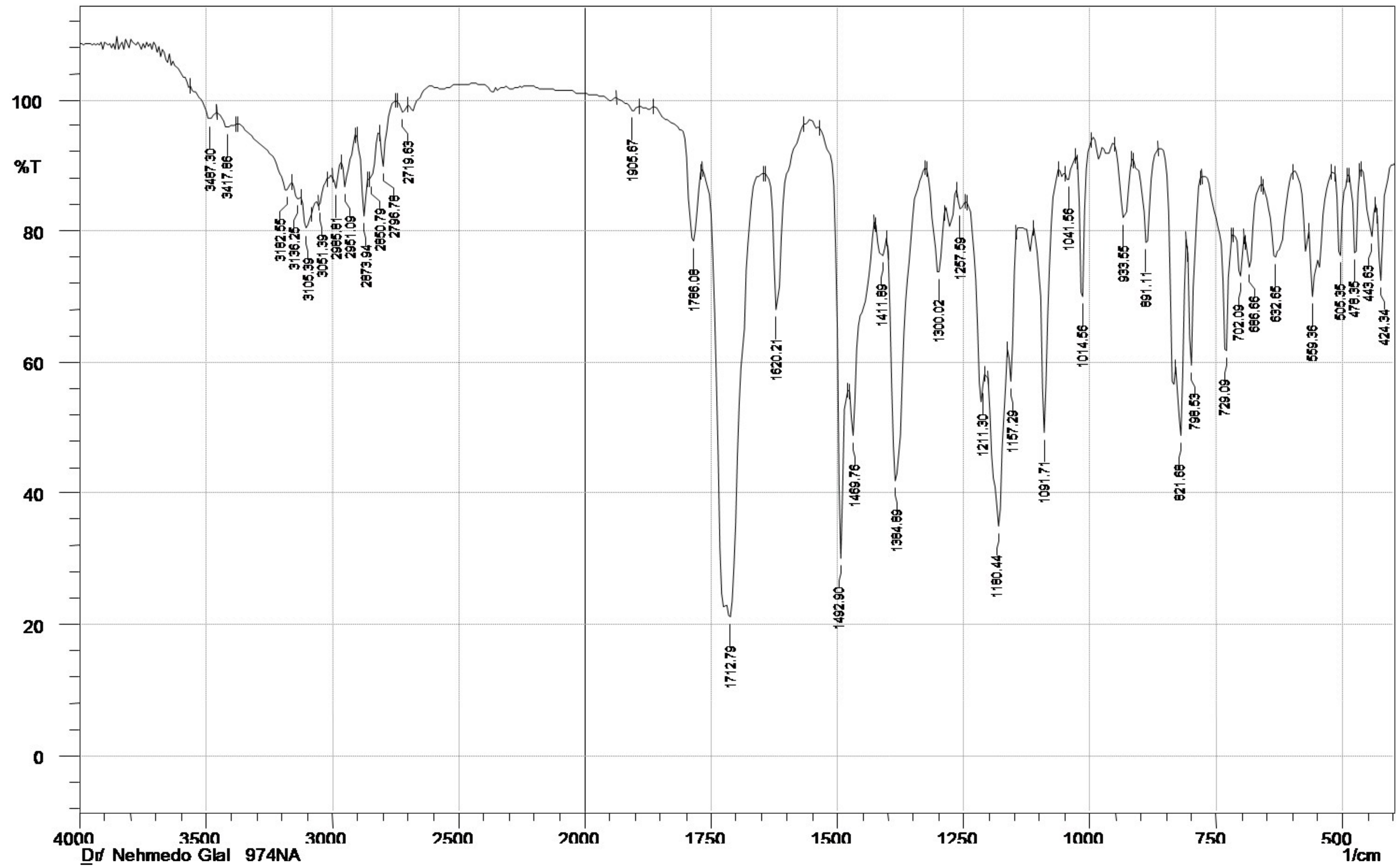

Fig. S33. IR spectrum of compound **8k** (KBr pellet).

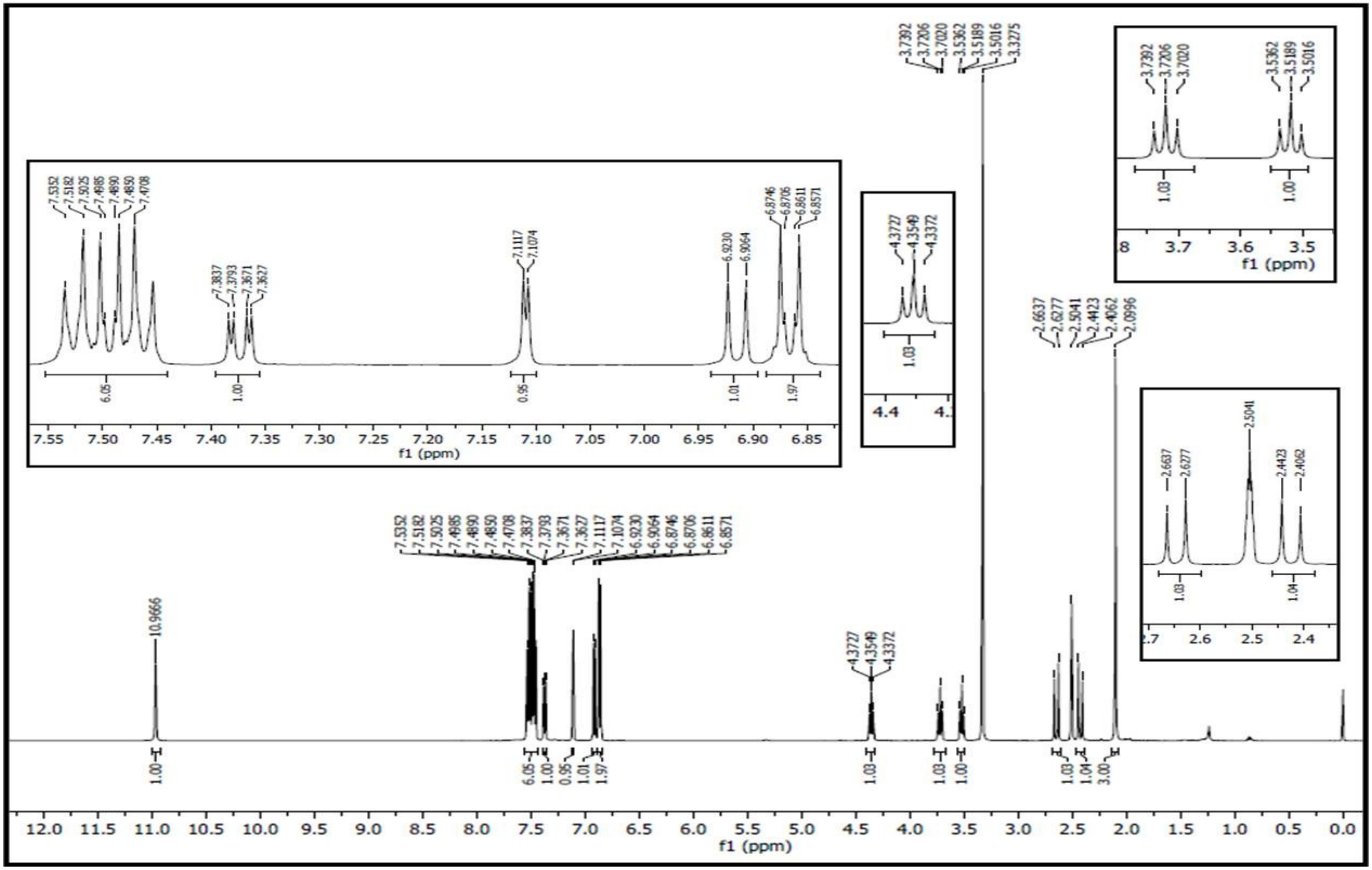

Fig. S34. ^{1}H-NMR spectrum of compound **8k** in DMSO-d_6.

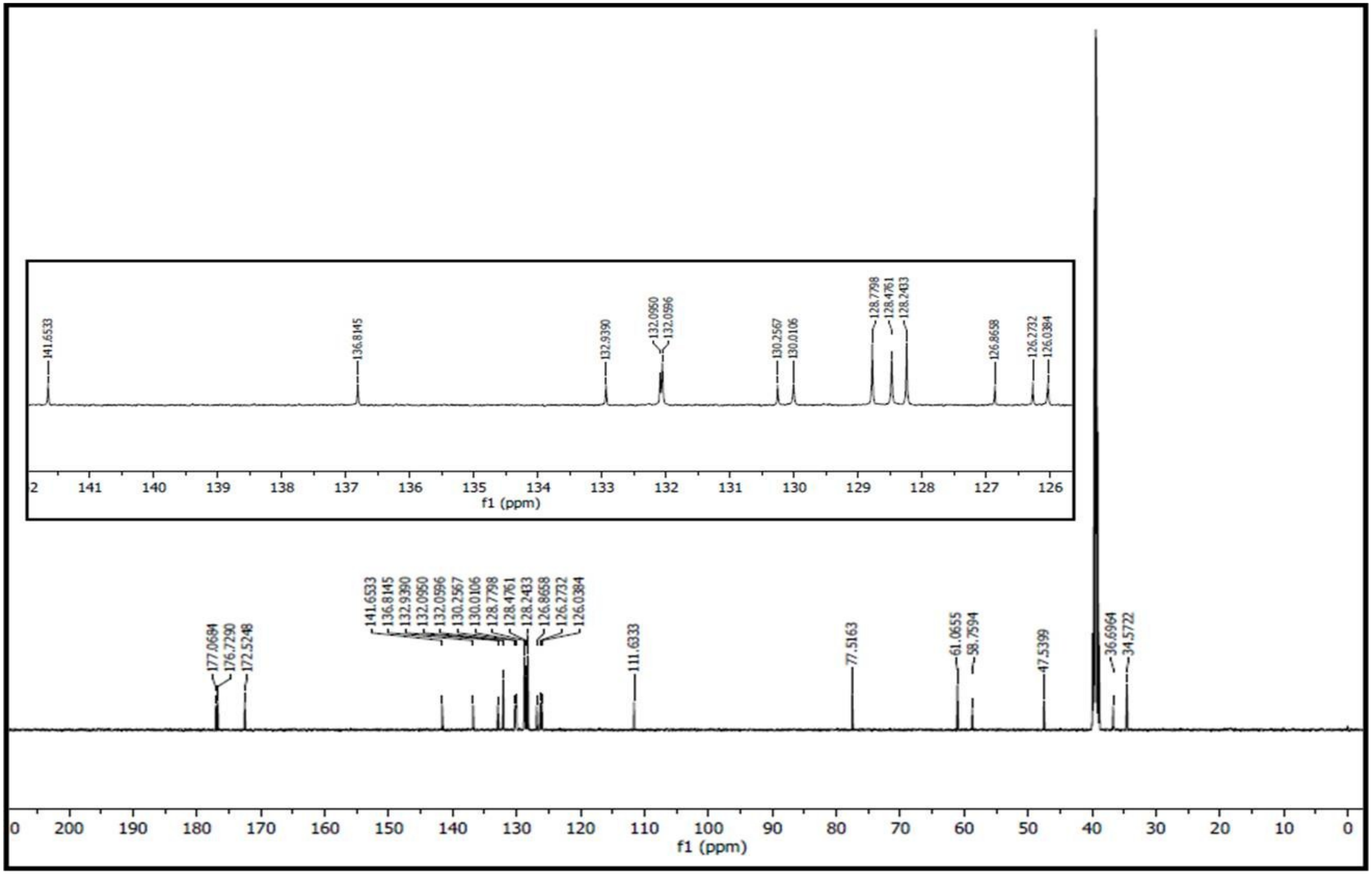

Fig. S35. ^{13}C-NMR spectrum of compound **8k** in DMSO-d_6.

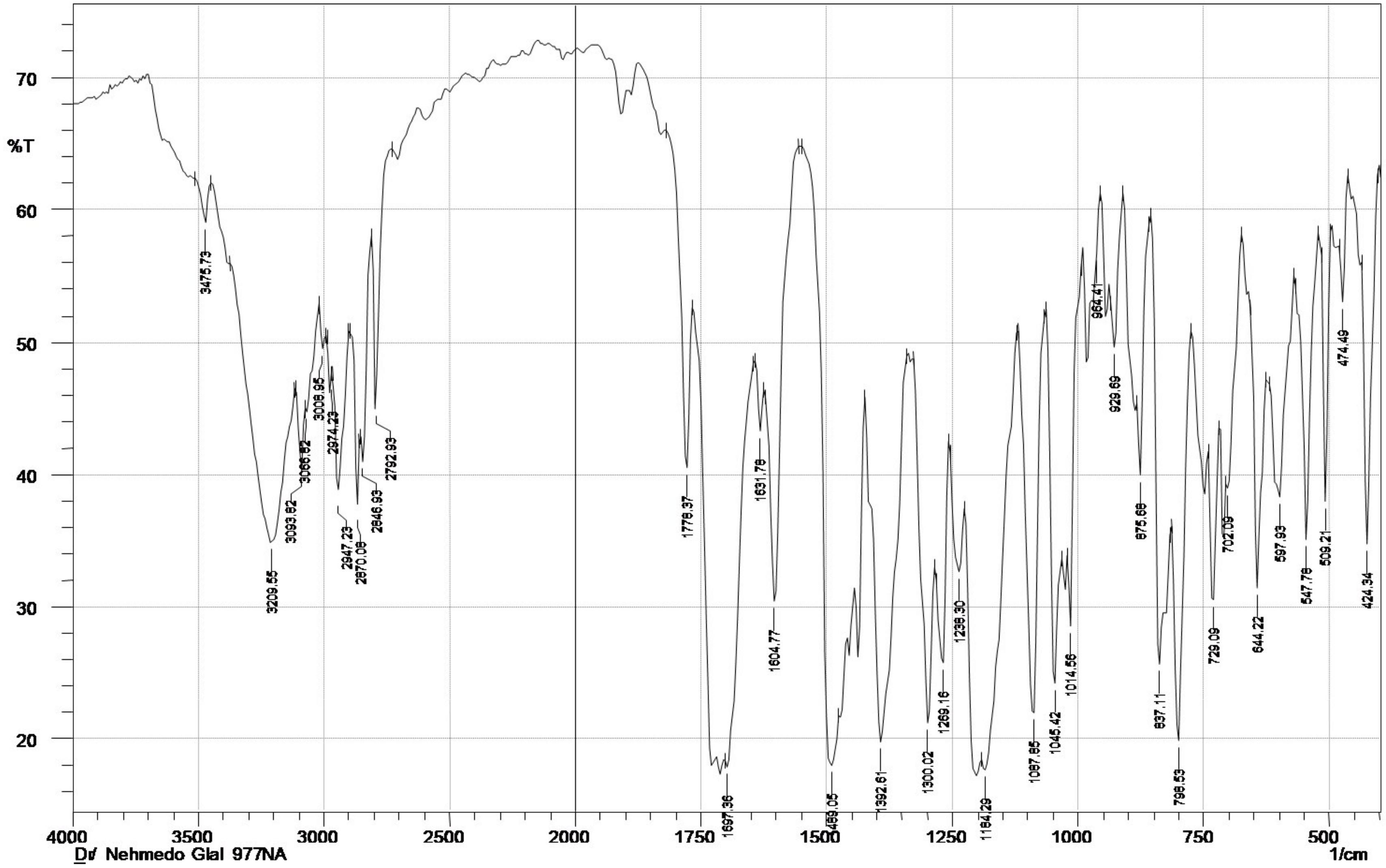

Fig. S36. IR spectrum of compound **8l** (KBr pellet).

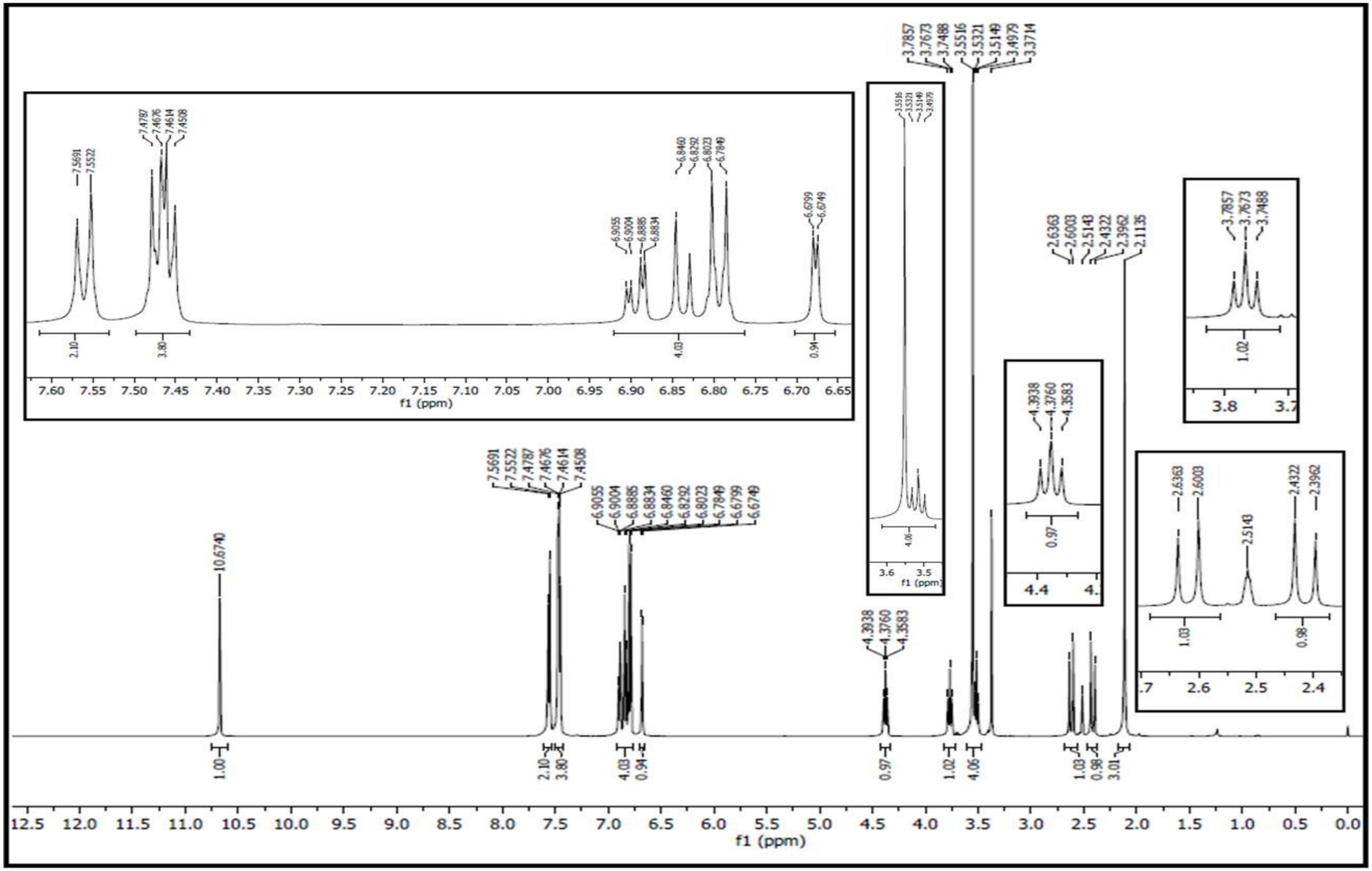

Fig. S37. ^{1}H-NMR spectrum of compound **8l** in DMSO-d_6.

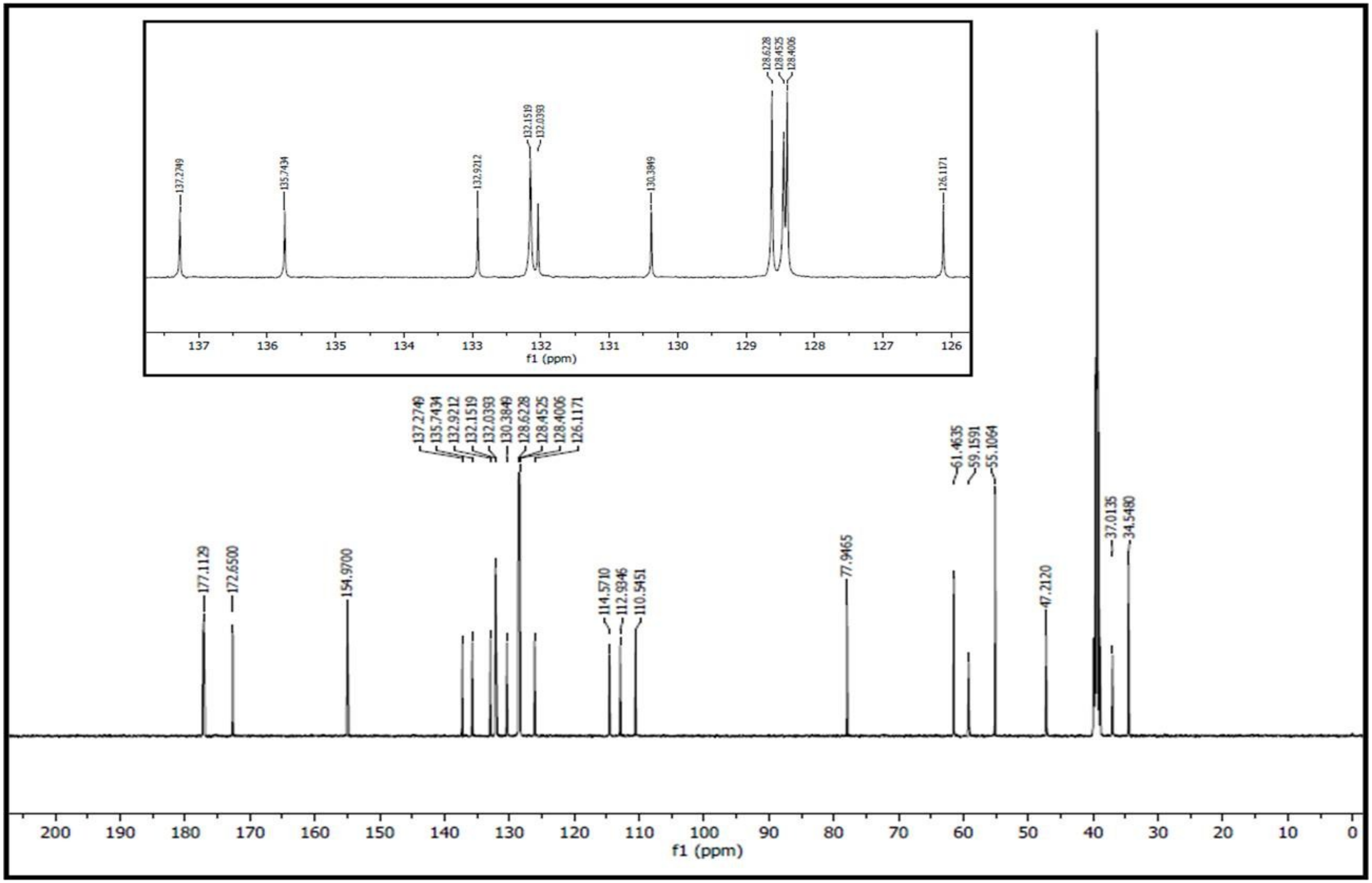

Fig. S38. ^{13}C-NMR spectrum of compound **8l** in DMSO-d_6.

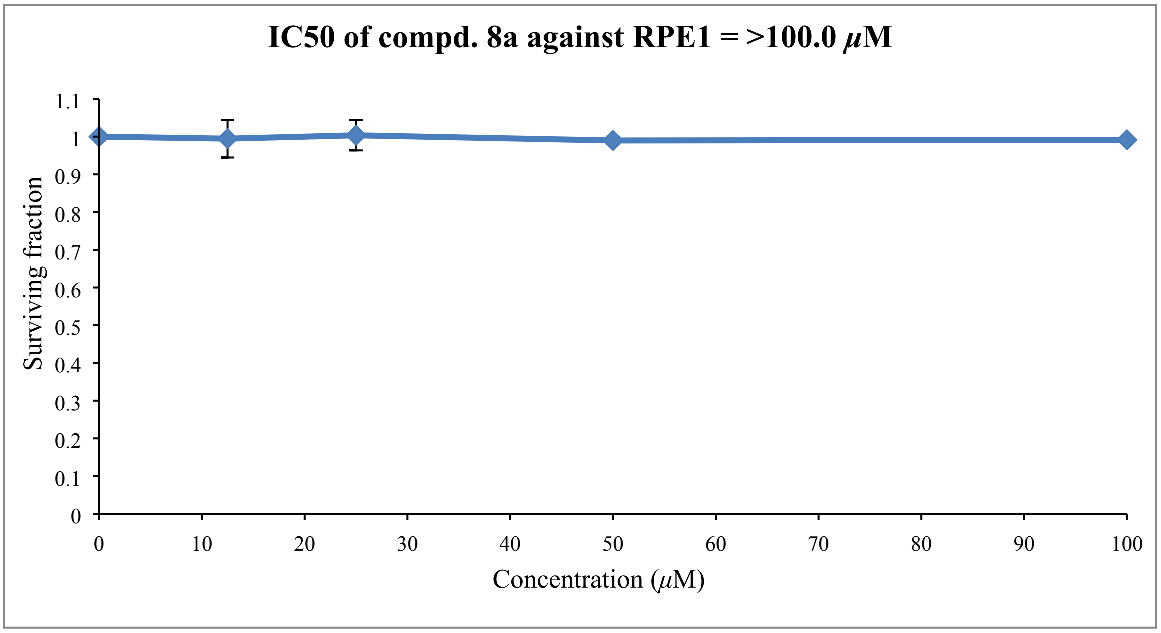
IC50 of compd. 8a against RPE1 = >100.0 μM
Surviving fraction
Concentration (μM)

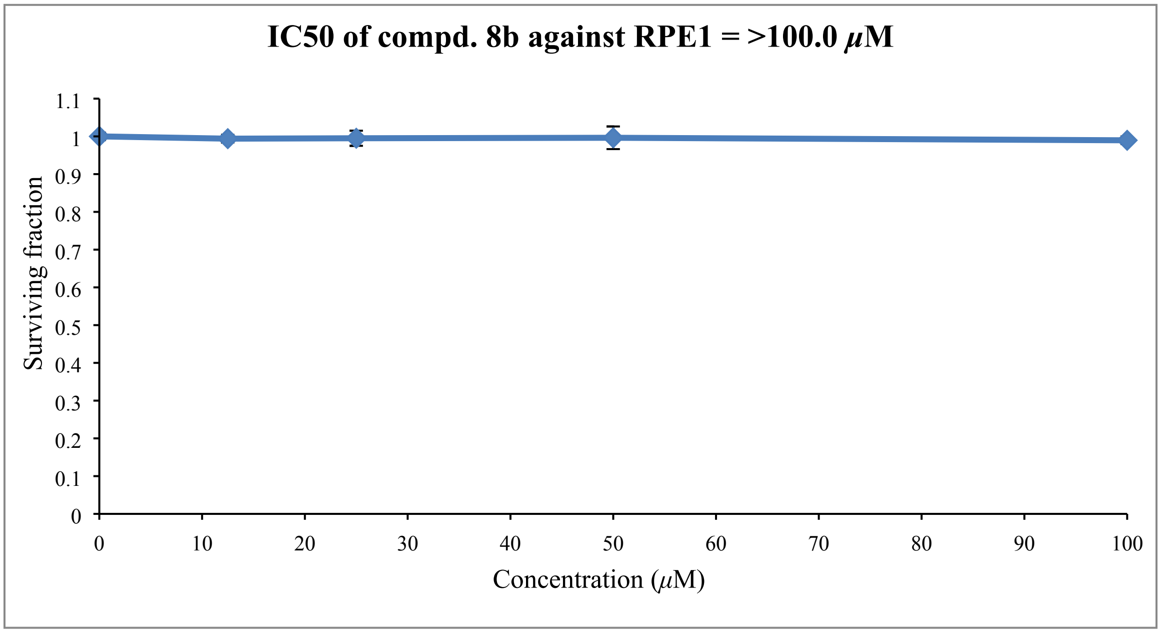
IC50 of compd. 8b against RPE1 = >100.0 μM
Surviving fraction
Concentration (μM)

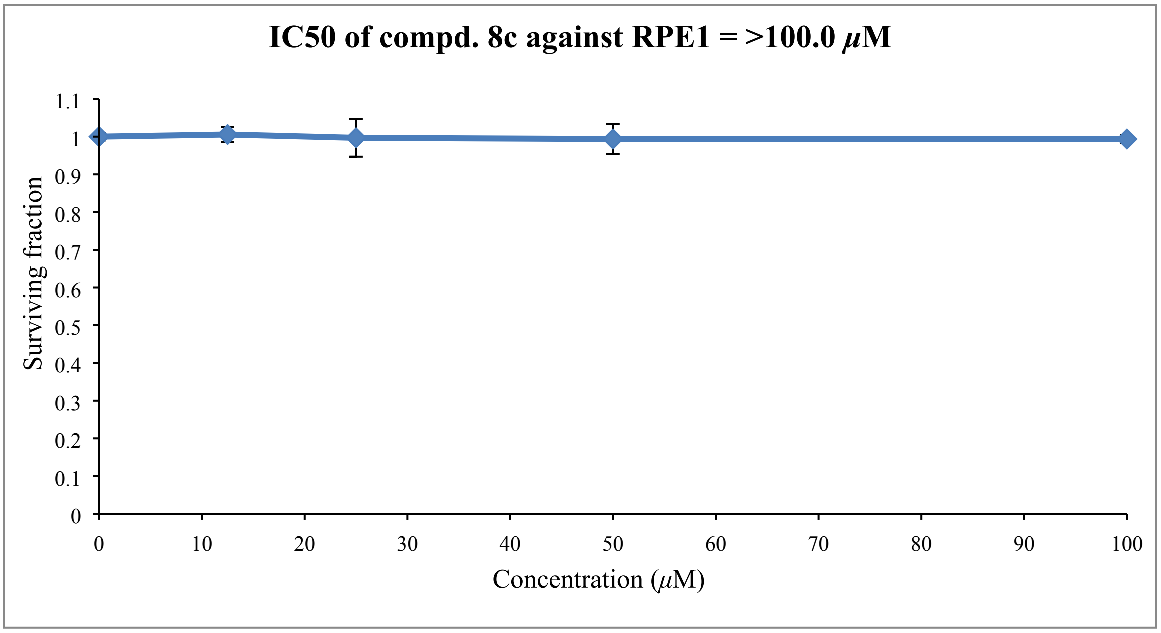
IC50 of compd. 8c against RPE1 = >100.0 μM
Surviving fraction
1.1
1
0.9
0.8
0.7
0.6
0.5
0.4
0.3
0.2
0.1
0
0
10
20
30
40
50
60
70
80
90
100
Concentration (μM)

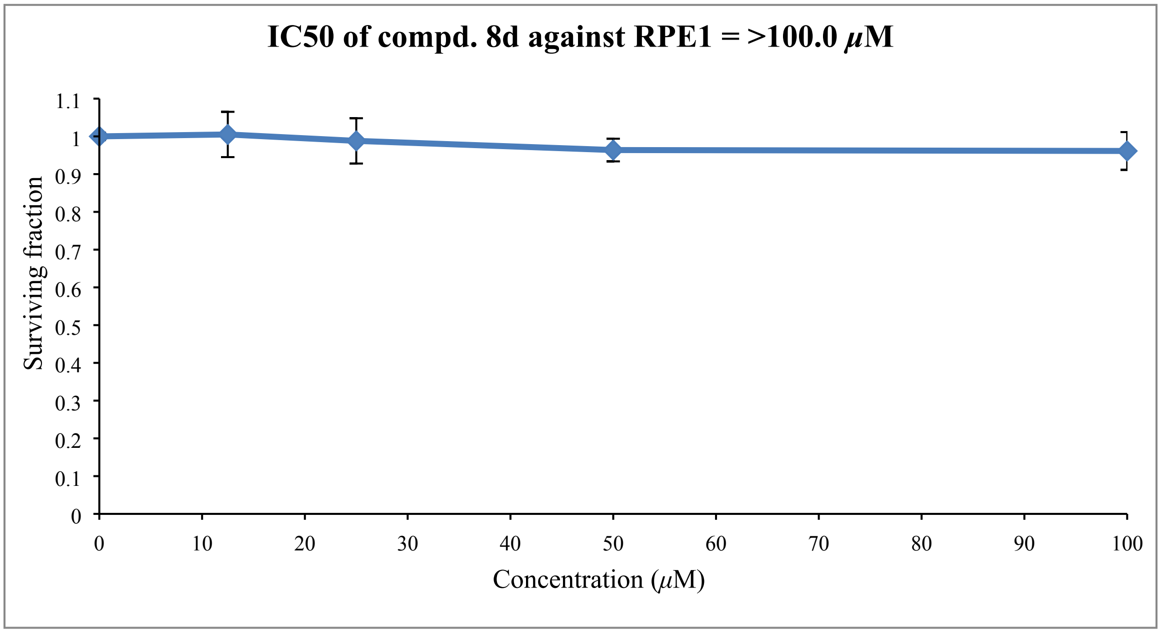
IC50 of compd. 8d against RPE1 = >100.0 μM
Surviving fraction
1.1
1
0.9
0.8
0.7
0.6
0.5
0.4
0.3
0.2
0.1
0
0
10
20
30
40
50
60
70
80
90
100
Concentration (μM)

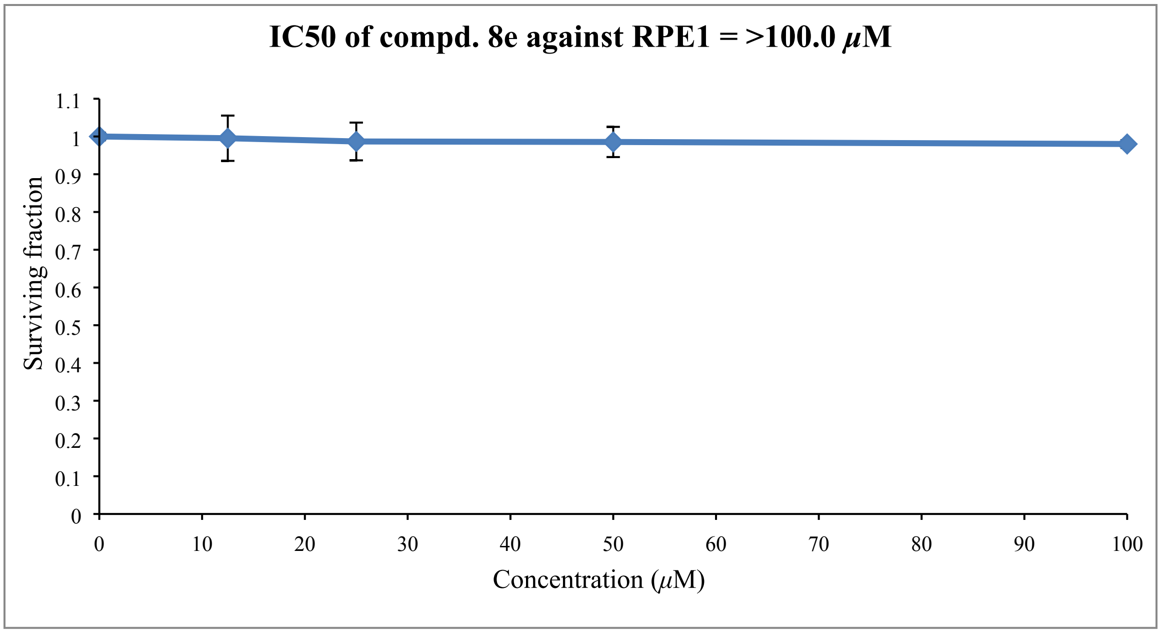
IC50 of compd. 8e against RPE1 = >100.0 μM
Surviving fraction
1.1
1
0.9
0.8
0.7
0.6
0.5
0.4
0.3
0.2
0.1
0
0
10
20
30
40
50
60
70
80
90
100
Concentration (μM)

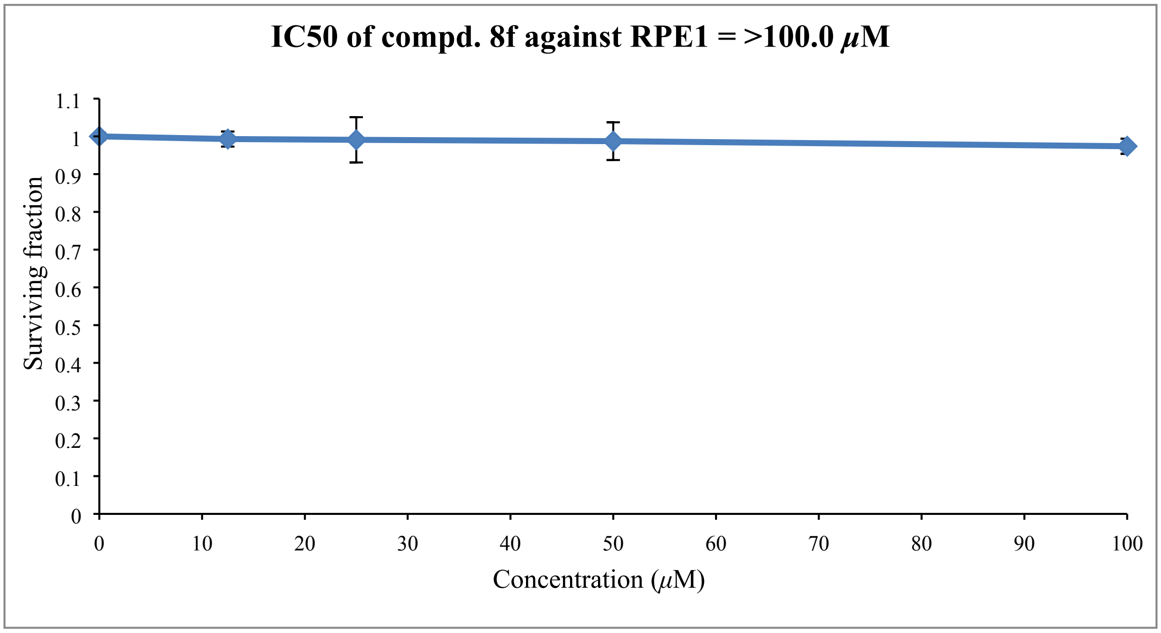
IC50 of compd. 8f against RPE1 = >100.0 μM
Surviving fraction
1.1
1
0.9
0.8
0.7
0.6
0.5
0.4
0.3
0.2
0.1
0
0
10
20
30
40
50
60
70
80
90
100
Concentration (μM)

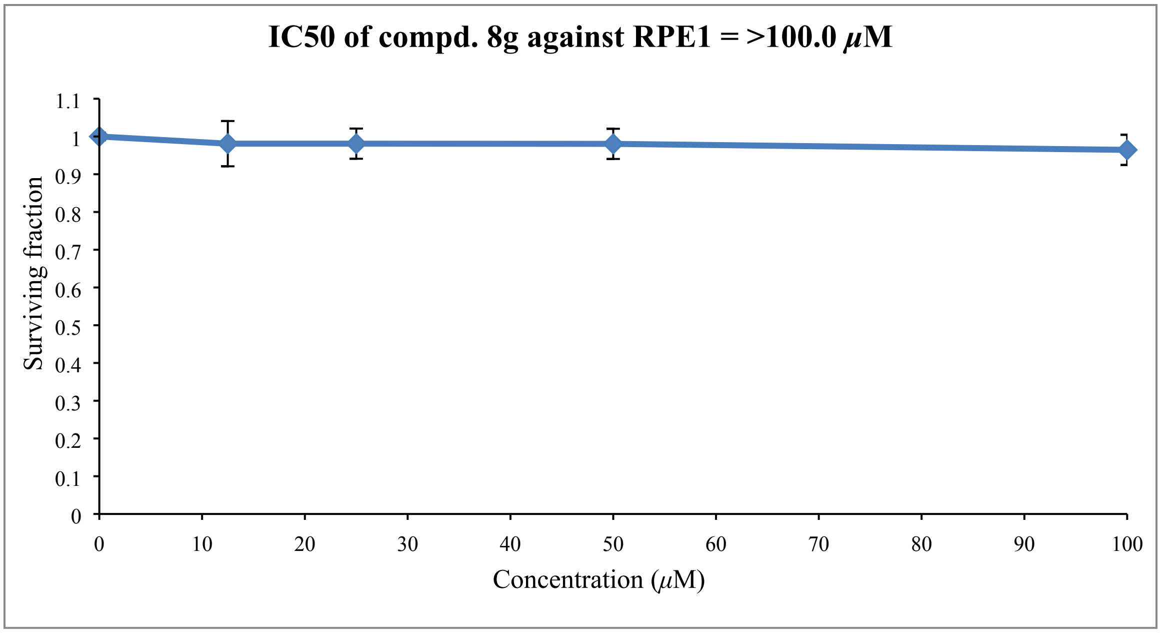

IC50 of compd. 8g against RPE1 = >100.0 μM
Surviving fraction
1.1
1
0.9
0.8
0.7
0.6
0.5
0.4
0.3
0.2
0.1
0
0
10
20
30
40
50
60
70
80
90
100
Concentration (μM)

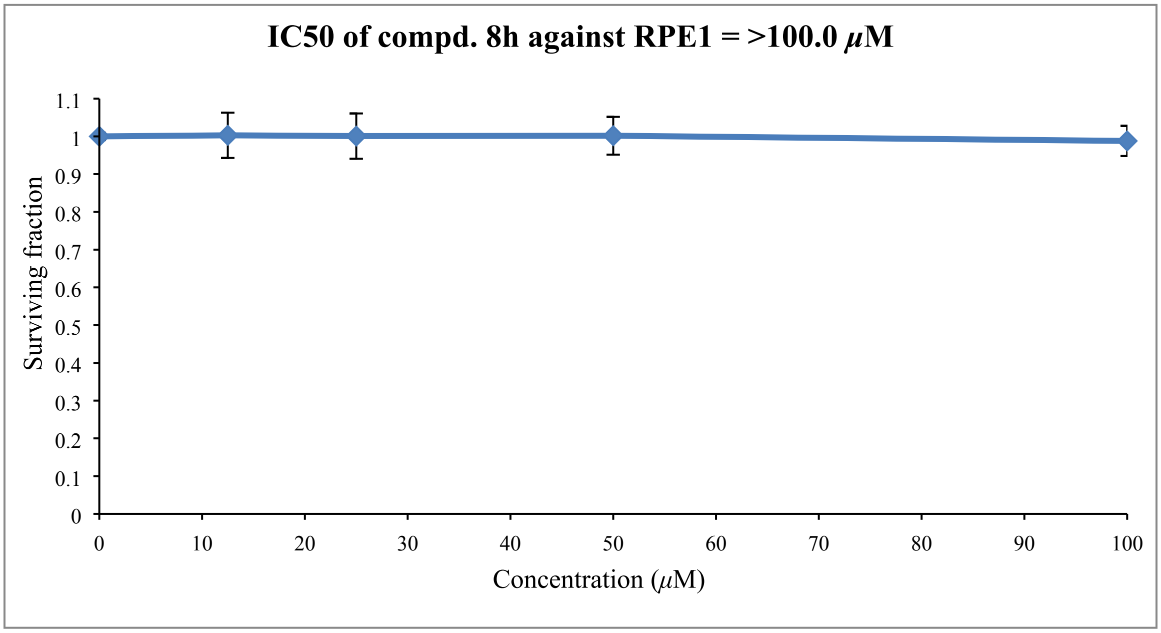

IC50 of compd. 8h against RPE1 = >100.0 μM
Surviving fraction
1.1
1
0.9
0.8
0.7
0.6
0.5
0.4
0.3
0.2
0.1
0
0
10
20
30
40
50
60
70
80
90
100
Concentration (μM)

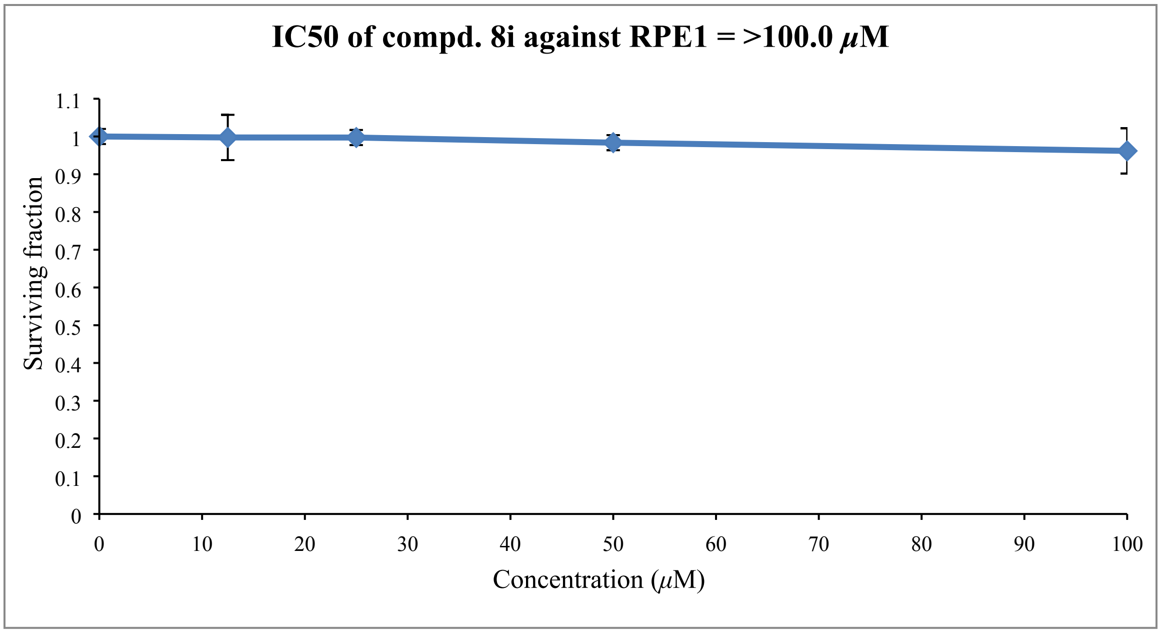
IC50 of compd. 8i against RPE1 = >100.0 μM
Surviving fraction
1.1
1
0.9
0.8
0.7
0.6
0.5
0.4
0.3
0.2
0.1
0
0
10
20
30
40
50
60
70
80
90
100
Concentration (μM)

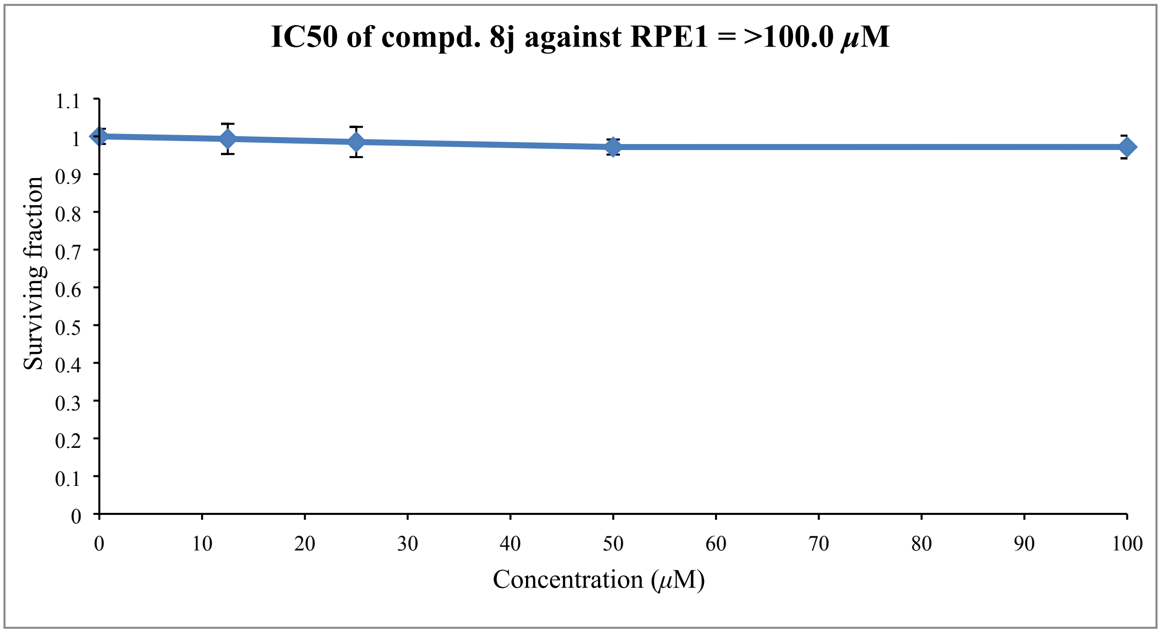
IC50 of compd. 8j against RPE1 = >100.0 μM
Surviving fraction
1.1
1
0.9
0.8
0.7
0.6
0.5
0.4
0.3
0.2
0.1
0
0
10
20
30
40
50
60
70
80
90
100
Concentration (μM)

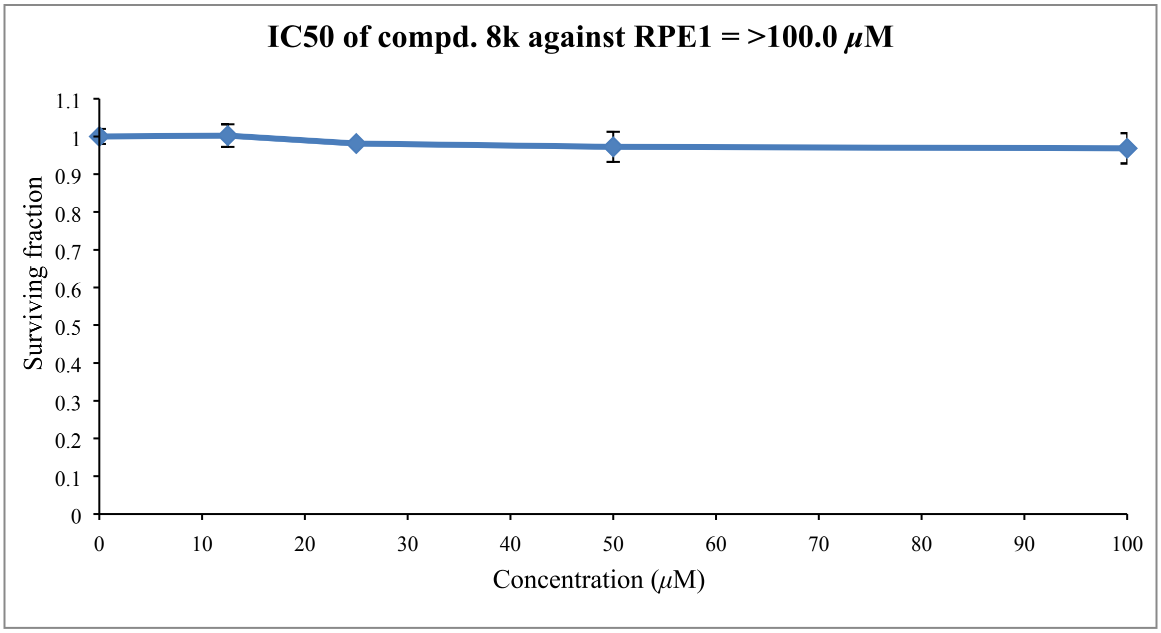

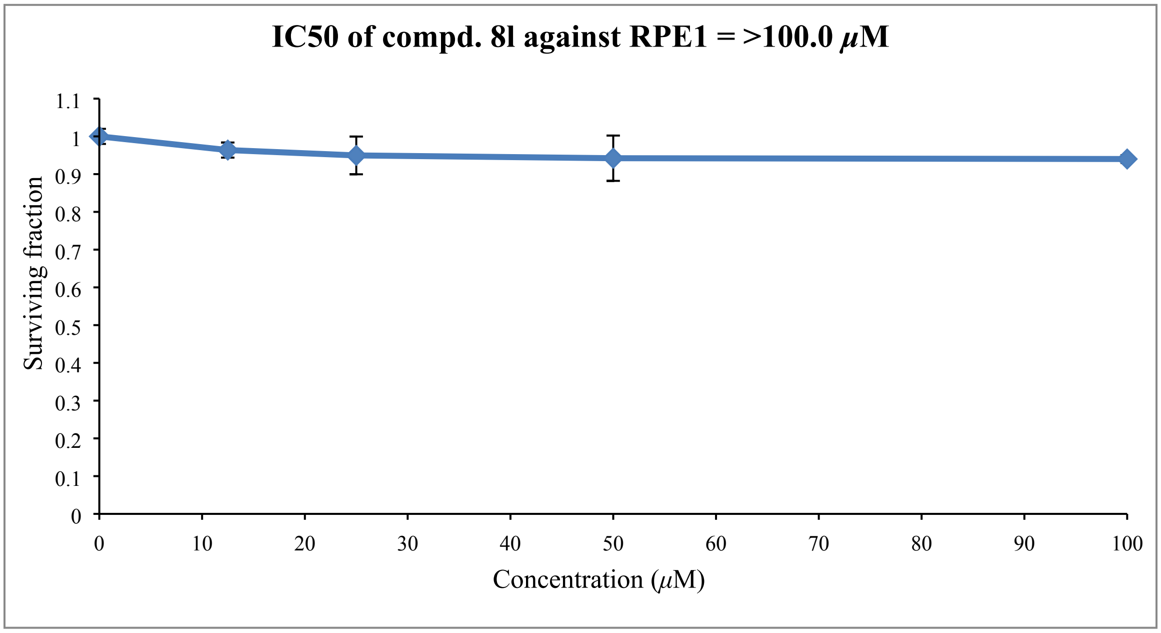

Fig. S39. Dose-response curve for the tested compounds against RPE1 (retinal pigment epithelium) cell line.

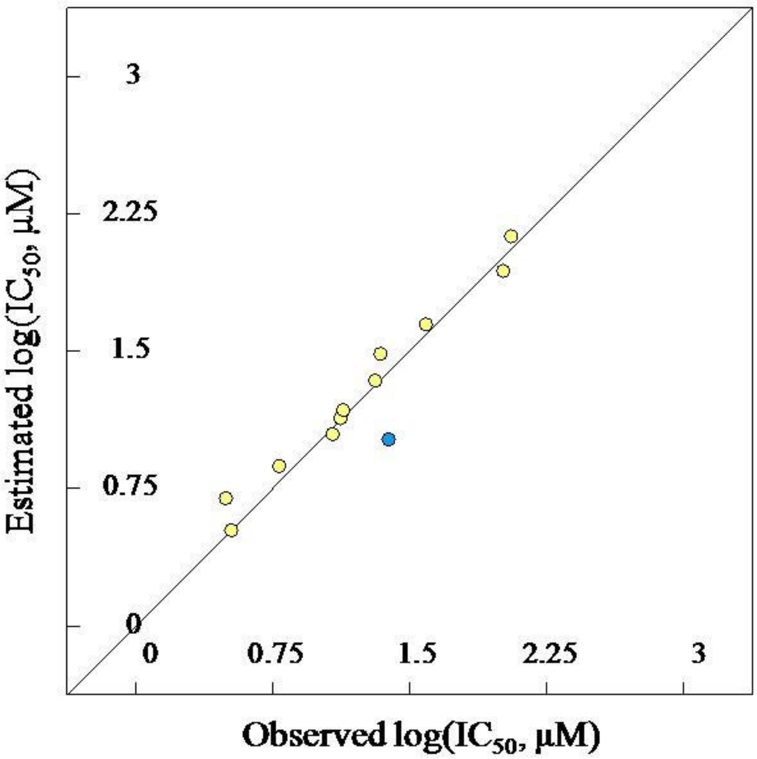

Fig. S40. BMLR-QSAR model plot of correlations representing the observed *vs.* predicted log/(IC_{50}, μM) values for the tested compounds as AChE inhibitor (compound **8d** is an outlier).

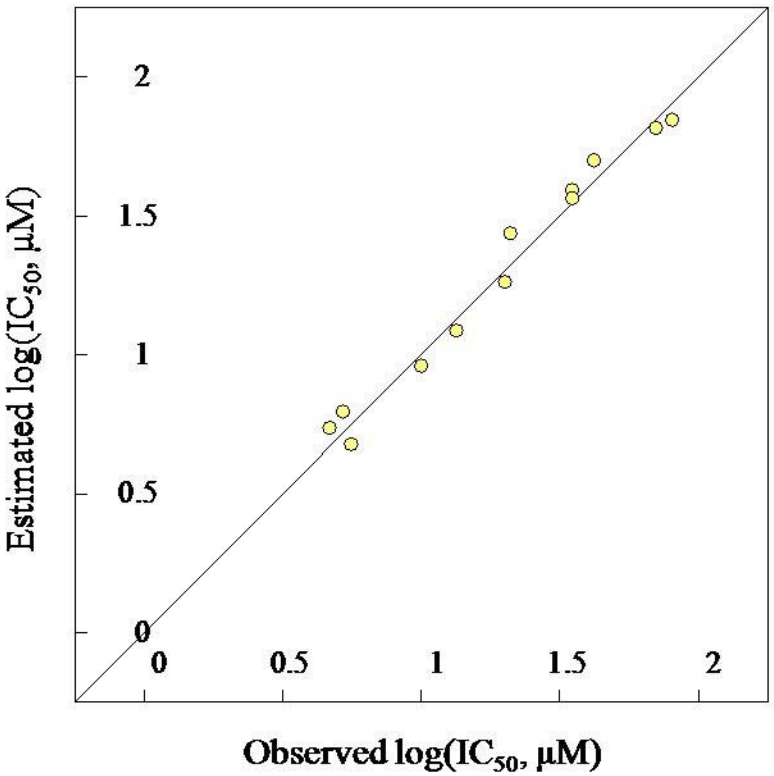

Fig. S41. BMLR-QSAR model plot of correlations representing the observed *vs.* predicted log/(IC_{50}, μM) values for the tested compounds as BChE inhibitor.

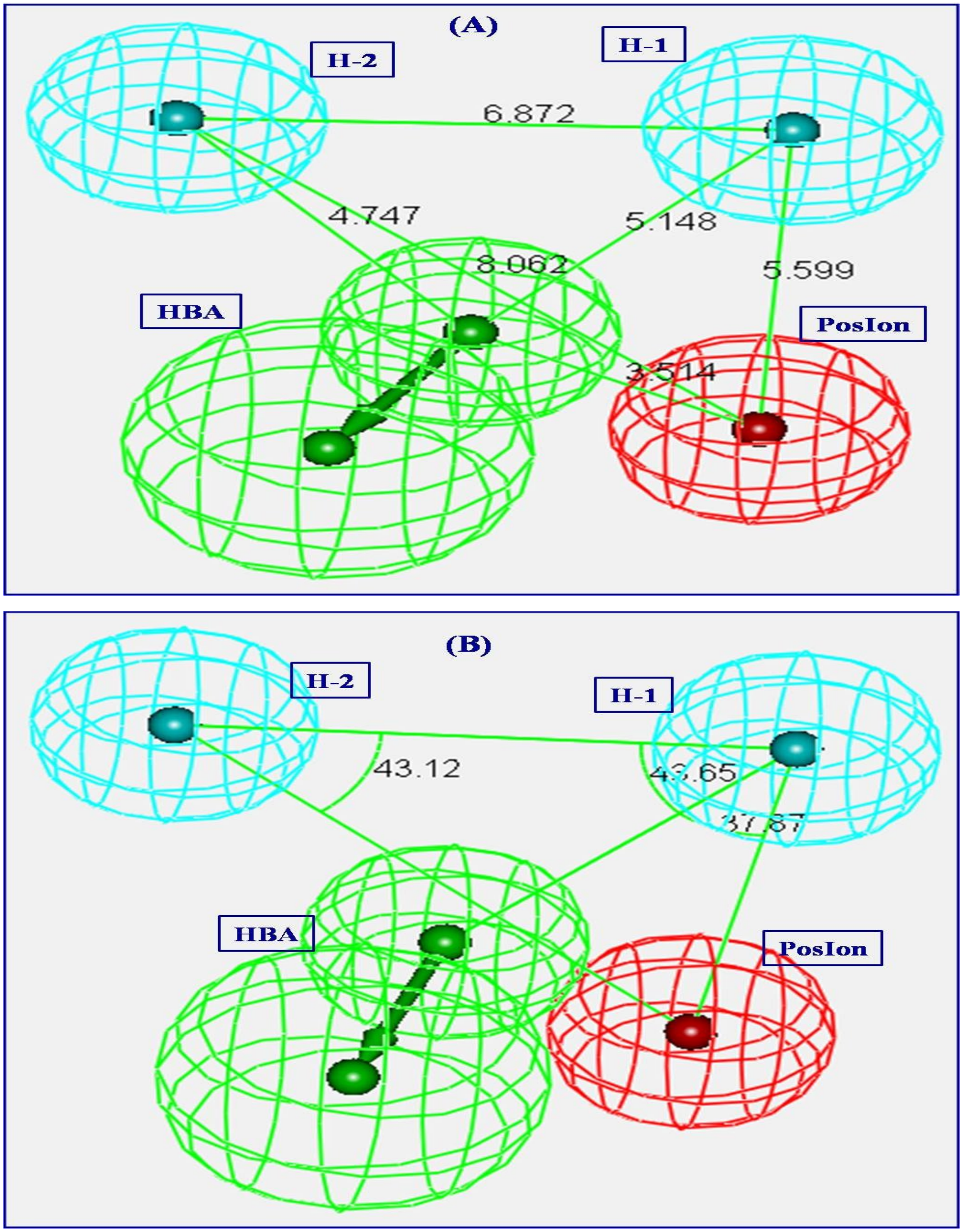

Fig. S42. (A) Constraint distances "H-1 – H-2 = 6.872, H-1 – PosIon = 5.599, H-1 – HBA = 5.148, H-2 – HBA = 4.747, H-2 – PosIon = 8.062, HBA – PosIon = 3.514 Å"; (B) Constraint angles "H-1 – H-2 – PosIon = 43.12, H-2 – H-1 – HBA = 43.65, HBA – H-1 – PosIon = 37.87 º" of the generated 3D-pharmacophore for the tested compounds **8a–l** as AChE inhibitor which contains two hydrophobics (H-1, H-2; light blue), one hydrogen bonding acceptor (HBA; green) and one positive ionizable (PosIon; red).

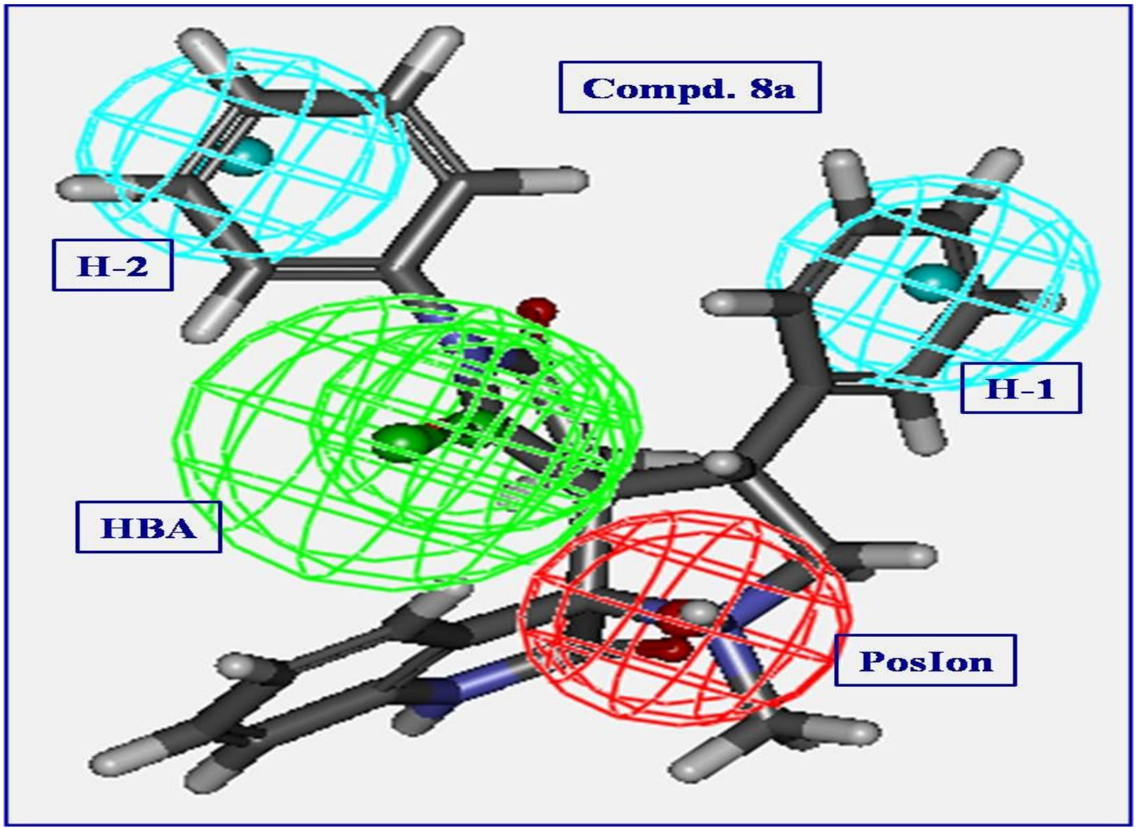
Compd. 8a
H-2
H-1
HBA
PosIon

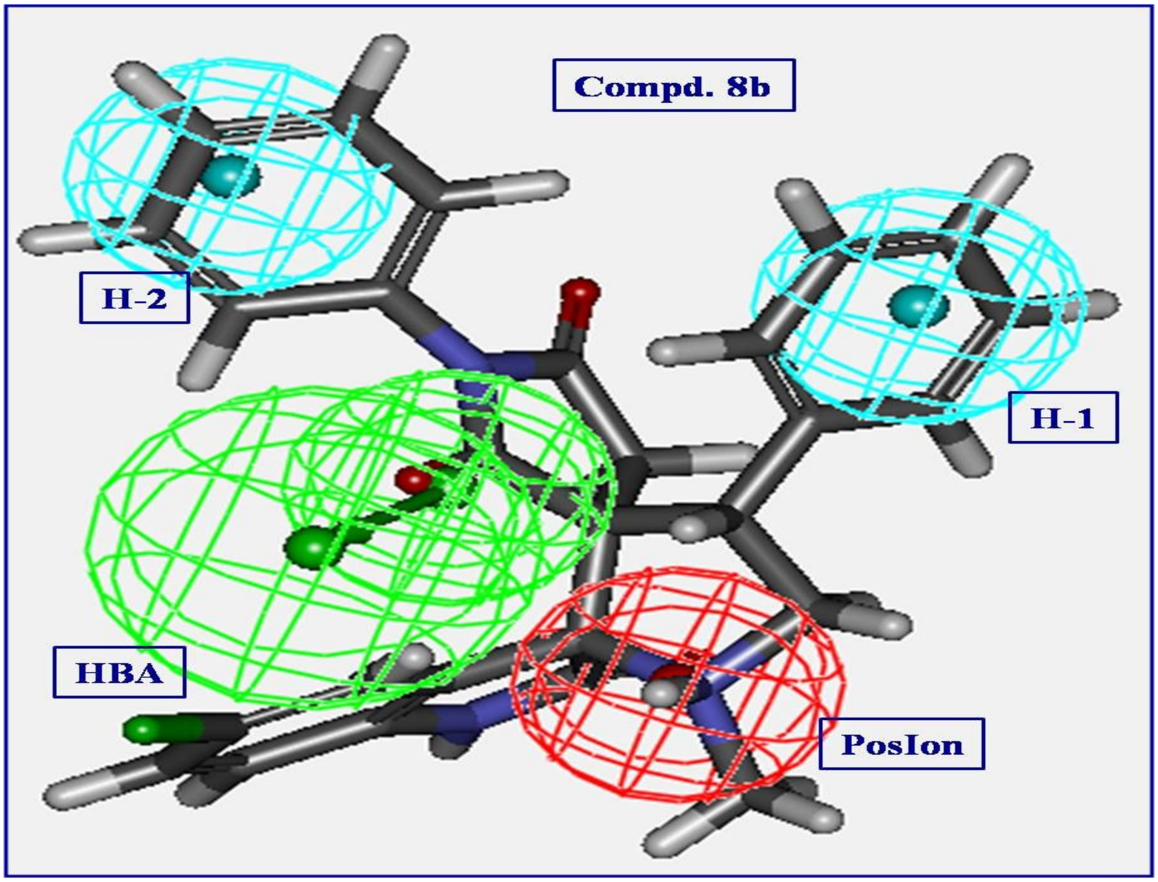
Compd. 8b
H-2
H-1
HBA
PosIon

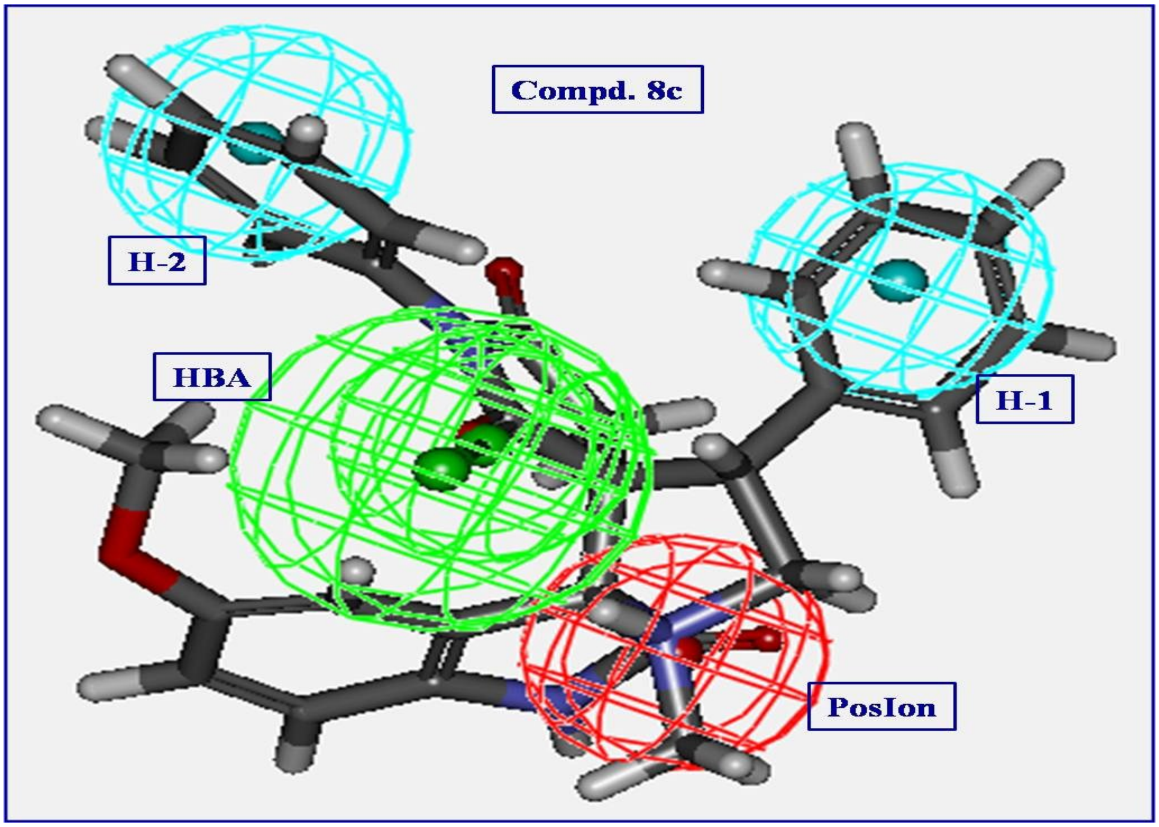
Compd. 8c
H-2
H-1
HBA
PosIon

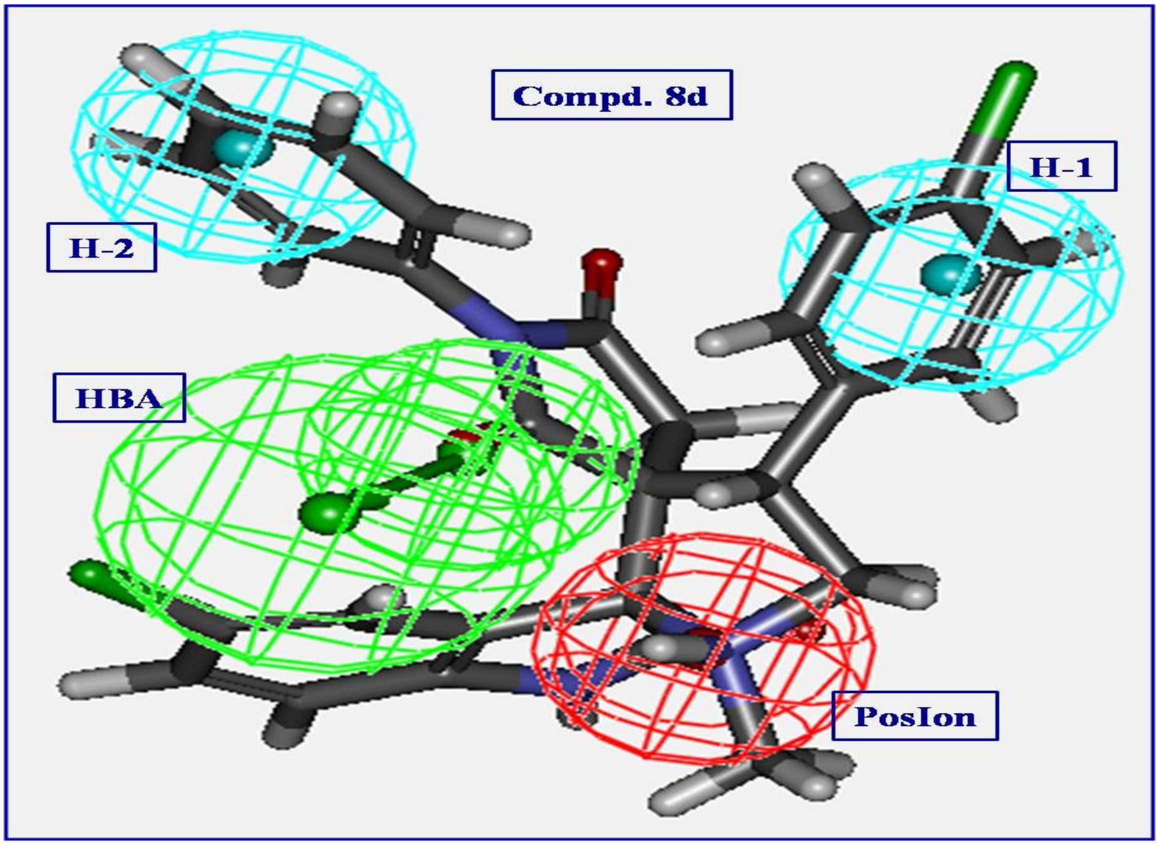
Compd. 8d
H-1
H-2
HBA
PosIon

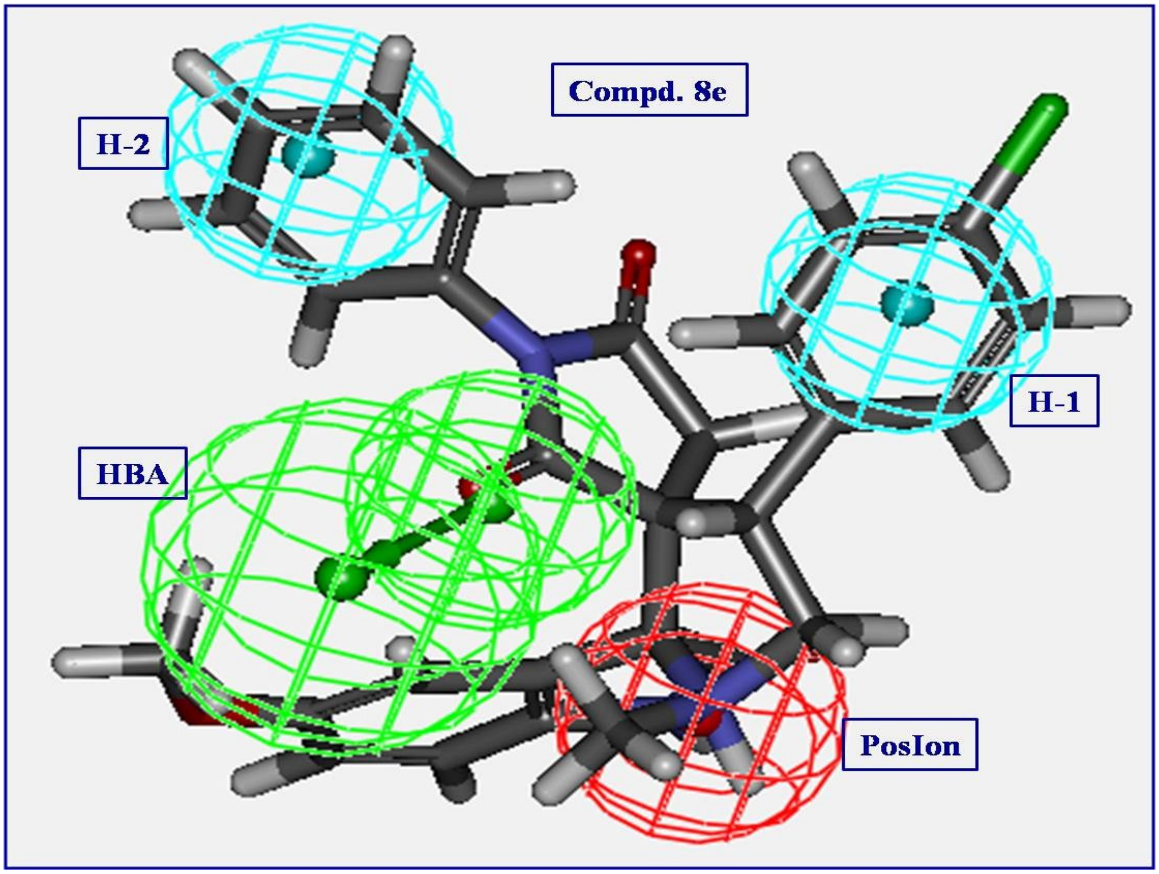
Compd. 8e
H-2
H-1
HBA
PosIon

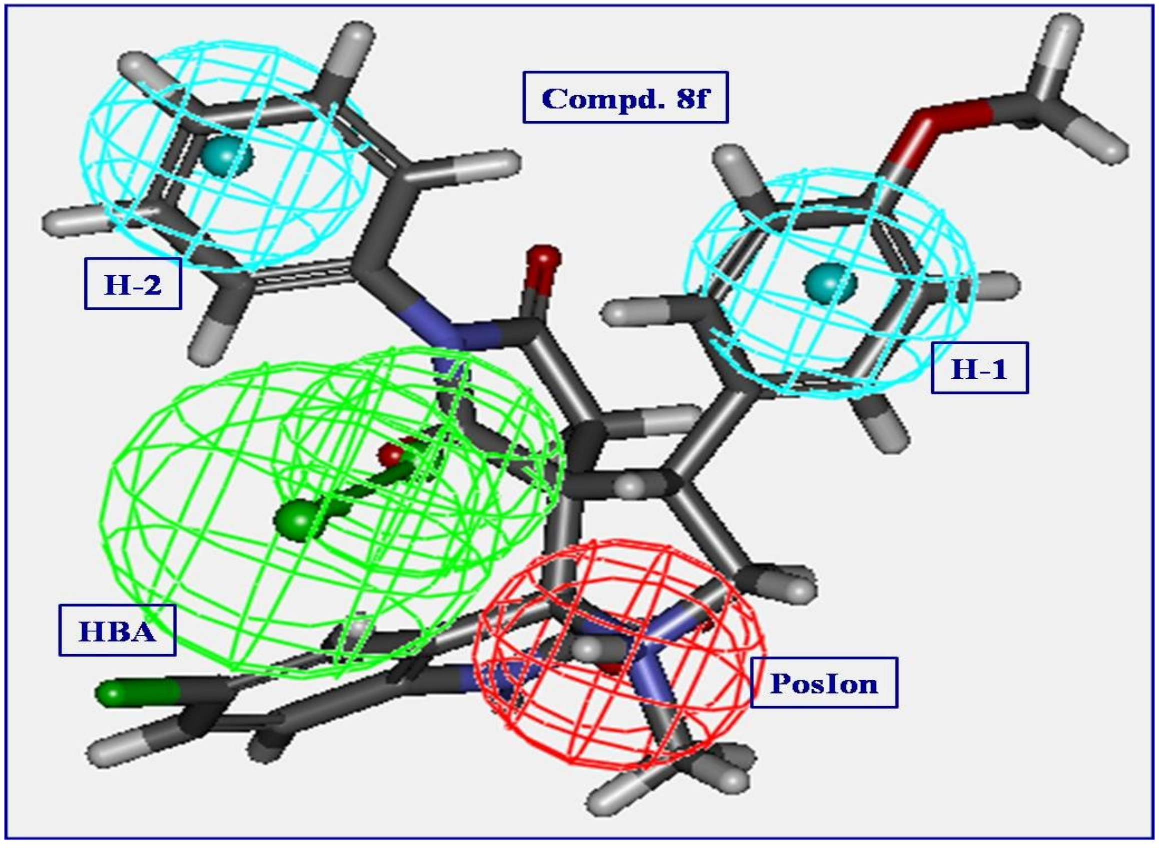
Compd. 8f
H-2
H-1
HBA
PosIon

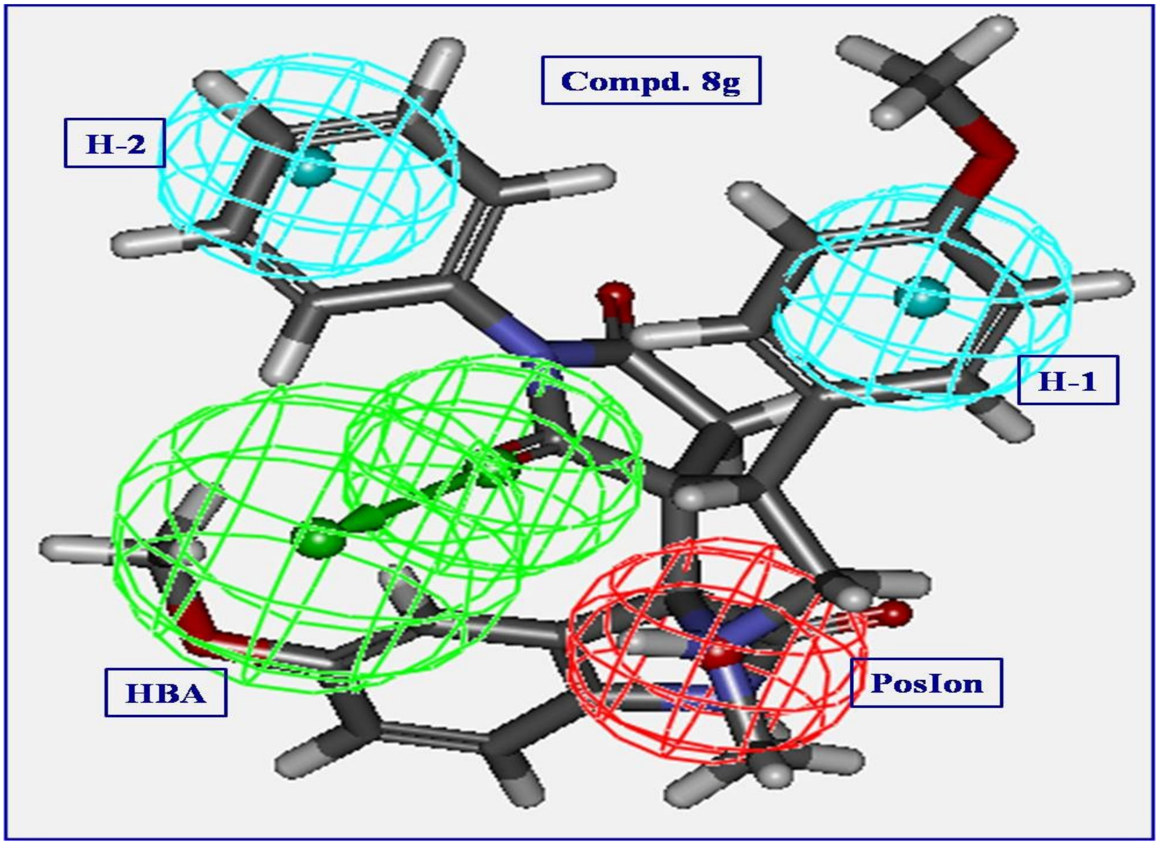
Compd. 8g
H-2
H-1
HBA
PosIon

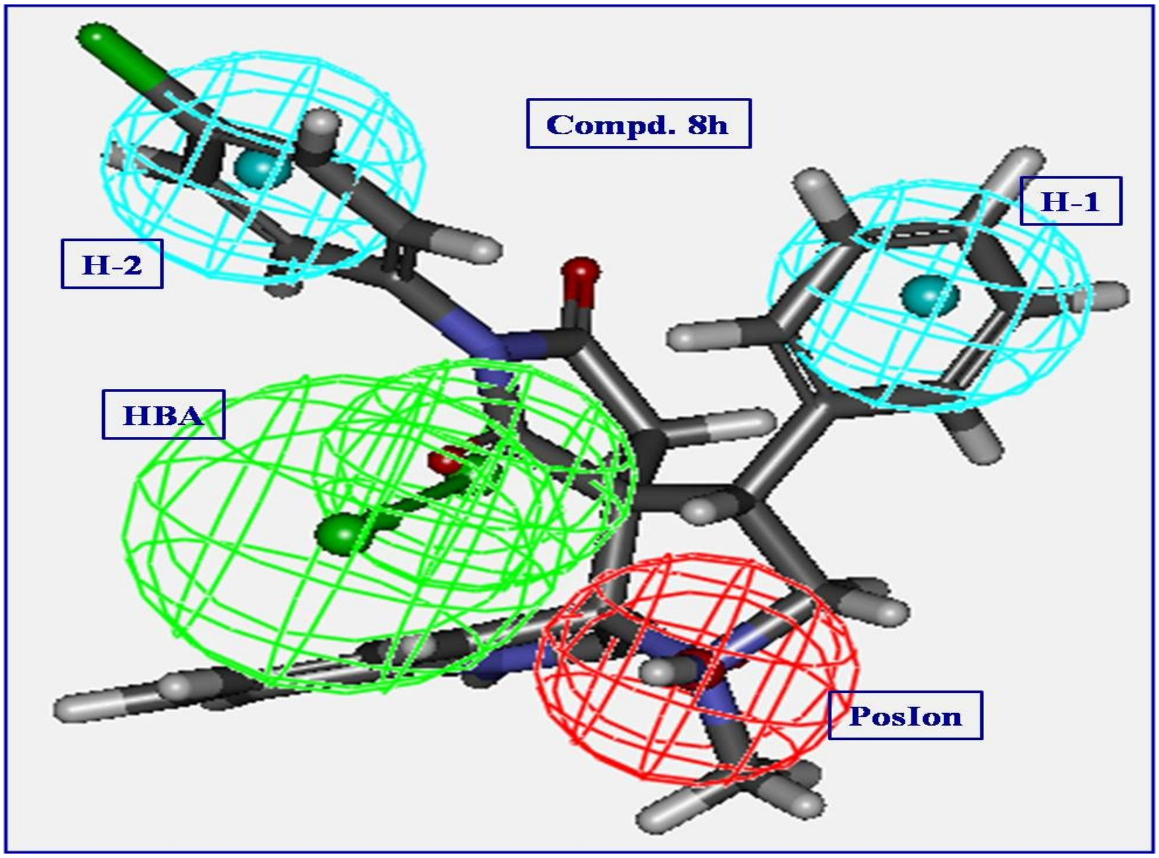
Compd. 8h
H-2
H-1
HBA
PosIon

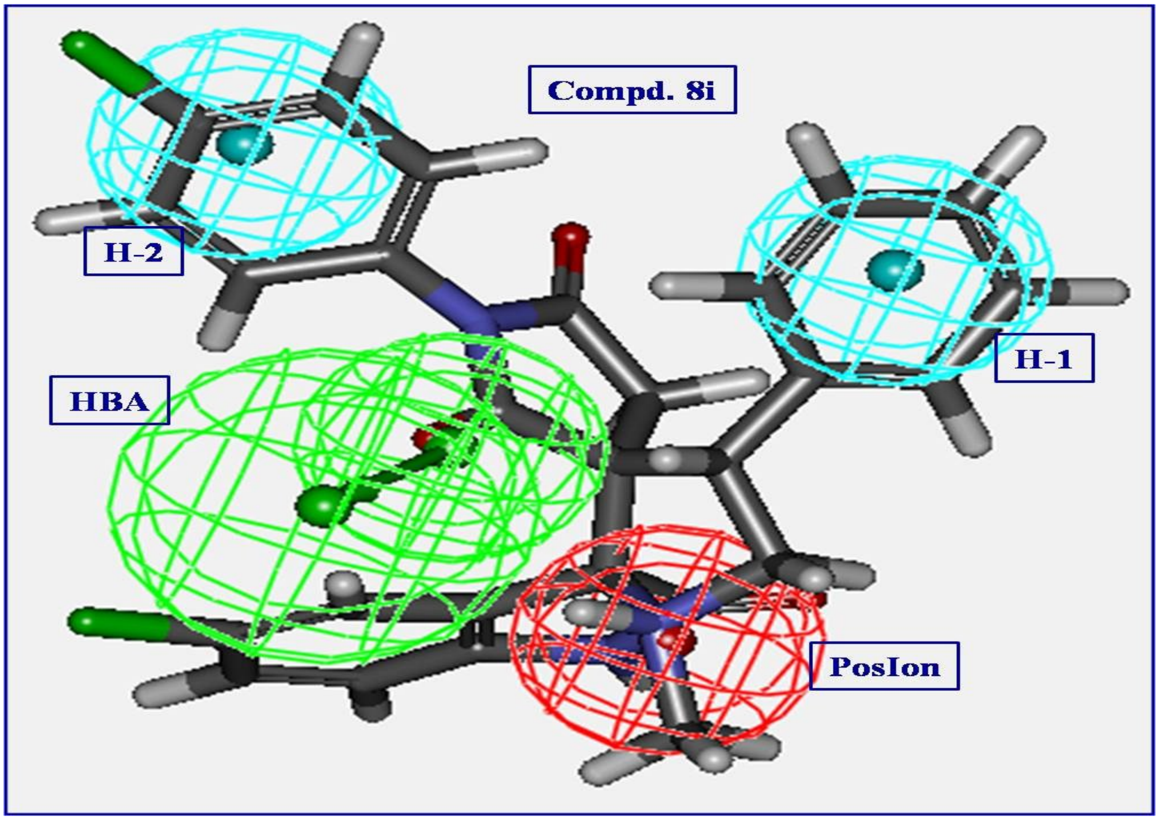
Compd. 8i
H-2
H-1
HBA
PosIon

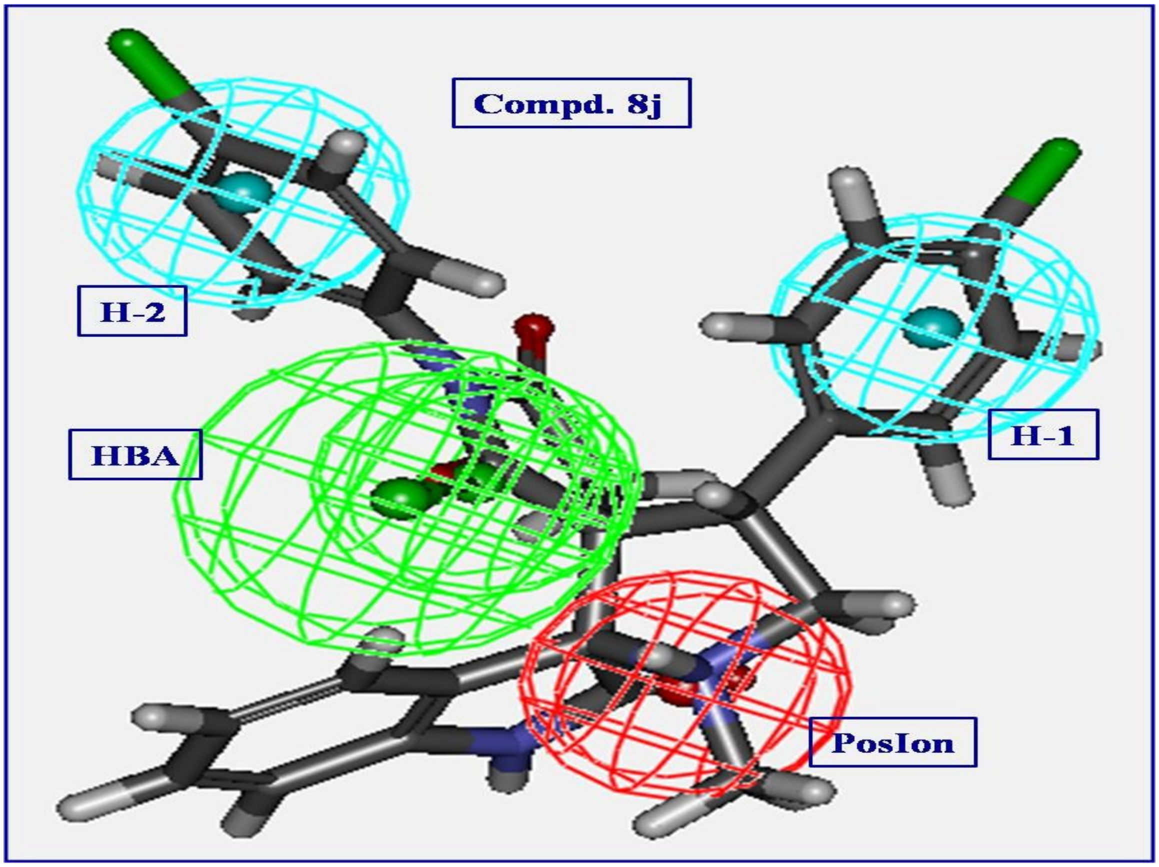
Compd. 8j
H-2
H-1
HBA
PosIon

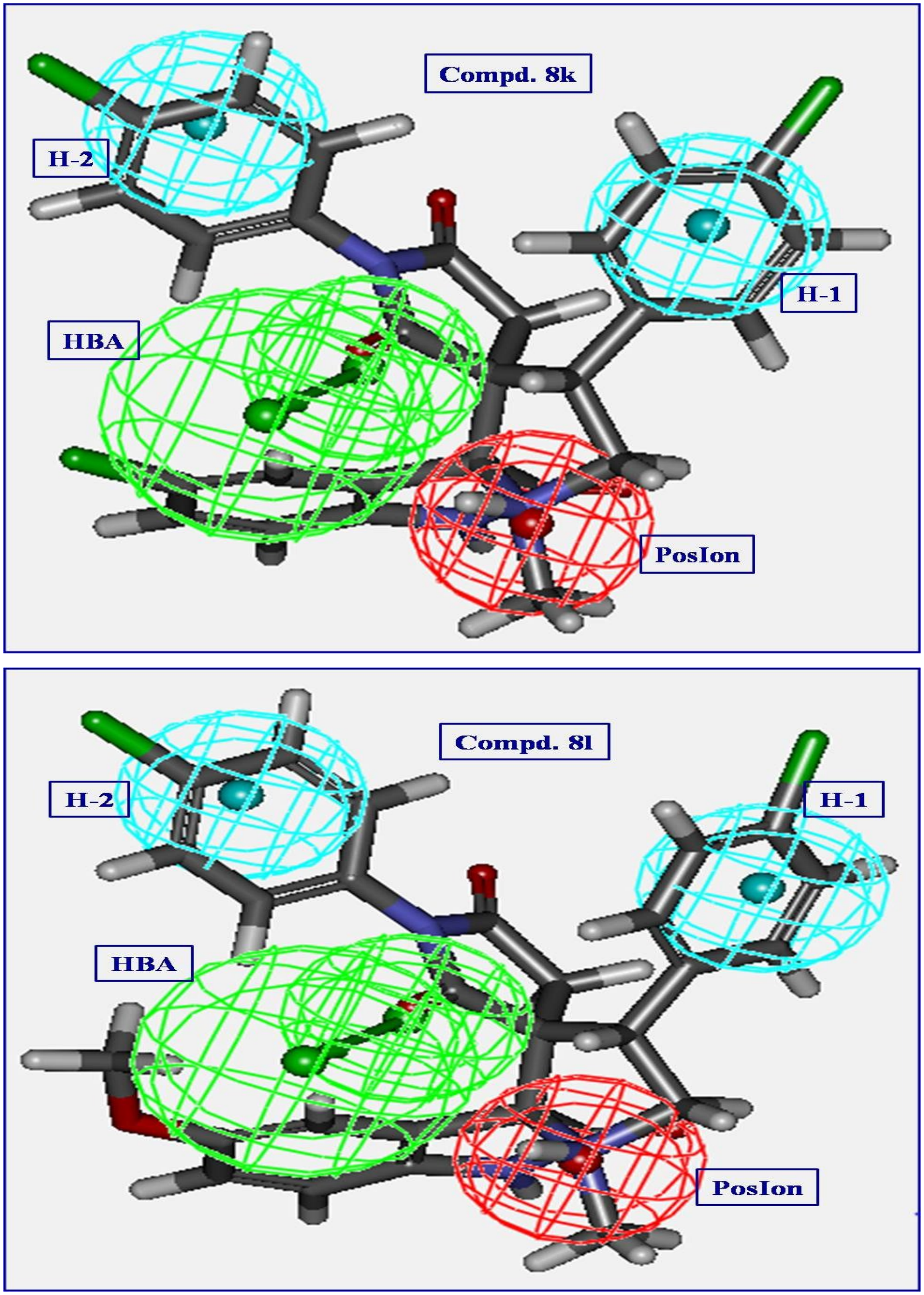

Fig. S43. 3D-pharmacophore model mapped on the tested compounds **8a–l** as AChE inhibitor.

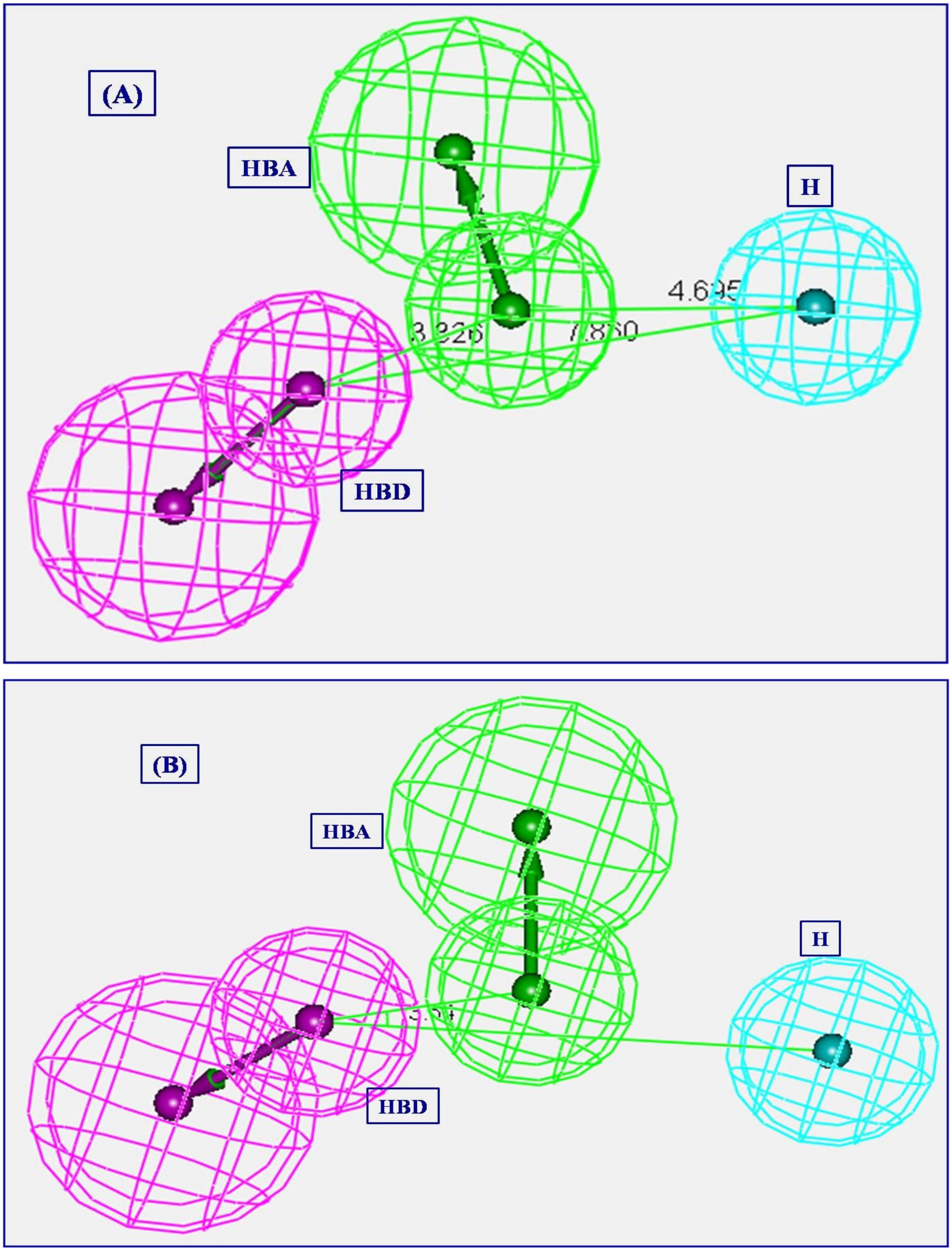

Fig. S44. (A) Constraint distances "H – HBA = 4.695, H – HBD = 7.860, HBA – HBD = 3.326 Å"; (B) Constraint angle "H – HBD – HBA = 13.64 º" of the generated 3D-pharmacophore for the tested compounds **8a–l** as BChE inhibitor which contains one hydrophobic (H; light blue), one hydrogen bonding acceptor (HBA; green) and one hydrogen bonding donor (HBD; purple).

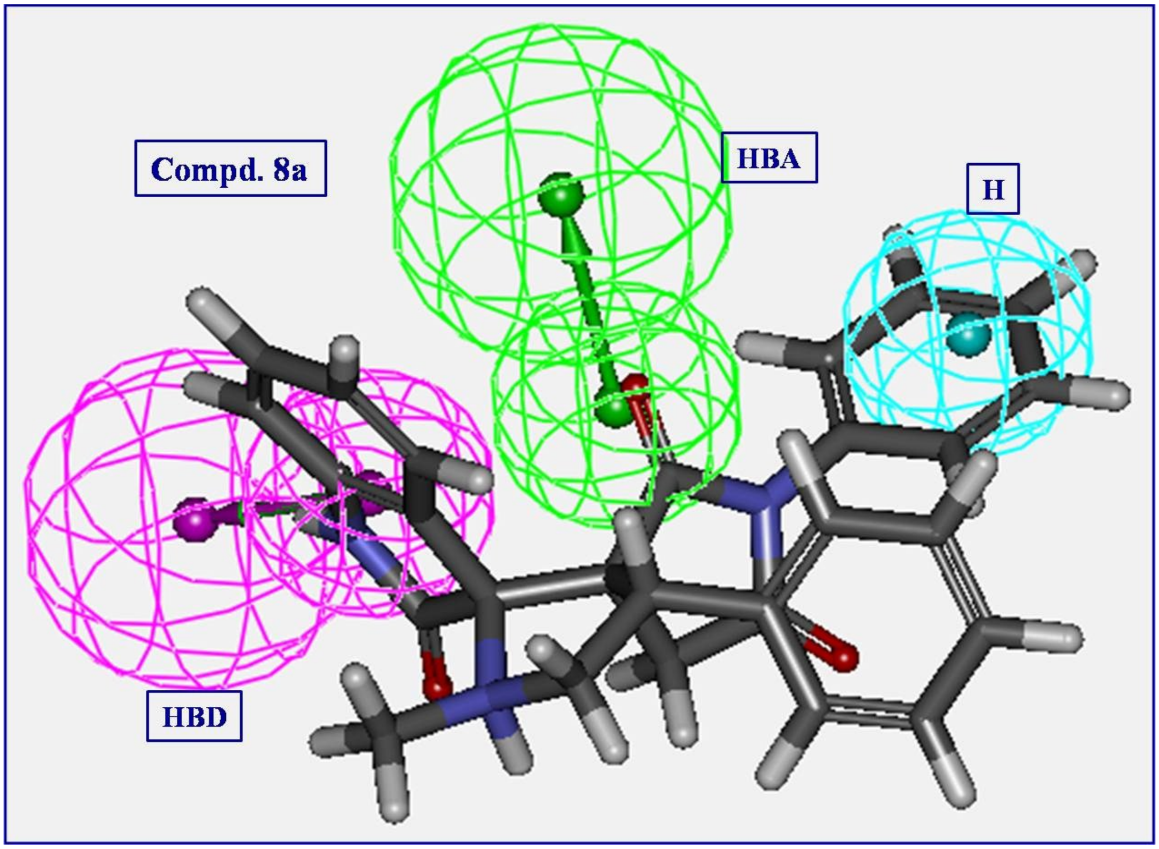
Compd. 8a
HBA
H
HBD

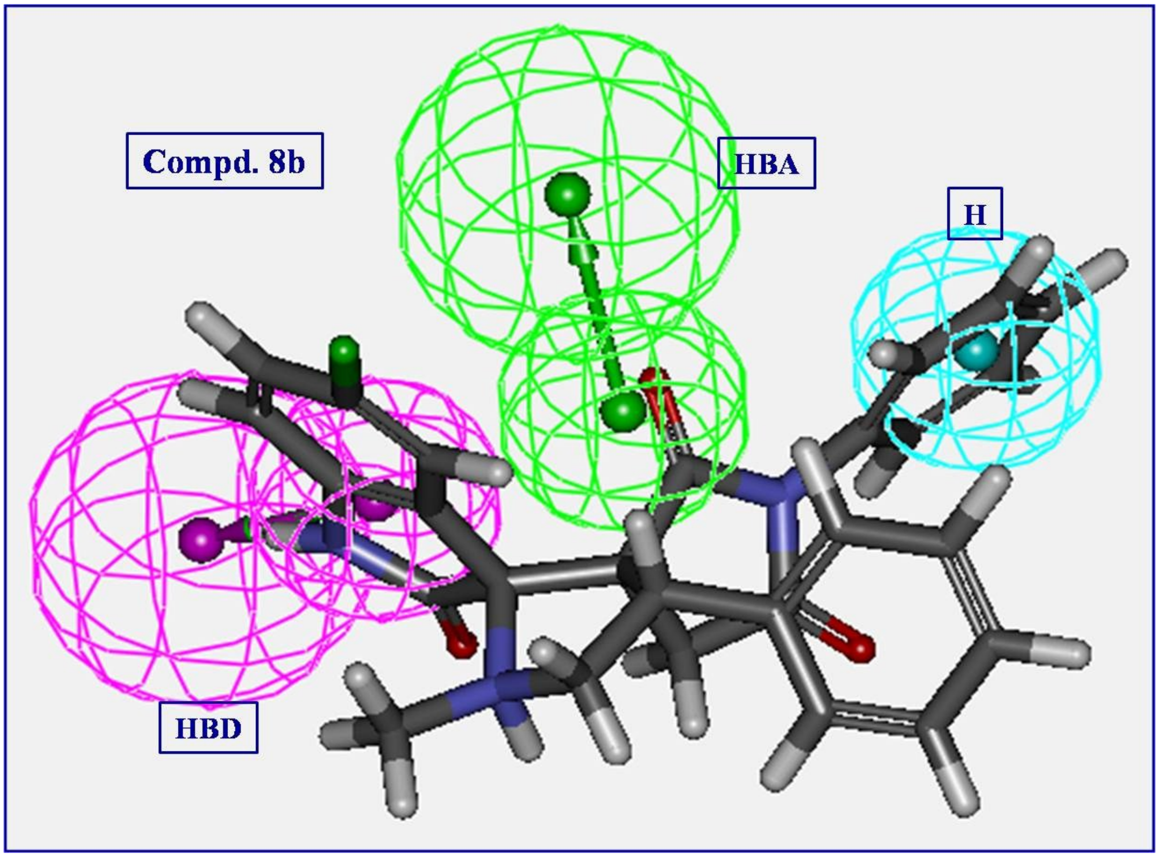
Compd. 8b
HBA
H
HBD

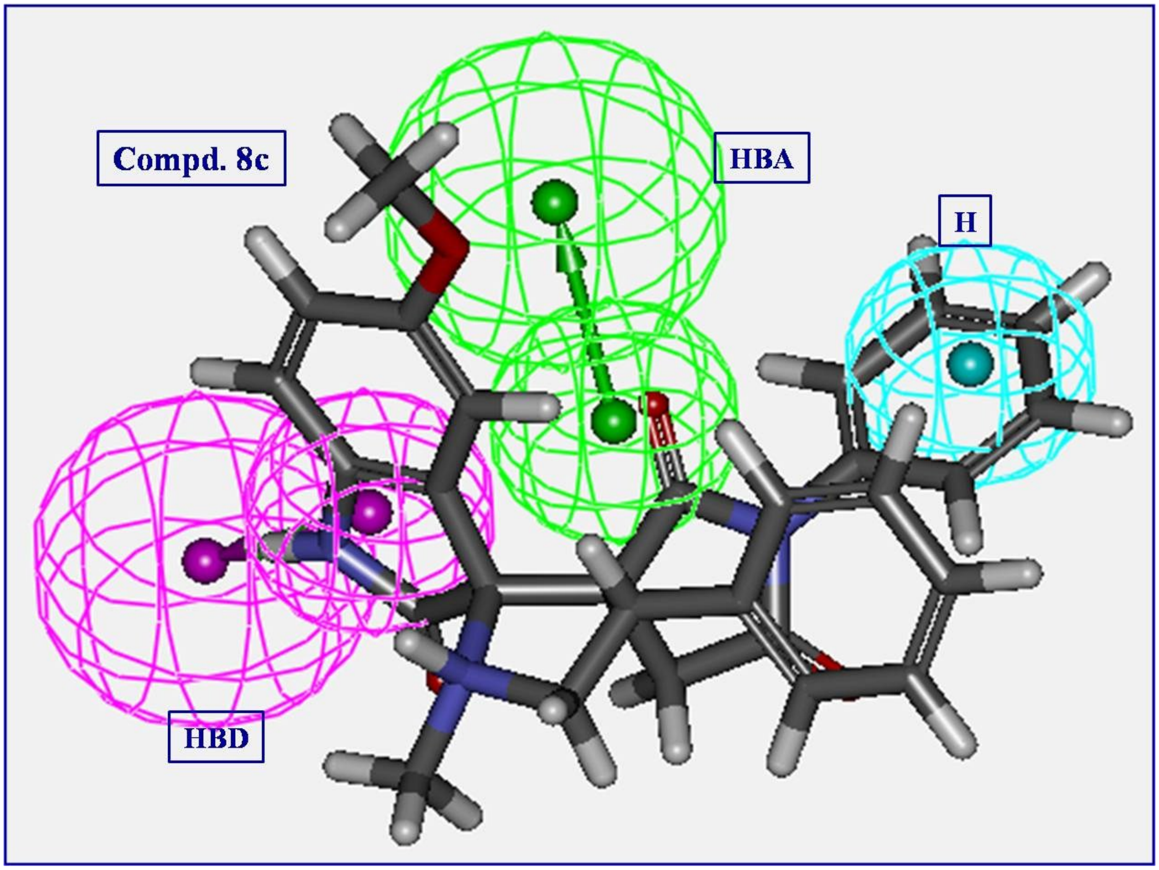
Compd. 8c
HBA
H
HBD

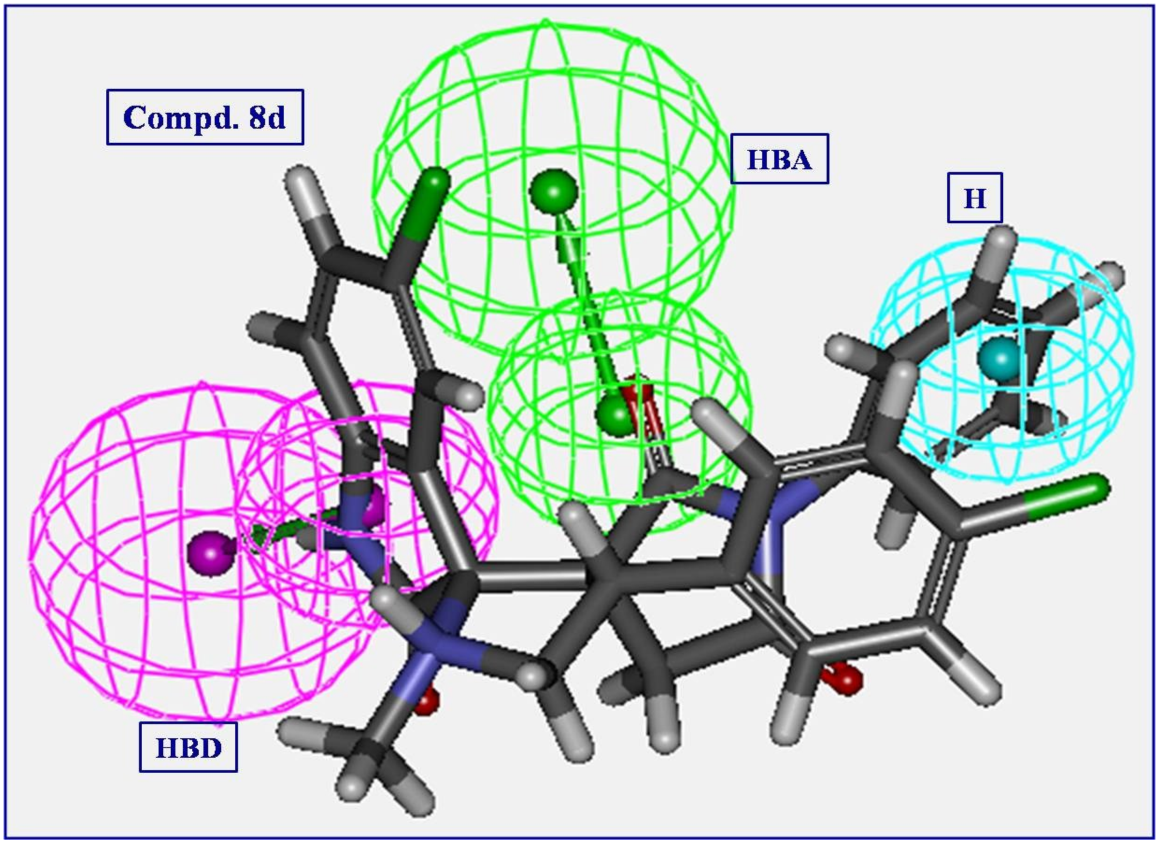
Compd. 8d
HBA
H
HBD

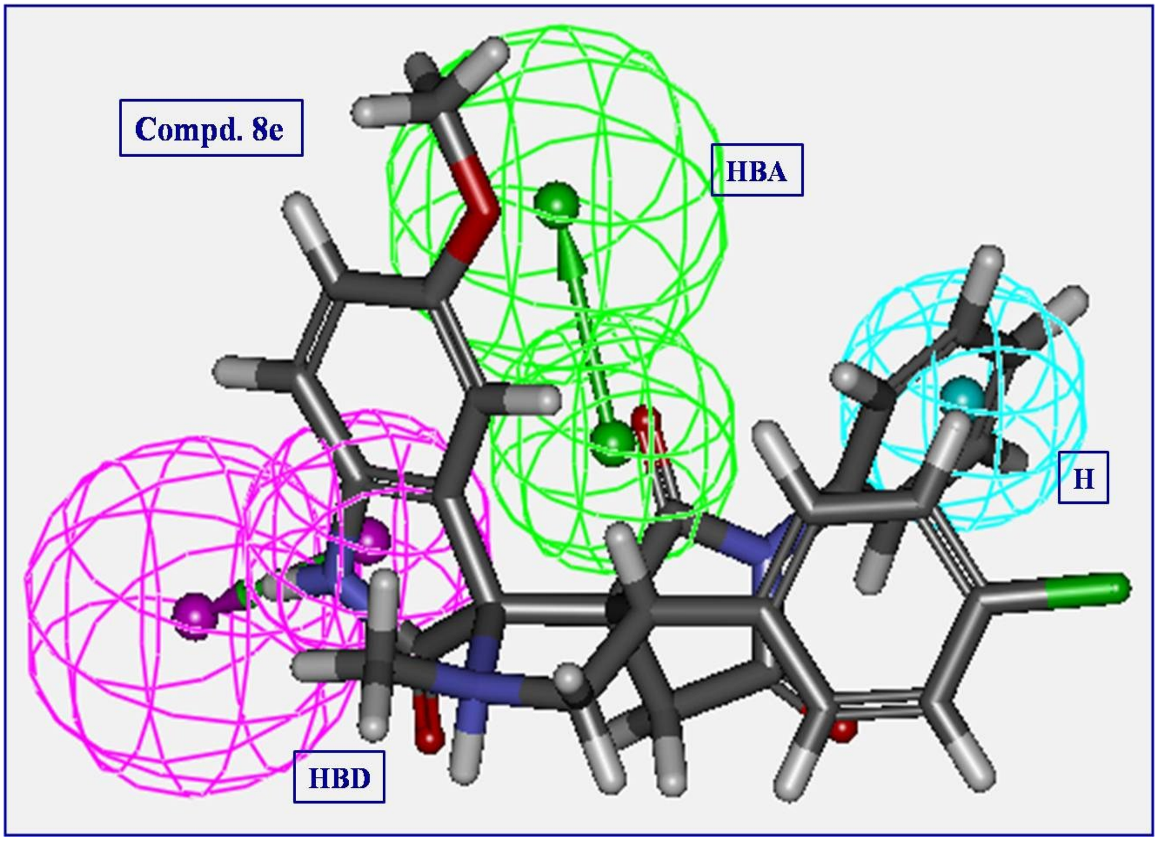
Compd. 8e
HBA
H
HBD

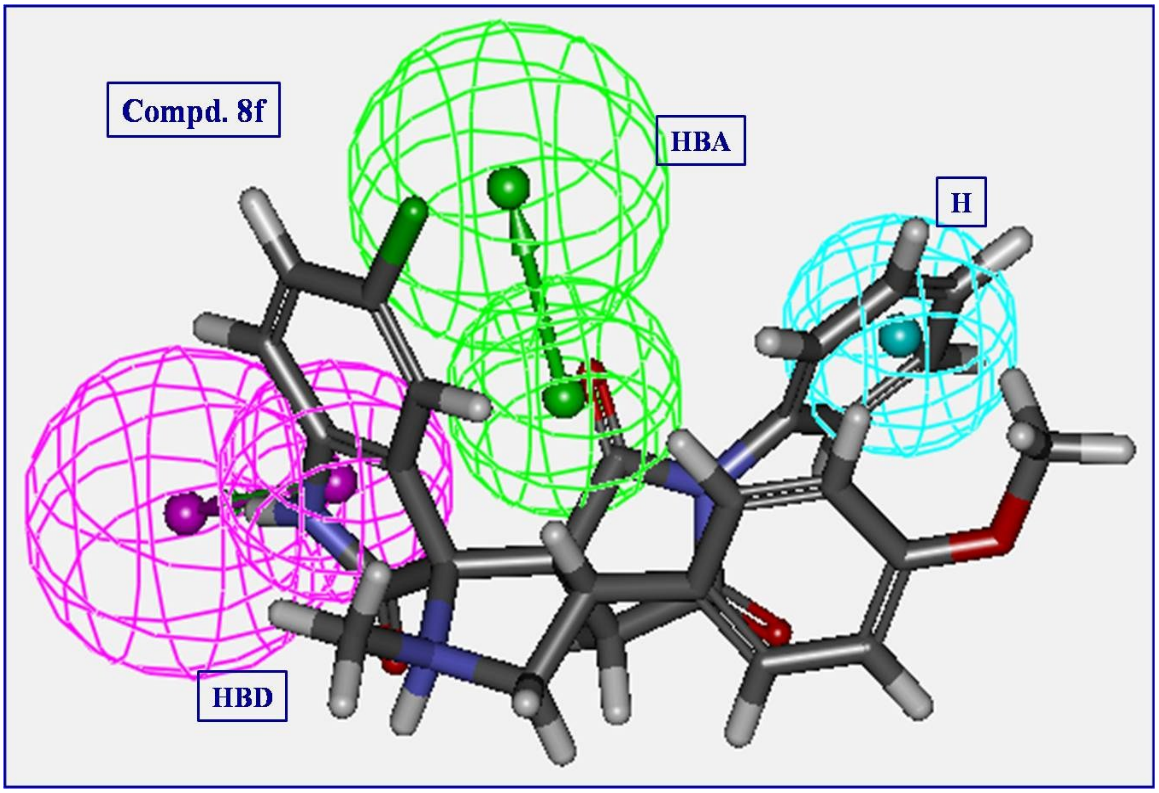
Compd. 8f
HBA
H
HBD

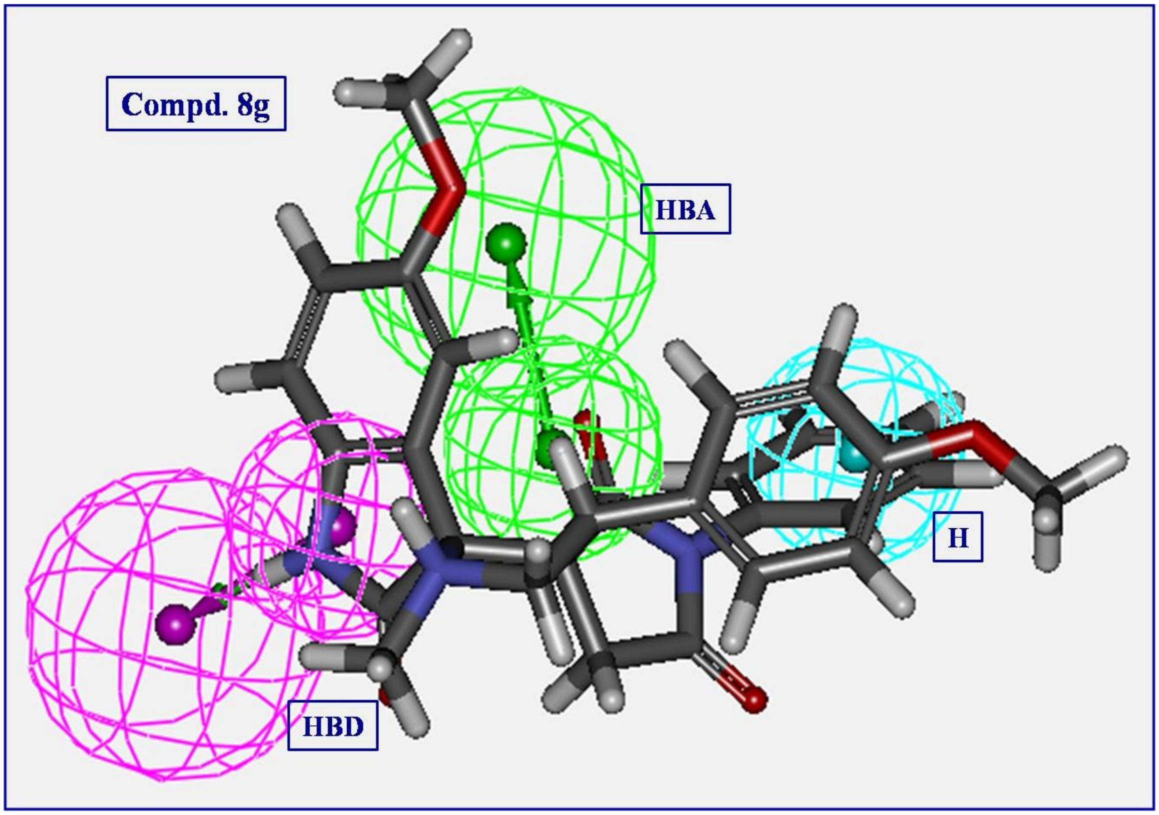
Compd. 8g
HBA
H
HBD

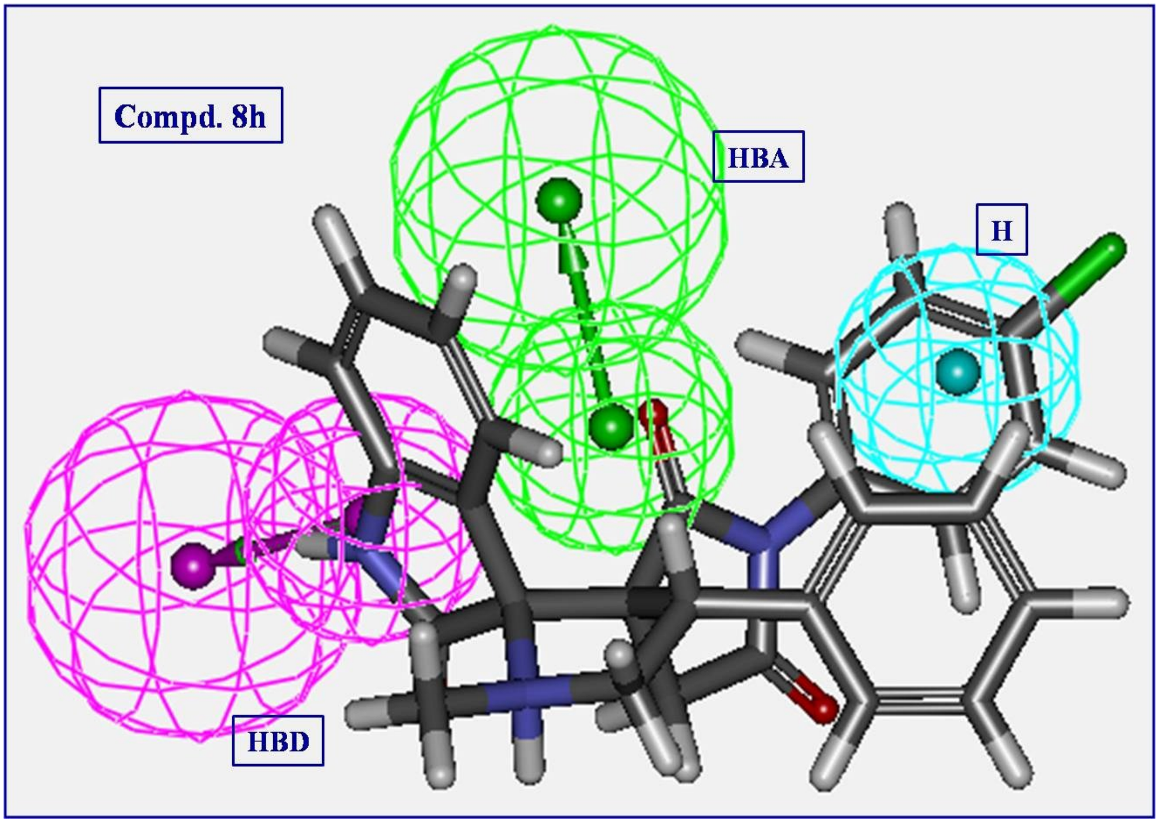
Compd. 8h
HBA
H
HBD

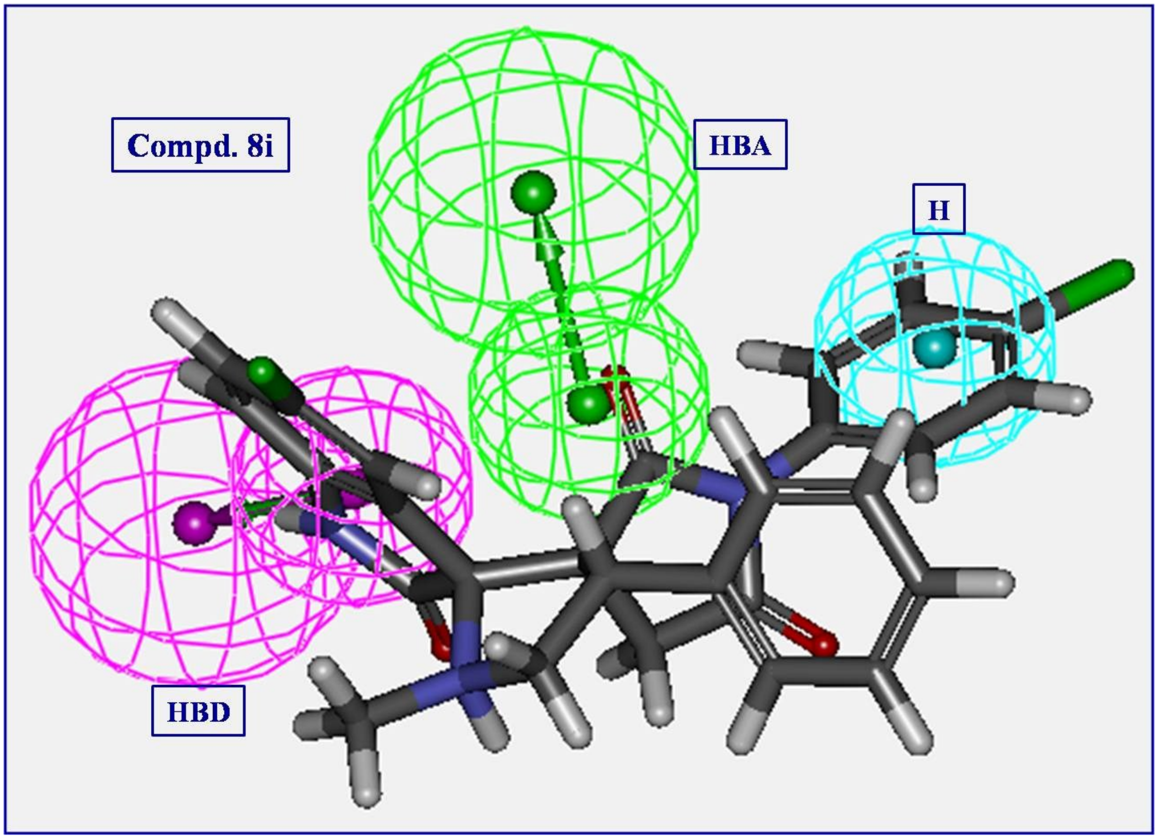
Compd. 8i
HBA
H
HBD

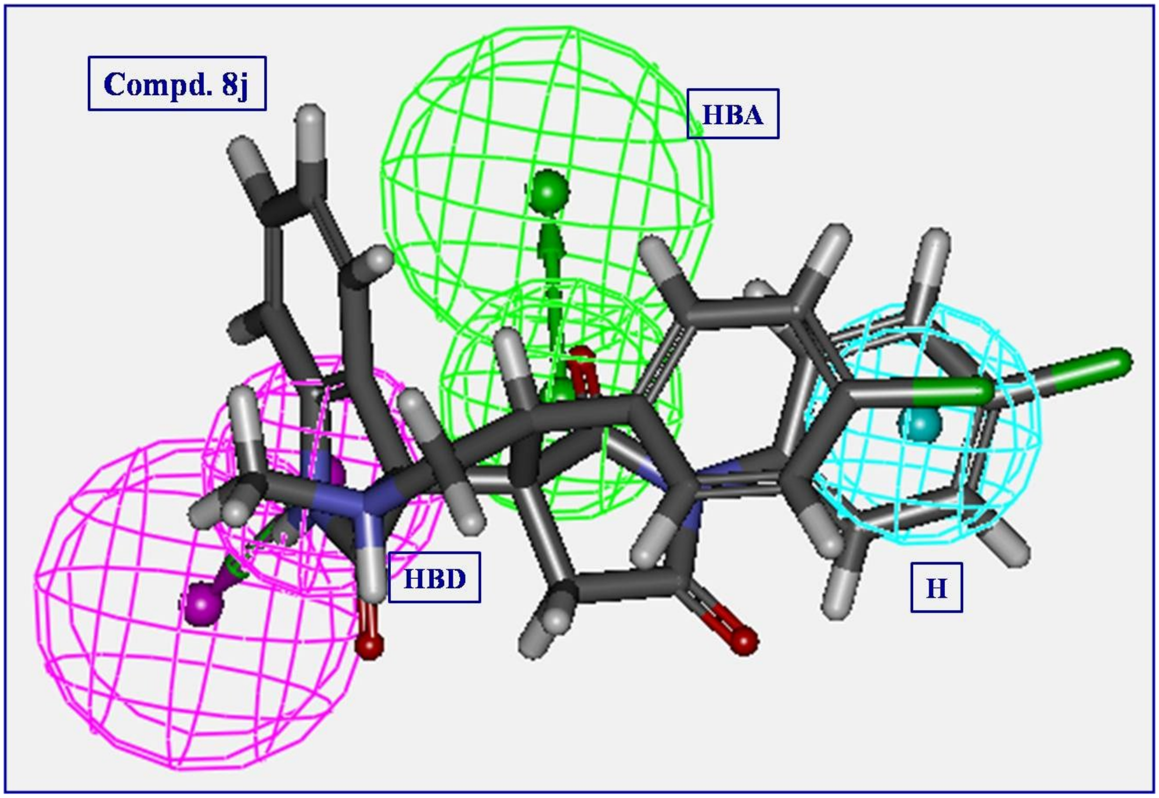
Compd. 8j
HBA
HBD
H

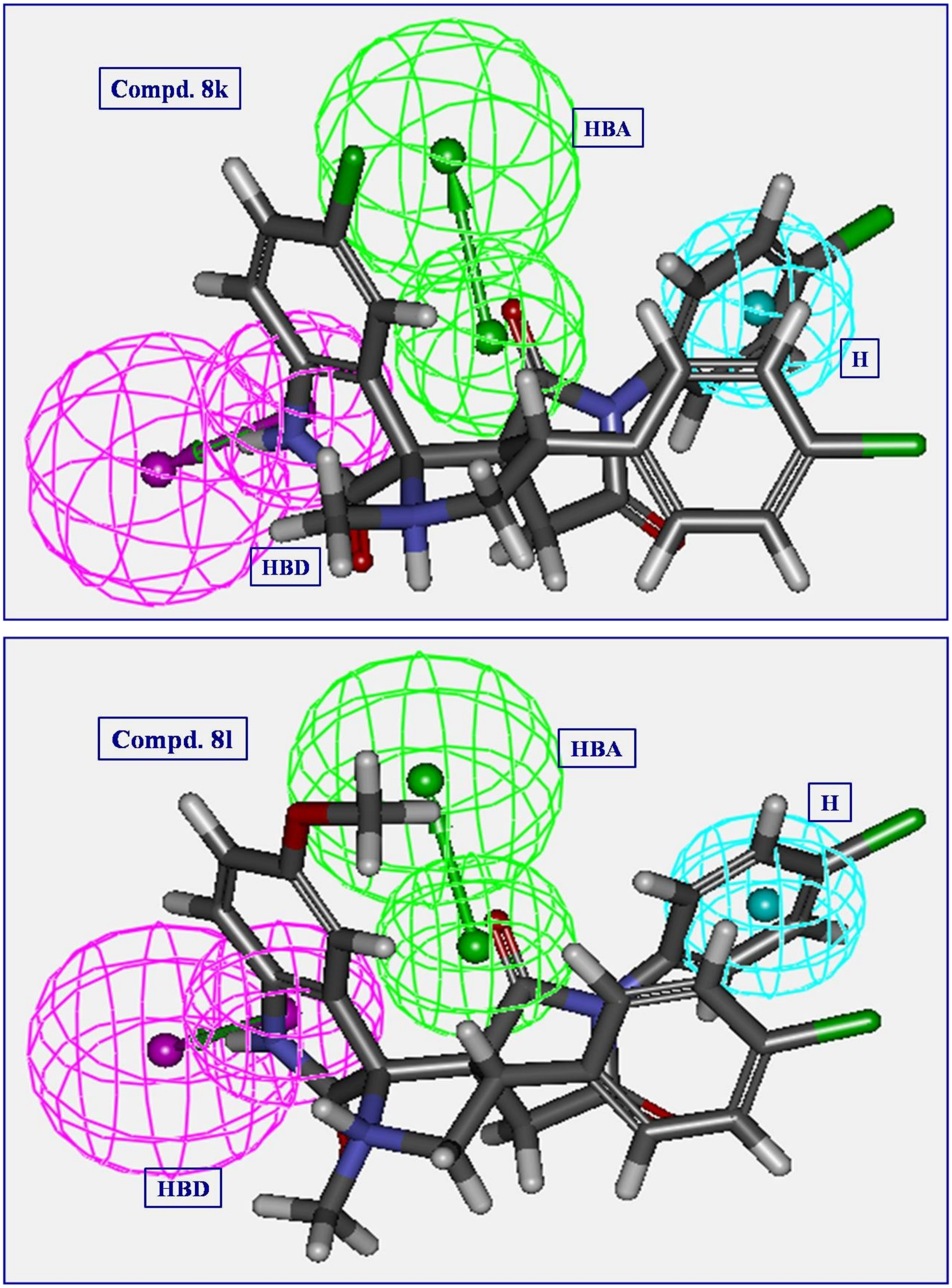

Fig. S45. 3D-pharmacophore model mapped on the tested compounds **8a–l** as BChE inhibitor.
